# Neocarzilin Inhibits Cancer Cell Proliferation via BST-2 Degradation, Resulting in Lipid Raft-Trapped EGFR

**DOI:** 10.1021/jacsau.4c00039

**Published:** 2024-05-08

**Authors:** Josef Braun, Yudong Hu, Adrian T. Jauch, Thomas F. Gronauer, Julia Mergner, Nina C. Bach, Franziska R. Traube, Stefan Zahler, Stephan A. Sieber

**Affiliations:** † TUM School of Natural Sciences, Department of Bioscience, Chair of Organic Chemistry II, Center for Functional Protein Assemblies (CPA), Technical University of Munich (TUM), Ernst-Otto-Fischer Straße 8, Garching near Munich D-85748, Germany; ‡ Department of Pharmacy, Pharmaceutical Biology, Ludwig-Maximilians-University in Munich (LMU), Butenandtstraße 5-13, Munich D-81377, Germany; § Metabolomics and Proteomics Core (MPC), Helmholtz Zentrum München GmbH German Research Center for Environmental Health, Heidemannstr. 1, Munich D-80939, Germany; ∥ Bavarian Center for Biomolecular Mass Spectrometry at Klinikum rechts der Isar (BayBioMS@MRI), Technical University of Munich (TUM), Einsteinstraße 25 Munich, D-81675, Germany; ⊥ Institute of Biochemistry and Technical Biochemistry, University of Stuttgart, Allmandring 31, Stuttgart D-70569, Germany

**Keywords:** natural products, proteomics, biological activity, mechanism of action, antitumor agents

## Abstract

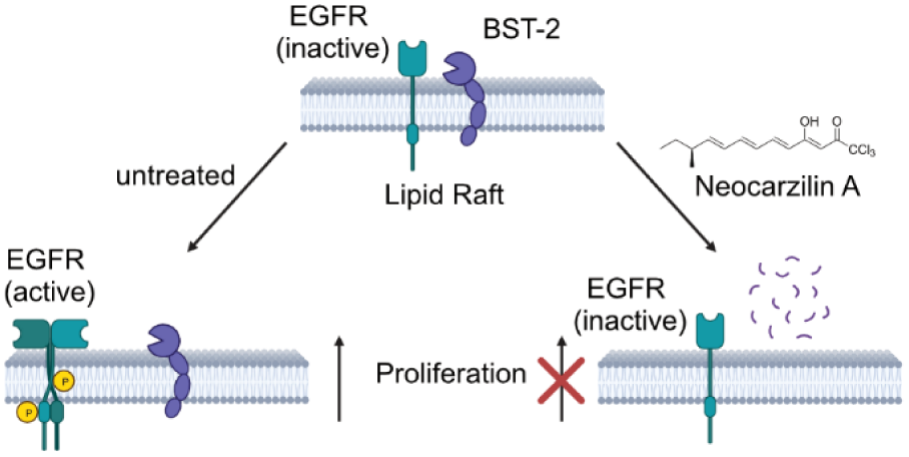

Neocarzilin (NCA) is a natural product exhibiting potent antimigratory as well as antiproliferative effects. While vesicle amine transport protein 1 (VAT-1) was previously shown to inhibit migration upon NCA binding, the molecular mechanisms responsible for impaired proliferation remained elusive. We here introduce a chemical probe closely resembling the structural and stereochemical features of NCA and unravel bone marrow stromal antigen 2 (BST-2) as one of the targets responsible for the antiproliferative effect of NCA in cancer cells. The antiproliferative mechanism of NCA was confirmed in corresponding BST-2 knockout (KO) HeLa cells, which were less sensitive to compound treatment. Vice versa, reconstitution of BST-2 in the KO cells again reduced proliferation upon NCA addition, comparable to that of wild-type (wt) HeLa cells. Whole proteome mass spectrometric (MS) analysis of NCA-treated wt and KO cancer cells revealed regulated pathways and showed reduced levels of BST-2 upon NCA treatment. In-depth analysis of BST-2 levels in response to proteasome and lysosome inhibitors unraveled a lysosomal degradation path upon NCA treatment. As BST-2 mediates the release of epidermal growth factor receptor (EGFR) from lipid rafts to turn on proliferation signaling pathways, reduced BST-2 levels led to attenuated phosphorylation of STAT3. Furthermore, fluorescence microscopy confirmed increased colocalization of EGFR and lipid rafts in the presence of NCA. Overall, NCA represents a versatile anticancer natural product with a unique dual mode of action and unconventional inhibition of proliferation via BST-2 degradation.

## Introduction

Natural products represent a rich source of bioactive molecules with widespread applications in medicine.^1^ Their structural diversity is evolutionarily optimized to address a wealth of cellular targets and facilitate numerous modes of action (MoA). Advances in target identification via chemical proteomics have revolutionized our knowledge in the breadth of the natural product MoA scope, hallmarked also by unconventional strategies such as addressing multiple proteins, overactivation of enzyme turnover, and molecular glues leading to proteasomal protein degradation.^2−5^ Especially, the latter discovery is groundbreaking as it highlights the power of evolution to design compounds combining several functional traits.

In fact, a wealth of natural products still lacks a firm functional characterization, and while in some cases a single target has been discovered, it often cannot consolidate the full MoA, requiring further in-depth studies. Given the unique inspiration for drug development, these studies are worthwhile endeavors.

**Figure 1:**
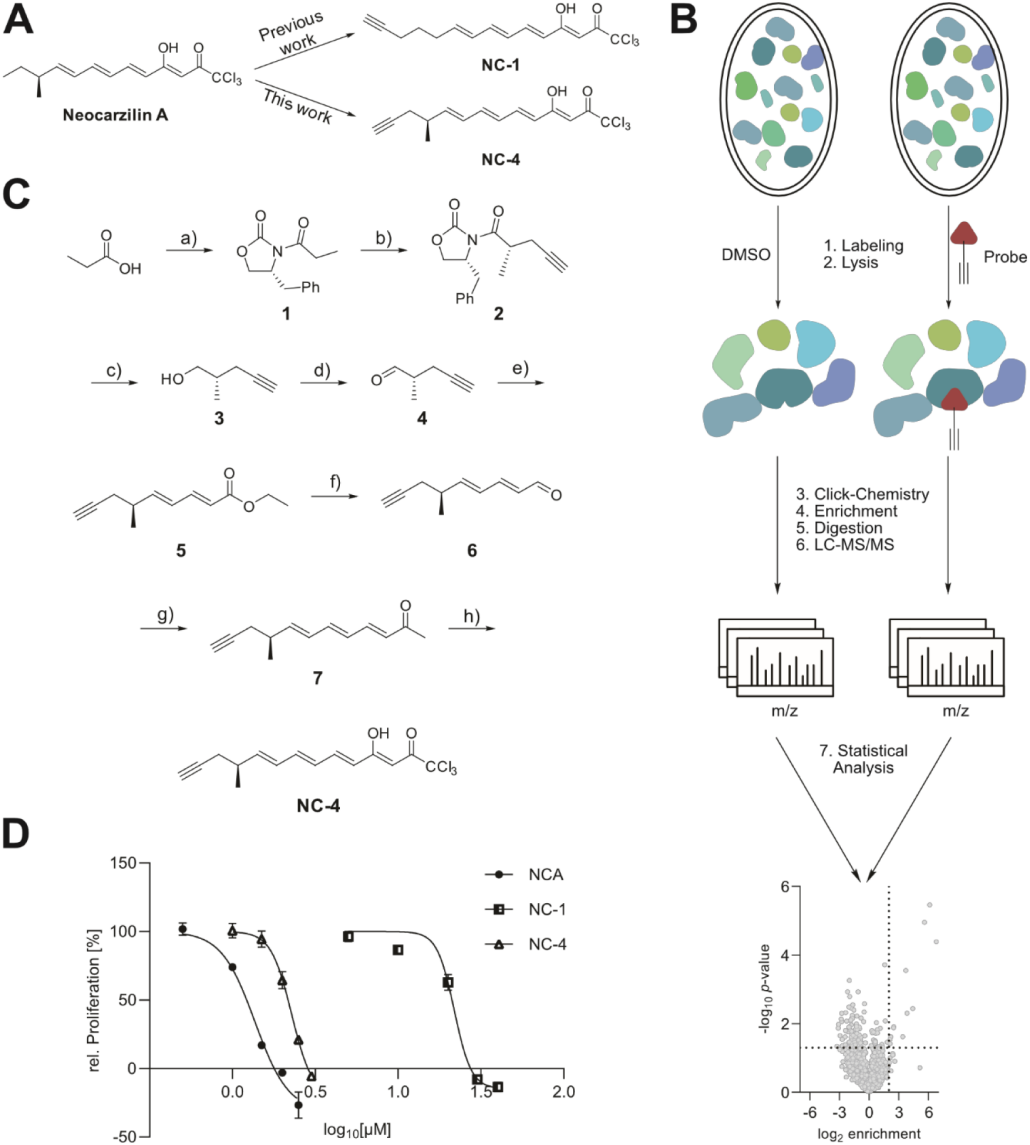
Design, synthesis, and labeling with the improved probe NC-4. (A) Chemical structure of the natural product neocarzilin A and probes NC-1^6^ and NC-4. (B) Schematic overview of an MS-based *in situ* ABPP experiment. (C) Synthesis of probe NC-4: (a) 1. NEt_3_, pivolyl chloride; 2. LiCl, (*R*)-4-benzyl-2-oxazolidinone; (b) 1. Diisopropylamine, *n*BuLi; (2) DMPU, propargyl bromide; (c) 1. MeOH, LiBH_4_; 2. NaOH_aq_; (d) DMSO, (COCl)_2_, NEt_3_; (e) ethyl (*E*)-4-(diethoxyphosphoryl)but-2-enoate, LiHMDS; (f) 1. DIBAL-H, MnO_2_; (g) 1-(triphenylphosphoraniliden)-2-propanone; (h) 1. LiHMDS; 2. Trichloroacetic anhydride. (D) The antiproliferative activity of NCA, NC-1, and NC-4 in HeLa wt cells was measured by a crystal violet staining assay. Cells were treated with the respective compounds at the indicated concentrations for 72 h. Data are presented as the mean ± SEM (*n* = 3).

We recently studied the MoA of the anticancer natural product neocarzilin A (NCA), synthesized by *Streptomyces carzinostaticus* (Figure 1A).^6,7^ NCA exhibits potent antiproliferative (IC_50_ = 0.4 μM) and antimigratory (50% reduction at 1.5 μM) effects on cancer cells^6^ and features a characteristic trichloromethylketone moiety indicative of a covalent binding mode. In order to decipher its targets, we synthetically equipped a simplified version of NCA with an alkyne moiety for target protein enrichment via activity-based protein profiling (ABPP).^8,9^ For the ease of synthesis, the terminal methyl group, including its *S*-configured stereocenter, was omitted, leading to a probe with almost retained antimigratory activity but a 60-fold drop in antiproliferative activity compared to the parent NCA. Treatment of viable MDA-MB-231 cells with the probe, followed by lysis and enrichment of labeled proteins via click ligation to biotin azide, enabled the identification of targets with mass spectrometry (MS) (Figure 1B).^10,11^ The vesicle amine transport protein 1 (VAT-1) was discovered as a prominent hit, and its role in the antimigratory effect of NCA was confirmed. However, VAT-1 could not explain the potent antiproliferative effect of NCA, highlighting the need for an NCA probe containing the native stereocenter. In fact, the synthesis of an NCA derivative with an inverted *R*-stereocenter resulted in a 3-fold drop of antiproliferative activity.

We here address this limited knowledge about the full complement of targets by the stereoselective synthesis of a novel NCA probe bearing the *S*-configured methyl moiety, which retains both potent antimigratory and antiproliferative effects. Chemical proteomic labeling of HeLa cells revealed bone marrow stromal antigen 2 (BST-2), a protein needed for antiviral defense and with a known role in proliferation, as an additional target of NCA. In accordance with the NCA phenotype, HeLa cells with a BST-2 knockout were less susceptible to inhibition of proliferation upon NCA treatment. Unexpectedly, BST-2 is degraded by the lysosome upon NCA treatment, and the release of epidermal growth factor receptor (EGFR) from lipid rafts is inhibited, with detrimental effects on cell proliferation. These results highlight an unconventional, second MoA of this natural product, which could be inspirational for novel therapeutic strategies.

## Results and Discussion

### Design and Synthesis of a Stereospecific NCA Probe

A limitation of the first-generation NCA probe NC-1 was its minimal antiproliferative activity, highlighting the need for an improved design of the alkyne attachment. Most likely, the lack of the methyl residue and the associated stereocenter was responsible for the impaired activity. We thus devised a novel synthetic approach to an NCA probe which only bears minimal perturbations by direct attachment of the alkyne moiety to the carbon skeleton of NCA and retaining the crucial *S*-configured methyl group (Figure 1A).

We synthesized NC-4 by starting from propionic acid. The acid was coupled to an oxazolidinone based chiral auxiliary (Figure 1C). The stereocenter was introduced by deprotonation in the alpha-position and the auxiliary directed addition of propargyl bromide. This yielded the substituted alkyne (**2**). Removal of the auxiliary under reductive conditions and subsequent oxidation yielded aldehyde (**4**). In a *Horner–Wadsworth–Emmons* reaction, the aldehyde was coupled to ethyl (E)-4-(diethoxyphosphoryl)but-2-enoate, resulting in ester (**5**). Reduction of the ester and reoxidation of the resulting alcohol gave an aldehyde (**6**). This aldehyde was converted in a *Wittig* reaction to a ketone (**7**). The trichloromethyl keto group was introduced by deprotonation in the alpha-position, followed by an attack of the enolate on trichloroacetic anhydride, to yield the final probe (NC-4).

NC-4 was evaluated next for its biological activities. Satisfyingly, strong antiproliferative (IC_50_ 2.17 μM) and antimigratory (IC_50_ 6.97 μM) activity were determined, emphasizing that the new probe is capable of addressing both targets (Figures 1D,S1).

### Probe Labeling in Cancer Cells Reveals BST-2 as a Novel NCA Target

**Figure 2:**
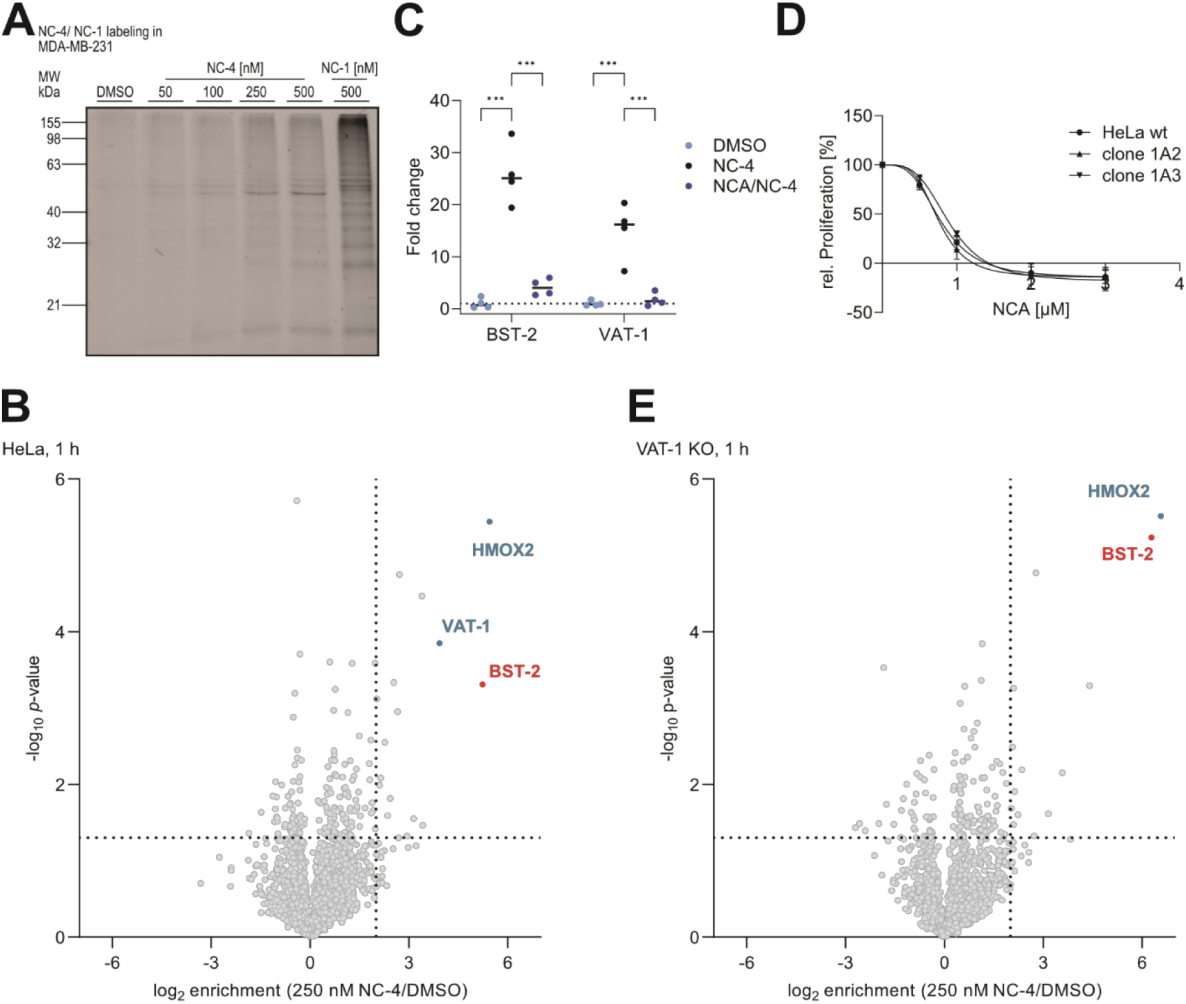
Identification of BST-2 as a cellular target of neocarzilin A. (A) SDS-Page analysis of MDA-MB-231 cells after *in situ* labeling with NC-4 (Coomassie-stained gel in Figure S2A). (B) Volcano plot of an LFQ-DDA ABPP experiment of HeLa cells labeled with 250 nM NC-4 for 1 h (*n* = 4). Proteins fulfilling the criteria *p*-value <0.05 and log2 fold change >2 were considered significantly enriched (Table S3). (C) Fold change of BST-2 and VAT-1 in an *in situ* competitive LFQ-DDA ABPP experiment in HeLa cells (*n* = 4) (for full MS data: Figure S2C, Table S4). Depicted are the fold changes of VAT-1 and BST-2 upon enrichment with the probe (NC-4) and after saturation of binding sites with NCA, followed by enrichment with NC-4 (NCA (25 μM)/NC-4 (250 nM)) in comparison to the DMSO control (DMSO). Two-way ANOVA, Dunnett’s test, ***p < 0.001. (D) Comparison of the proliferation of HeLa wt and VAT-1 KO clones in response to NCA treatment. Cells were stimulated at the indicated concentrations for 72 h, and proliferation determined by a crystal violet staining assay. Data are presented as mean ± SEM (*n* = 3). (E) Volcano plot of the in situ LFQ-DDA ABPP experiment in VAT-1 KO cells labeled with 250 nM NC-4 for 1 h (*n* = 4). Proteins fulfilling the criteria *p* value <0.05 and log2 fold change >2 were considered significantly enriched (Table S5).

NC-4 was first tested for *in situ* labeling of target proteins in the breast cancer cell line MDA-MB-231, used in our previous study, with probe concentrations ranging from 50 to 500 nM. After 1 h of incubation, cells were lysed, clicked to rhodamine azide, and the labeled proteome separated by SDS-PAGE followed by in-gel fluorescent scanning (Figure 2A).

In comparison to the first probe generation (NC-1), the superior signal-to-noise, the high band intensities, and the diverging labeling pattern indicate sufficient reactivity of the probe and coverage of so far undeciphered NCA targets. From these data, we selected 250 nM as the concentration with an optimal labeling intensity and commenced with the quantitative MS analysis.

For this, probe labeling was performed in two representative cancer cell lines, HeLa and MDA-MB-231 cells, followed by cell lysis, click ligation to biotin azide, and enrichment of probe-bound proteins on avidin beads (Figure 1B). Tryptic digestion, LC-MS/MS analysis via label-free quantification, and data-independent acquisition (LFQ-DDA) resulted in the significant enrichment of 12 proteins in MDA and 17 in HeLa cells (*p* value < 0.05, log2 fold change > 2) (Figures 2B, S2B, Tables S2, S3). Among these proteins, the previously identified hit VAT-1 was most prominently enriched, confirming the validity of the new probe NC-4. Importantly, several additional putative targets, foremost the antiviral defense protein BST-2, were among the most significant hits. In addition, heme oxygenase 2 (HMOX2), a frequent hit for covalent probes due to its reactive cysteine residues and previous target of NC-1, was also among these top targets. Target engagement of VAT-1, HMOX2, and BST-2 was further verified by competition with an excess of NCA, as visualized in the corresponding profile plots (Figures 2C and S2C, Table S4). Of note, comparing NC-4 results in MDA-MB-231 with the former NC-1 probe, lacking antiproliferative effects, BST-2 remained as the most prominent difference.^6^

To further focus on novel targets beyond VAT-1, we envisioned performing probe labeling in cells lacking VAT-1. For this, we created HeLa knockout (KO) cells by the Crispr-Cas9 technology.^12^ The lack of VAT-1 was confirmed by Western blot and whole proteome LC-MS/MS analysis (Figure S3). As expected, the antiproliferative effect of NCA was comparable between HeLa wt cells and KO clones (Figure 2D). For target analysis, the KO cells were treated with the probe as described above, and LC-MS/MS analysis confirmed HMOX2 and BST-2 as the most significant hits (Figure 2E, Table S5). BST-2 (also called tetherin, CD317, and HM1.24) is a membrane-anchored, cell-surface glycoprotein, which plays an important role in the antiviral defense.^13,14^ Moreover, BST-2 is aberrantly expressed in many cancers, and silencing studies have shown its role in cell proliferation.^15−18^ More specifically, BST-2 releases EGFR from lipid rafts and thereby activates downstream pathways.^19,20^ Thus, BST-2 was selected as a prime candidate for further studies.

### BST-2 Mediates the Antiproliferative Effects of NCA

**Figure 3:**
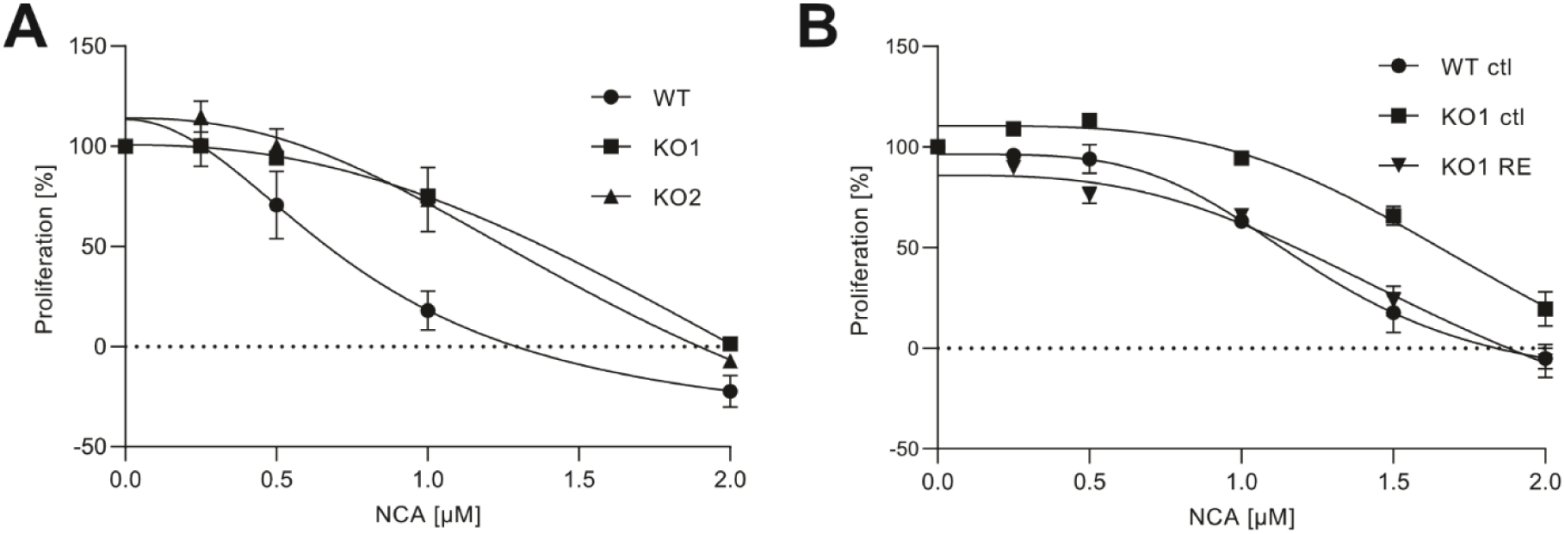
BST-2 is the antiproliferative target of NCA. (A) Antiproliferative effects of NCA in wt and BST-2-KO HeLa cells were measured by a crystal violet staining assay. Cells were treated with indicated concentrations of NCA for 72 h, and data are presented as means ± SEM (*n* = 3). (B) Antiproliferative effects of NCA in wt with empty plasmid (ctl) HeLa cells, BST-2-KO with empty plasmid (ctl) HeLa cells, and BST-2-KO HeLa cells with BST-2 reconstitution (RE). Cells were treated with the indicated concentrations of NCA for 72 h and data are presented as means ± SEM (*n* = 3).

To directly investigate the cellular effects of NCA on BST-2, we created corresponding KO strains in HeLa cells via Crispr-Cas9, as confirmed by Western blot and whole-proteome LC-MS/MS analysis (Figure S4). Interestingly, the BST-2-KO HeLa cells were less sensitive to the antiproliferative effect of NCA (Figure 3A).

Vice versa, reconstitution of BST-2 in KO cells via overexpression using a respective plasmid reinduced the sensitivity of the cells toward NCA to approximately the same level as in wild-type cells (Figure 3B). This confirms BST-2, a major antiproliferative target of NCA.

### NCA Treatment Reduces BST-2 Levels via Lysosomal Degradation

**Figure 4:**
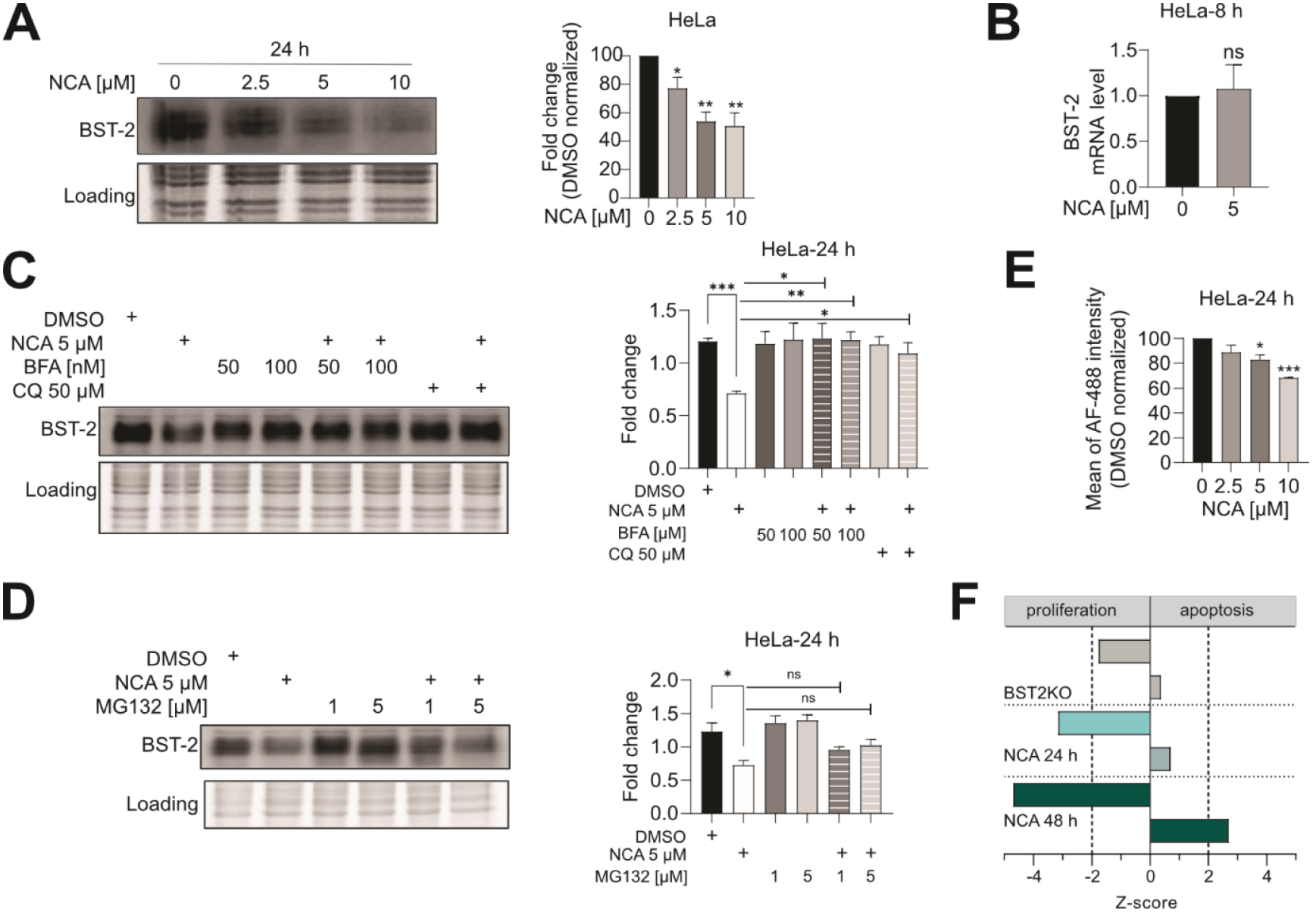
NCA promotes BST-2 protein degradation via the lysosomal pathway in a concentration-dependent manner. (A) Western blot analysis of the BST-2 protein level in HeLa cells treated with different concentrations of NCA for 24 h. Representative blots of three independent experiments are shown (all replicates in Figure S6). The amount of BST-2 was normalized to the loading control, and the results were normalized to the DMSO control. Data are presented as means ± SEM (*n* = 3), one-way ANOVA, Dunnett’s test, **p < 0.002. (B) qPCR analysis of BST-2 mRNA level in DMSO- or NCA-treated HeLa cells for 8 h. Data are presented as means ± SEM (*n* = 3), unpaired *t*-test with Welch’s correction, ^ns^*p* > 0.12. (C) Western blot analysis of BST-2 protein level in HeLa cells with indicated treatment. HeLa cells were pretreated with BFA or CQ for 1 h before NCA treatment for 24 h. Representative blots of three independent experiments are shown (all replicates in Figure S7–9). The amount of BST-2 was normalized to loading control and data are presented as means ± SEM (*n* = 3), one-way ANOVA, Dunnett’s test, *p < 0.033. (D) Western blot analysis of BST-2 protein level in HeLa cells with indicated treatment. HeLa cells were pretreated with MG132 for 1 h before NCA treatment for 24 h. Representative blots of three independent experiments are shown (all replicates in Figure S7–9). The amount of BST-2 was normalized to loading control, and the data are presented as means ± SEM (*n* = 3), one-way ANOVA, Dunnett’s test, ^ns^*p* > 0.12, **p < 0.002. The diffuse bands of BST-2 in the Western blots are due to different glycosylation patterns of BST-2. (E) BST-2 surface-level analysis of HeLa cells with indicated concentrations of NCA for 24 h (Figure S10). Data are presented as means ± SEM (*n* = 3), one-way ANOVA, Dunnett’s test, *p < 0.033, ***p < 0.001. (F) Ingenuity pathway analysis (disease and function) of whole proteome data of BST-2-KO, 2.5 μM NCA 24 h-treated, and 2.5 μM NCA 48 h-treated HeLa cells compared to the respective controls. Significance threshold |*z*-score| ≥ 2.

To further analyze the cellular effects of NCA treatment on the global proteome of HeLa cells and, in particular, BST-2, we monitored protein expression levels via LC-MS/MS whole proteome analysis. NCA treatment does not significantly affect HMOX2 levels, indicating that the enrichment of HMOX2 in ABPP experiments (Figure 2B,E) is due to an interaction of the probe and HMOX2 and not a result of an upregulation of HMOX2 as part of the cellular xenobiotic detoxification program. Interestingly, BST-2, on the other hand, was significantly reduced in abundance in NCA-treated cells after 24 h (Figure S5). This intriguing discovery raises a question about the underlying mechanism of diminished BST-2 levels. First, we confirmed the reduction of BST-2 levels via Western blot within whole HeLa cells upon NCA treatment in a concentration-dependent manner (Figures 4A, and S6). Interestingly, the change in abundance of BST-2 must occur posttranscriptionally as the mRNA levels remained constant (Figure 4B).

Consequently, we focused next on mechanisms of protein degradation to explain the loss of BST-2 after treatment with NCA. To narrow down possible degradation pathways, we added the proteasome inhibitor MG132, or the autophagosome-lysosome inhibitors bafilomycin A (BFA) and chloroquine (CQ) and observed a stalled BST-2 degradation in the case of bafilomycin A and chloroquine, indicative of lysosomal degradation (Figures 4C, D, and S7–9). Interestingly, lysosomal degradation of BST-2 has been reported as a viral entry strategy by which the viral protein Vpu induces ubiquitinylation of BST-2, followed by lysosomal removal and subsequent viral entry.^21,22^ To investigate whether degradation of BST-2 correlates with its abundance in the membrane, we measured surface levels by flow cytometry after antibody staining. Surface levels of BST-2 were reduced to a similar degree as total BST-2 levels (Figures 4E, and S10). We next rationalized the consequences of NCA treatment and the reduced BST-2 levels on the HeLa cell proteome by MS analysis. Ingenuity pathway analysis of whole proteome data of BST-2-KO HeLa cells in comparison to NCA-treated cells (for 24 and 48 h) revealed an influence on processes connected to protein proliferation and apoptosis with NCA treatment over 48 h having the strongest effect (Figure 4F).

To test if BST-2 degradation upon NCA treatment is mediated by ubiquitinylation, we overexpressed BST-2 fused to a HA-tag in HeLa cells and treated the cells with NCA (5 μM) for 2, 4, 6, or 24 h. We lysed the cells and immunoprecipitated BST-2 via the HA-tag. Western blot analysis showed no significant change in ubiquitinylation upon NCA treatment. This indicates that BST-2 degradation upon NCA treatment is not mediated by ubiquitin (Figure S11).

BST-2 was not degraded upon treatment with the weak antiproliferative compound NC-1 (Figure S12). However, this residual activity suggests that other targets are involved in the antiproliferative effect.

### NCA Impairs EGFR Release from Lipid Rafts

**Figure 5:**
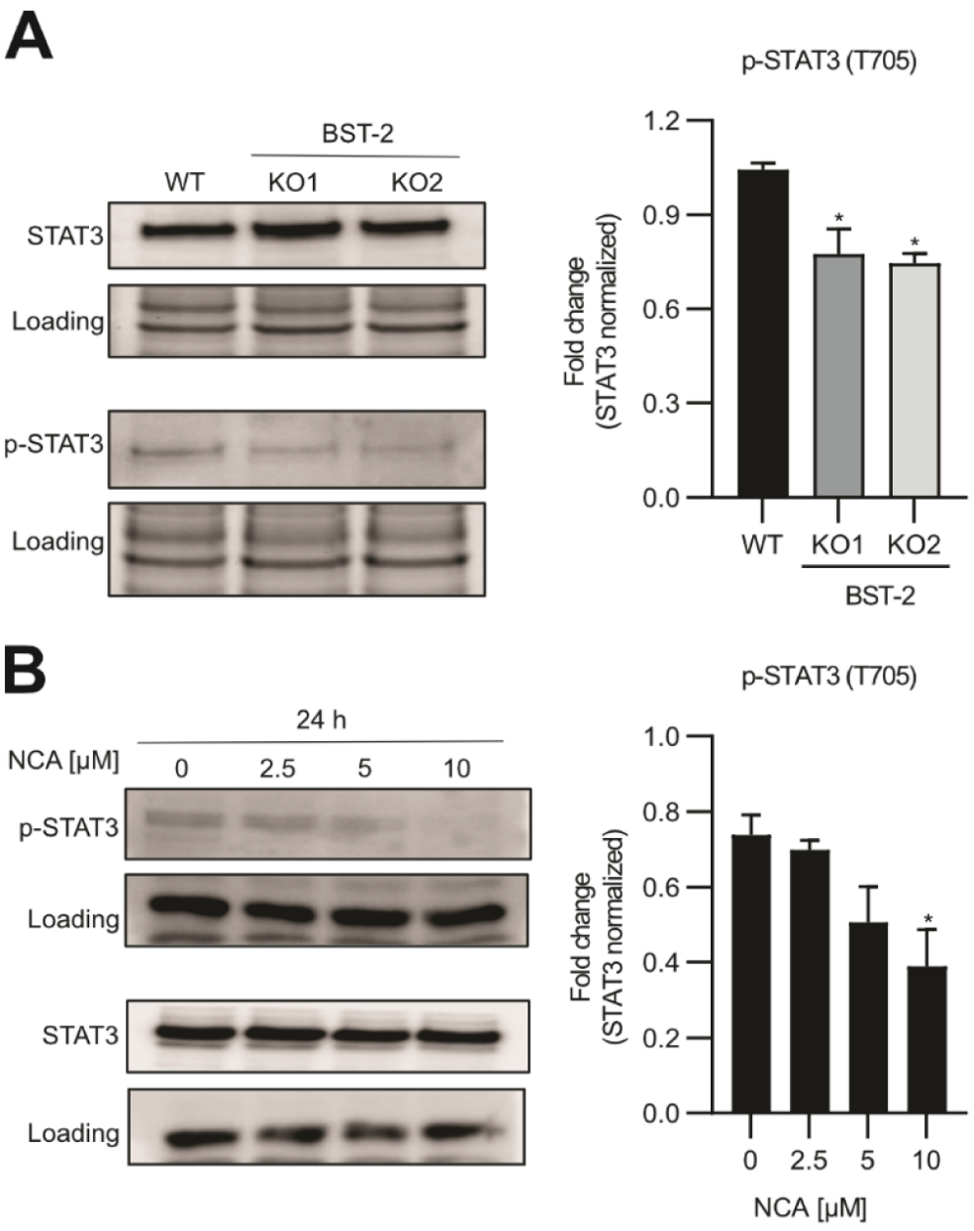
BST-2-KO and NCA influence the EGFR-STAT signaling pathway. (A) Left: Western blot analysis of STAT3 and p-STAT3 protein levels in wt and BST-2-KO cells. Representative blots of three independent experiments are shown. Right: Bar graphs show the mean ± SEM of three independent experiments, and the amount of p-STAT3 was normalized to STAT3, respectively. One-way ANOVA, Dunnett’s test, *p < 0.033, **p < 0.002. (B) Left: Western blot analysis of STAT3 and p-STAT3 protein levels in HeLa cells with indicated concentrations of NCA treatment for 24 h. Representative blots of three independent experiments are shown. Right: Bar graphs show the mean ± SEM of three independent experiments and the amount of p-STAT3 was normalized to STAT3, respectively. One-way ANOVA, Dunnett’s test, *p < 0.033, **p < 0.002. Blots for STAT3 and p-STAT3 were run separately.

Since BST-2 has been previously shown to facilitate the release of EGFR from lipid rafts,^19^ we hypothesized that this might be the mechanistic link between NCA treatment and its antiproliferative effect. EGFR is a receptor tyrosine kinase and a key regulator of cell proliferation. It is commonly upregulated or mutated in cancer cells, and, consequently, an attractive target for antiproliferative therapies.^23^ Upon ligand binding, the receptor autophosphorylates on multiple sites, and main downstream signaling comprises the AKT-PI3K-mTOR, the RAS-RAF-MEK-ERK MAPK, and the STAT pathways.^23,24^ While MS-based phosphoproteome analysis of HeLa cells treated with 10 μM NCA for 6 h did not reveal EGFR phosphotyrosines, increased phosphorylation of T693, a modification known to be associated with reduced EGFR activity and corresponding attenuated growth, was observed (Figure S13).^25^ Accordingly, we analyzed the phosphorylation status of the downstream kinase STAT3 of NCA-treated HeLa cells and BST-2-KO cells via Western blot. We observed significantly reduced STAT3 (Y705) phosphorylation both in BST-2-KO and after treatment with NCA, confirming an inhibition of the corresponding pathways (Figure 5A, B). Of note, analysis of STAT3 phosphorylation upon NCA treatment in BST-KO cells also showed a concentration dependent but less-pronounced decrease in phosphorylation of STAT3 compared to wt HeLa cells. These results further suggest that, in addition to BST-2, other pathways may contribute to the antiproliferative effect of NCA.

**Figure 6:**
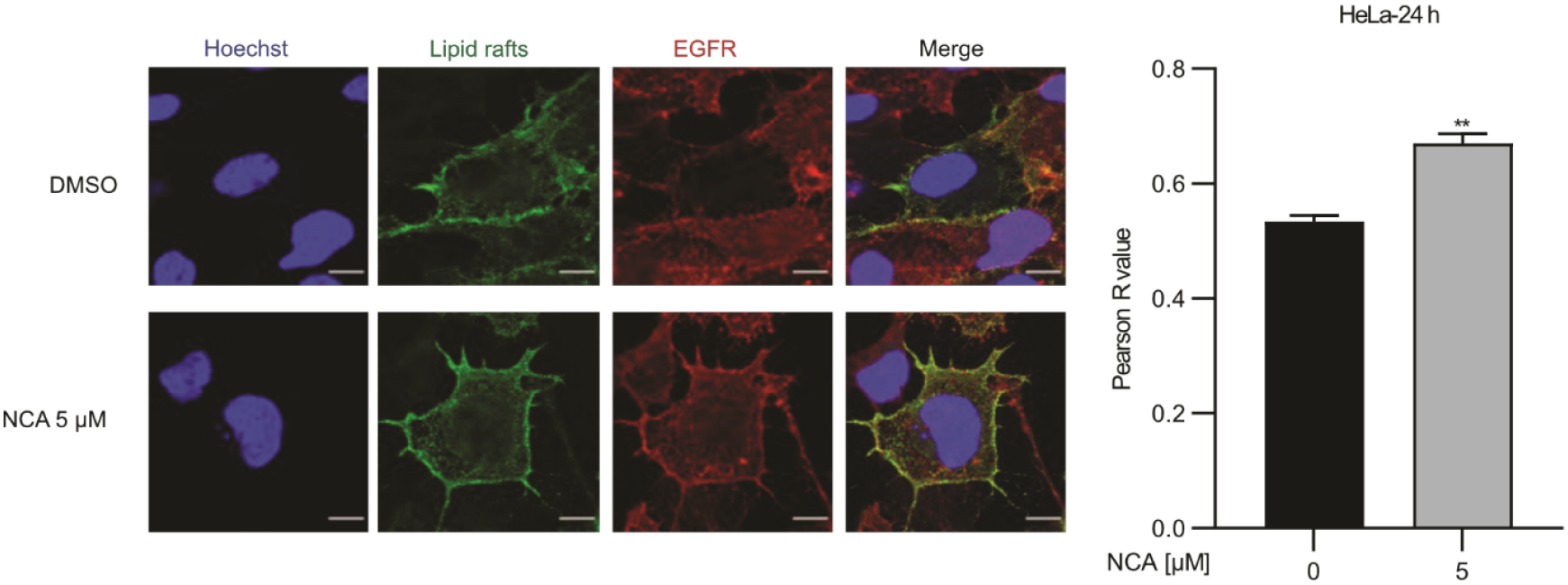
NCA affects the dynamics of the EGFR protein by sequestering EGFR in lipid rafts. Left: HeLa cells treated with DMSO or NCA were immunostained for EGFR (red), lipid rafts (green), and nuclei (blue). Shown are representative fluorescence images. Scale bar: 10 μm. EGFR-lipid rafts colocalization plot and Pearson’s correlation coefficient (R) (right; mean ± SEM for at least 60 cells). Unpaired *t*-test with Welch’s correction, **p < 0.01. Binding of cholera toxin subunit B (CT-B) is used as a marker for lipid rafts.

To investigate the cellular localization of EGFR and lipid rafts, costaining was performed using confocal microscopy. Upon treatment with NCA, the colocalization of a lipid raft marker and EGFR staining was compared to solvent controls and revealed an increased Pearson’s correlation coefficient indicative of an enhanced interaction (Figures 6, and S14). Intriguingly, these results provide further evidence on the impaired function of BST-2 under NCA treatment, resulting in EGFR stalled in lipid rafts and inhibition of downstream signaling.

## Conclusion

Natural products are known to often address more than one target in order to maximize their biological effects. NCA follows this logic and acts on cancer cells via inhibiting migration as well as proliferation. As this dual mode of action is quite unique, deciphering the molecular targets not only enlightens the fundamental principles of nature’s mechanisms of action but also facilitates intriguing perspectives for the design of novel therapeutics against druggable targets. The discovery of BST-2 as an additional target of NCA provides unprecedented insight into a unique MoA by which compound binding induces lysosomal protein degradation. Our experiments further showed that other targets could contribute to the overall antiproliferative effects of NCA. Together with our previous studies on the inhibition of migration via binding to VAT-1, we conclude that NCA impairs cell physiology at two crucial pathways, explaining its high anticancer potency.

Elucidation of how NCA binding drives BST-2 into the lysosomal degradation pathway and whether this could be used for targeted approaches will be subject to future studies. Of note, degradation of proteins by natural products acting as molecular glues for proteasomal degradation has been recently discovered, adding NCA, to the best of our knowledge, as the first example of the lysosomal pathway.^4,26^

Although we were unable to decipher the NCA binding site on BST-2 via MS, probably due to the transient nature of the electrophilic warhead, we demonstrate that antiproliferative effects are mediated via BST-2 in corresponding KO and overexpression cell lines. Furthermore, reduced levels of BST-2 upon lysosomal degradation prevent the release of EGFR from lipid rafts, which immediately affects downstream signaling and proliferation. Overall, BST-2 represents a promising hot spot for therapeutic approaches, as it drives the activity of the crucial downstream EGFR pathway. Pharmacologically fine-tuned NCA derivatives switching on the degradation of aberrantly expressed BST-2 in cancer cells represent a novel and effective treatment option.

## Supporting Information Available

Supplementary figures S1–14, supplementary tables S1–5, supplementary experimental details, synthetic procedures and compound characterization (HRMS, ^1^H NMR, ^13^C NMR) data (PDF)

## Abbreviations

ABPP: activity-based protein profiling
BFA: Bafilomycin A
BST-2: bone marrow stromal antigen 2
CQ: chloroquine
CRISPR: clustered regularly interspaced short palindromic repeats
DDA: data-dependent acquisition
EGFR: epidermal growth factor receptor
HMOX2: heme oxygenase 2
LFQ: label-free quantification
MoA: mode of action
NCA: neocarzilin A
VAT-1: vesicle amine transport protein 1
wt: wild-type
