# Supplementary material for: Neocarzilin Inhibits Cancer Cell Proliferation via BST-2 Degradation, Resulting in Lipid Raft-Trapped EGFR
Source: JACS Au. 2024 May 8;4(5):1833–40. doi: 10.1021/jacsau.4c00039 (PMC11134574; doi:10.1021/jacsau.4c00039)
Supplement: Supplementary file 1 — au4c00039_si_001.pdf [file au4c00039_si_001.pdf]

Neocarzilin inhibits cancer cell proliferation via BST-2 degradation resulting in lipid raft trapped EGFR

Josef Braun[†,#], Yudong Hu[‡,#], Adrian T. Jauch[‡#], Thomas F. Gronauer[†,§], Julia Mergner°, Nina C. Bach[†], Franziska R. Traube[⊥], Stefan Zahler*[,‡], Stephan A. Sieber*[,†]

[†]TUM School of Natural Sciences, Department of Bioscience, Chair of Organic Chemistry II, Center for Functional Protein Assemblies (CPA), Technical University of Munich (TUM), Ernst-Otto-Fischer Straße 8, Garching near Munich, D-85748, Germany

[‡]Department of Pharmacy, Pharmaceutical Biology, Ludwig-Maximilians-University in Munich (LMU), Butenandtstraße 5-13, Munich, D-81377, Germany

°Bavarian Center for Biomolecular Mass Spectrometry at Klinikum rechts der Isar (BayBioMS@MRI), Technical University of Munich (TUM), Einsteinstraße 25, Munich, D-81675, Germany

[⊥]Institute of Biochemistry and Technical Biochemistry, University of Stuttgart, Allmandring 31, Stuttgart, D-70569, Germany

§Metabolomics and Proteomics Core (MPC), Helmholtz Zentrum München GmbH German Research Center for Environmental Health, Heidemannstr. 1, D-80939, Munich, Germany

# These authors contributed equally to this work.

-Supporting Information –

Table of contents

1. Supplementary Figures S1-S14 3
2. Methods 15
   2.1 Cell culture and cell lines 15
   2.2 MS-based proteomic procedures 15
      2.2.1 *In situ* labeling in human cells 15
      2.2.2 Analytical *in situ* labeling 15
      2.2.3 *In situ* preparative labeling label-free quantification 15
      2.2.4 Whole proteome analysis 16
      2.2.5 MS/MS measurement Orbitrap Fusion 16
      2.2.6 Bioinformatics and statistics 17
      2.2.7 Ingenuity pathway analysis (IPA) 17
   2.3 Phosphoproteomic procedures 17
      2.3.1 Sample Preparation 17
      2.3.2 Mass spectrometric measurement 18
      2.3.3 Bioinformatics and statistics 19
   2.4 Cell based assays 19
      2.4.1 Compounds and treatment 19
      2.4.2 Crystal violet staining assay 19
      2.4.3 xCELLigence® assay 20
      2.4.4 Quantification and statistical analysis 20
   2.5 Immunological procedures 20
      2.5.1 Antibodies 20
      2.5.2 Detection of BST-2 surface level 20
      2.5.3 Western Blot 21
      2.5.4 Lipid rafts staining 21
      2.5.5 Immunoprecipitation 21
   2.6 Genetic Methods 22
      2.6.1 Generation of a VAT-1 knockout cell line 22
      2.6.2 Generation of a BST-2 knockout cell line 22
      2.6.3 Quantitative real-time PCR analysis 22
3 Supplementary Tables 23
4 Synthetic procedures 25
   4.4 General Methods and Materials 25
   4.5 Synthesis 26
      4.5.1 Synthesis of NCA 26
      4.5.2 Synthesis of NC-4 26
5 NMR Spectra 32

6 Safety Statement..40
7 Supplementary References 40

1. Supplementary Figures S1-S14

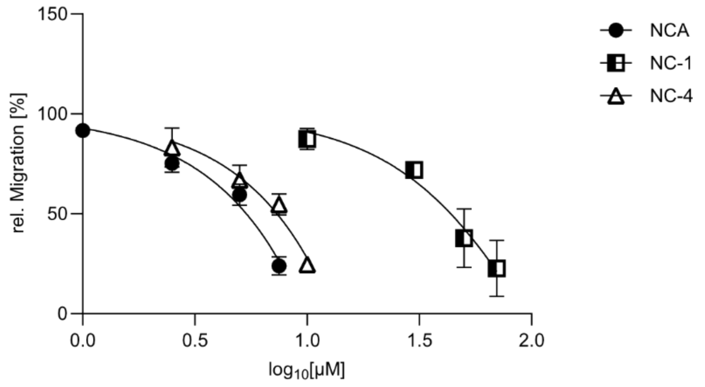

Figure S1 Antimigratory activity of NCA, NC-1 and NC-4. xCELLigence® migration assay of HeLa wt cells treated at the indicated concentrations, impedance was recorded over 18 h. Slope of the obtained cell index curves was calculated and normalized to DMSO control as relative migration [%]. Data are presented as mean ± SEM, (n = 3).

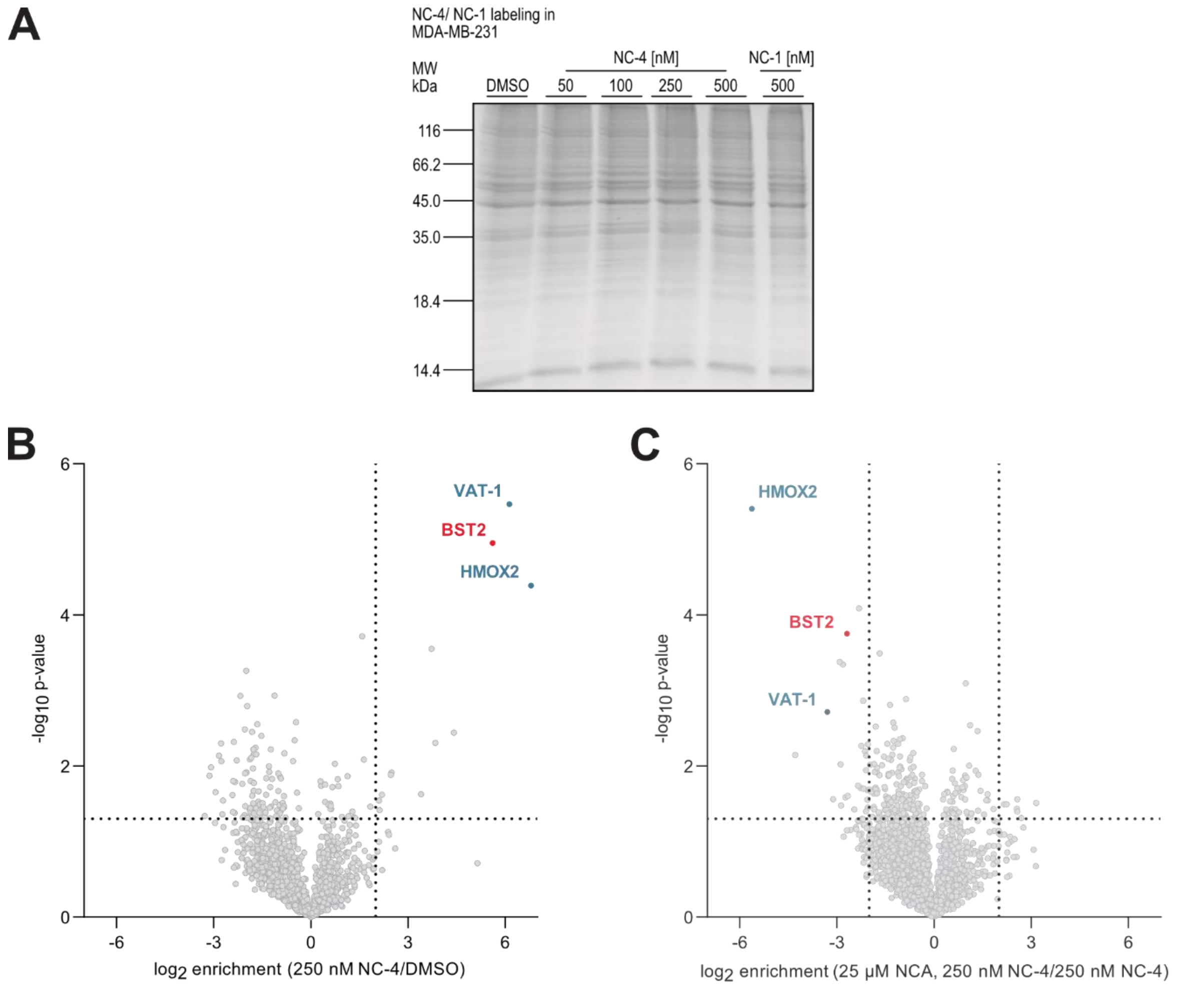

Figure S2 *In situ* labeling with NC-4. (A) Coomassie stained SDS-Page analysis of MDA-MB-231 cells after *in situ* labeling with NC-4 (Figure 2A fluorescent image of gel). (B) Volcano plot of an LFQ ABPP experiment of

MDA-MB-231 cells labeled with 250 nM NC-4 for 1 h (n = 4). Proteins fulfilling the criteria p-value < 0.05 and log2 fold-change > 2 were considered significantly enriched (Table S2). (C) Volcano plot of *in situ* competitive LFQ ABPP experiment in HeLa cells (NCA (25 µM) /NC-4 (250 nM), 1 h each) (n = 4). Proteins fulfilling the criteria p-value < 0.05 and log2 fold-change < -2 were considered significantly outcompeted (Table S4).

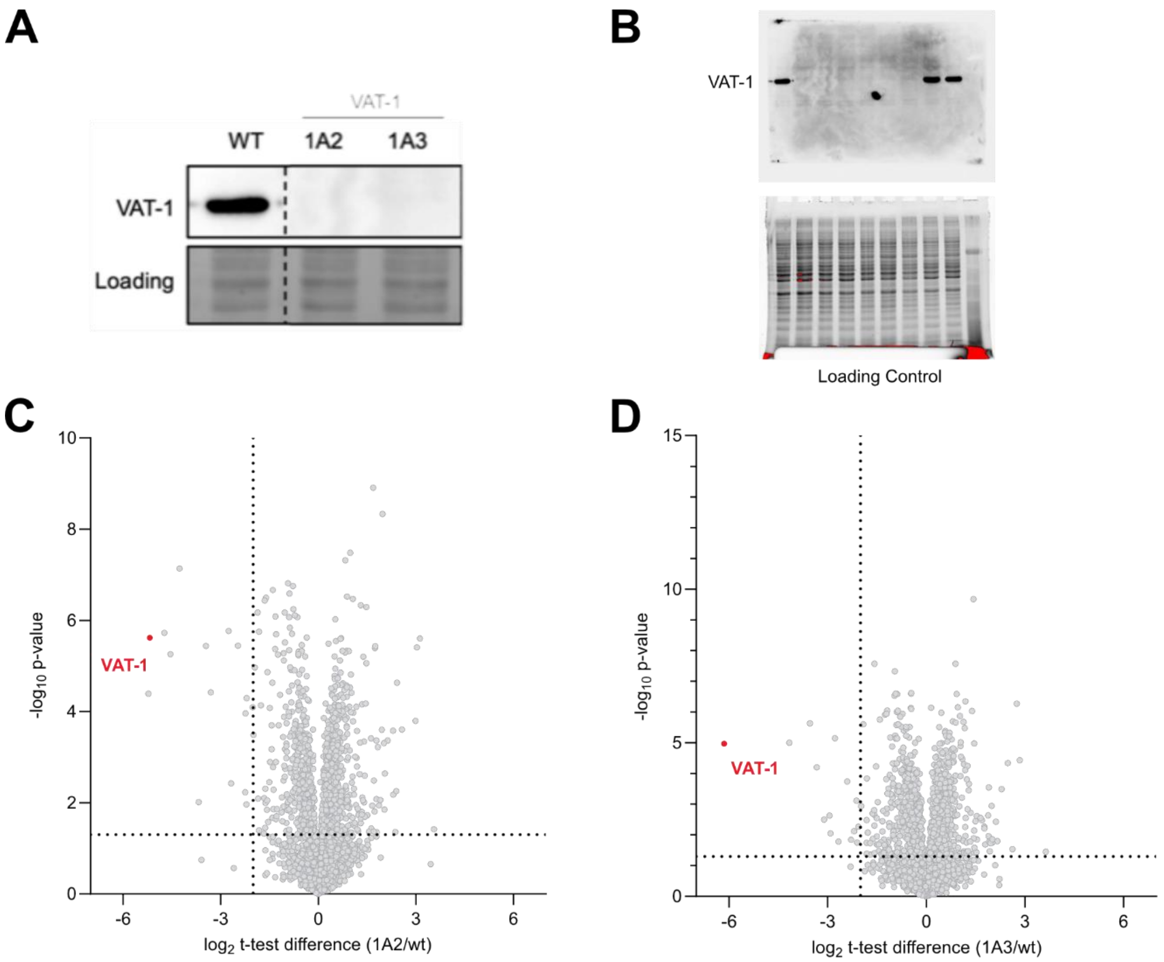

Figure S3 Validation of VAT-1 CRISPR knockout clones. (A) Western blot of VAT-1 protein levels in HeLa wt cells and HeLa VAT-1 KO clones generated via CRISPR-Cas9. (B) Uncropped version of Figure S3A. (C) Volcano plot of whole proteome analysis of KO clone 1A2 compared to HeLa wt cells (n =4). Proteins fulfilling the criteria p-value < 0.05 and log2 fold-change < -2 were considered significantly downregulated (values for VAT-1 imputed. VAT-1 was not detected by MS in KO clones). (D) Volcano plot of whole proteome analysis of KO clone 1A3 compared to HeLa wt cells (n =4). Proteins fulfilling the criteria p-value < 0.05 and log2 fold-change < -2 were considered significantly downregulated (values for VAT-1 imputed. VAT-1 was not detected by MS in KO clones).

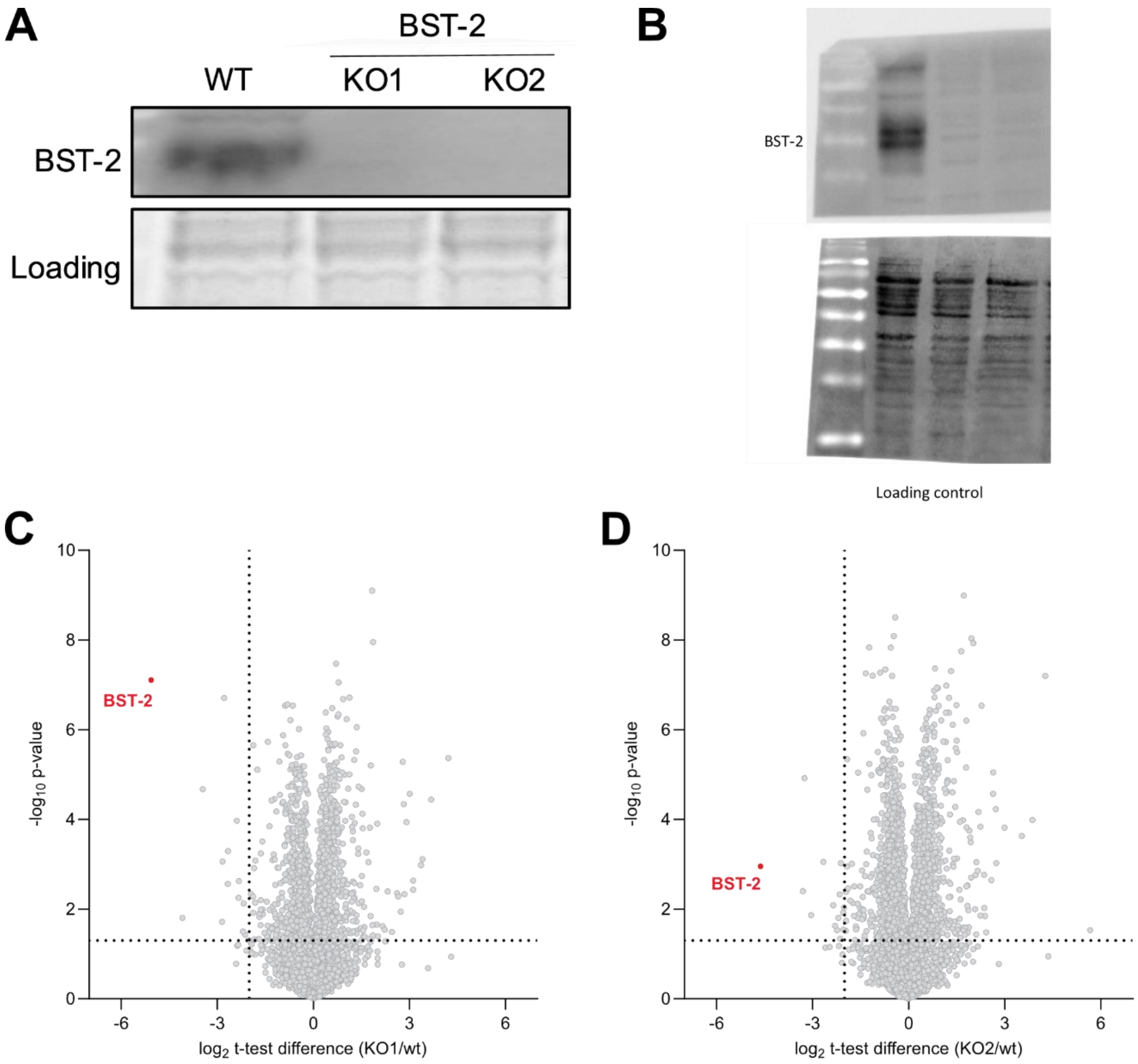

Figure S4 Validation of BST-2 CRISPR knockout clones. (A) Western blot of BST-2 protein levels in HeLa wt cells and HeLa BST-2 KO clones generated via CRISPR-Cas9. (B) Uncropped version of Figure S4A. The diffuse bands for BST-2 are due to different glycosylation patterns of BST-2. (C) Volcano plot of whole proteome analysis of KO clone KO1 compared to HeLa wt cells (n =4). Proteins fulfilling the criteria p-value < 0.05 and log2 fold-change < -2 were considered significantly downregulated (values for BST-2 imputed. BST-2 was not detected by MS in KO clones). (D) Volcano plot of whole proteome analysis of KO clone KO2 compared to HeLa wt cells (n =4). Proteins fulfilling the criteria p-value < 0.05 and log2 fold-change < -2 were considered significantly downregulated (values for BST-2 imputed. BST-2 was not detected by MS in KO clones).

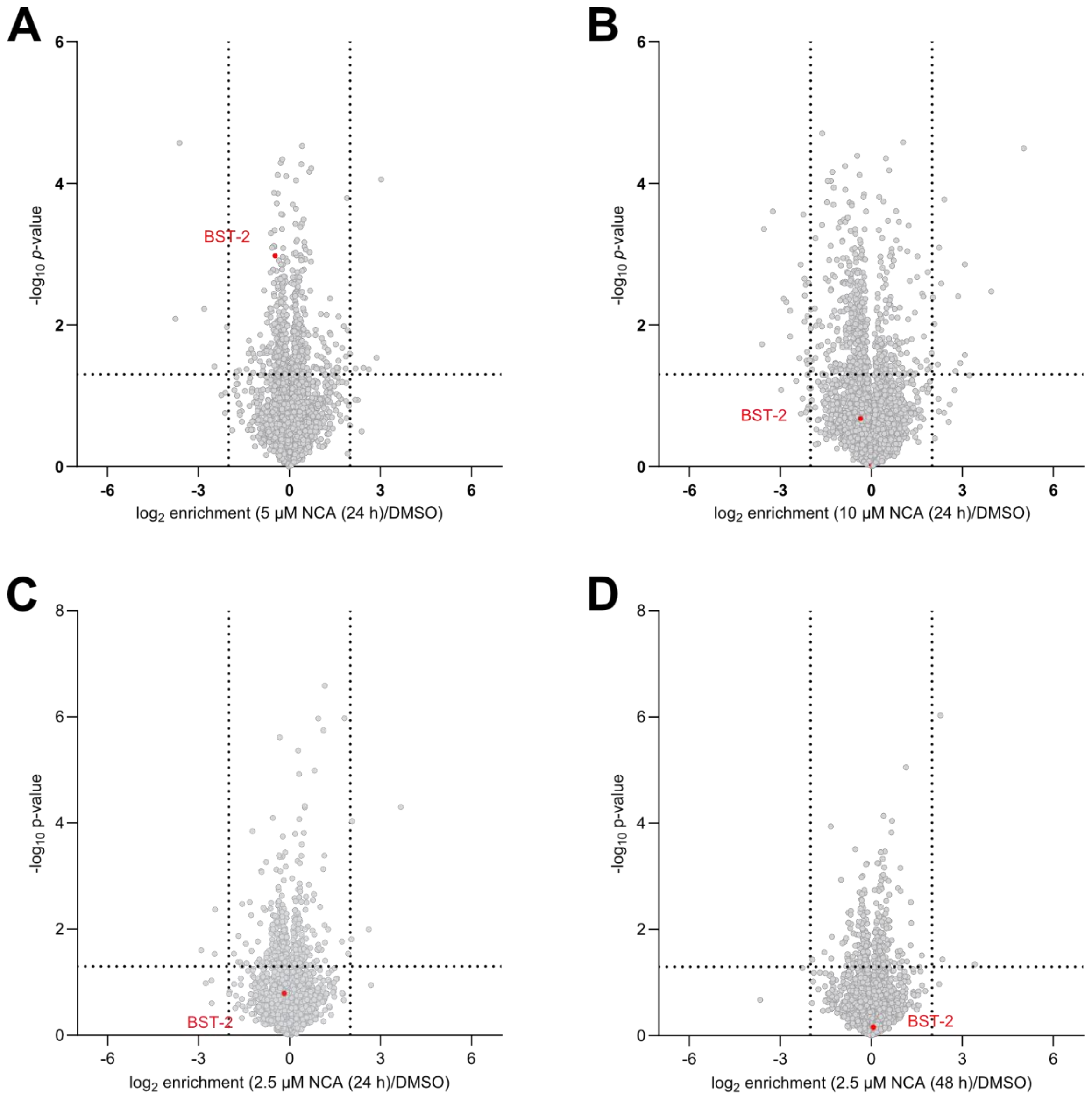

Figure S5 Cellular effects of NCA. (A) Volcano plot of whole proteome analysis of HeLa cells treated with 5 µM NCA for 24 h. Proteins fulfilling the criteria p-value < 0.05 were considered significant. (B) Volcano plot of whole proteome analysis of HeLa cells treated with10 µM NCA for 24 h. Proteins fulfilling the criteria p-value < 0.05 were considered significant. (C) Volcano plot of whole proteome analysis of HeLa cells treated with 2.5 µM NCA for 24 h. Proteins fulfilling the criteria p-value < 0.05 were considered significant. (D) Volcano plot of whole proteome analysis of HeLa cells treated with 2.5 µM NCA for 48 h. Proteins fulfilling the criteria p-value < 0.05 were considered significant.

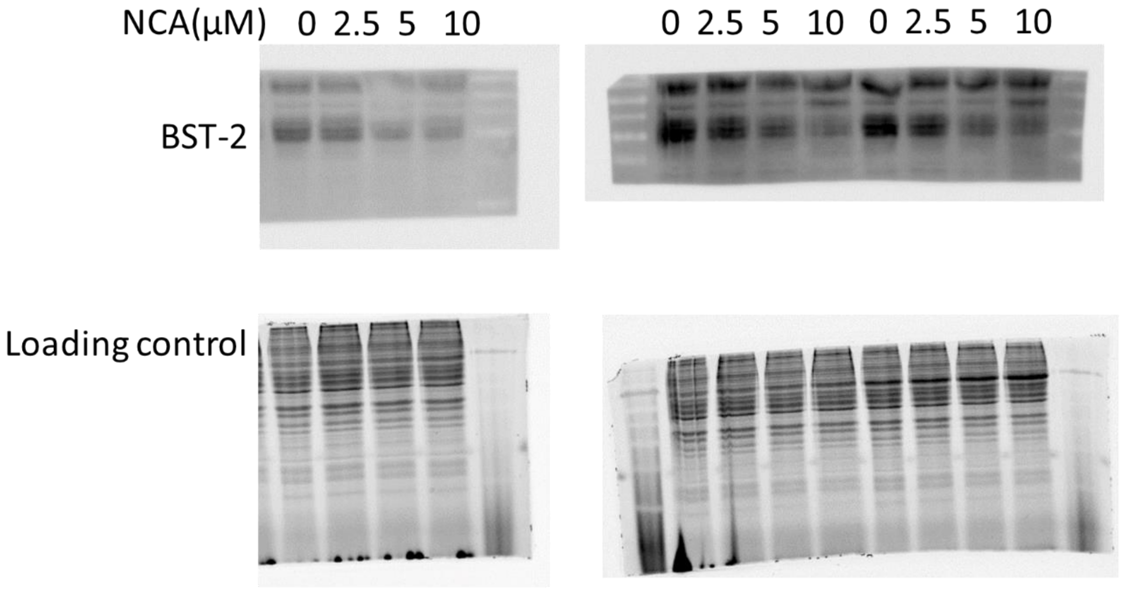

Figure S6 Replicates of the western blot analysis of BST-2 protein level in HeLa cells treated with different concentrations of NCA for 24 h (Figure 4 A). (left) replicate 1, (right) replicate 2 & 3.

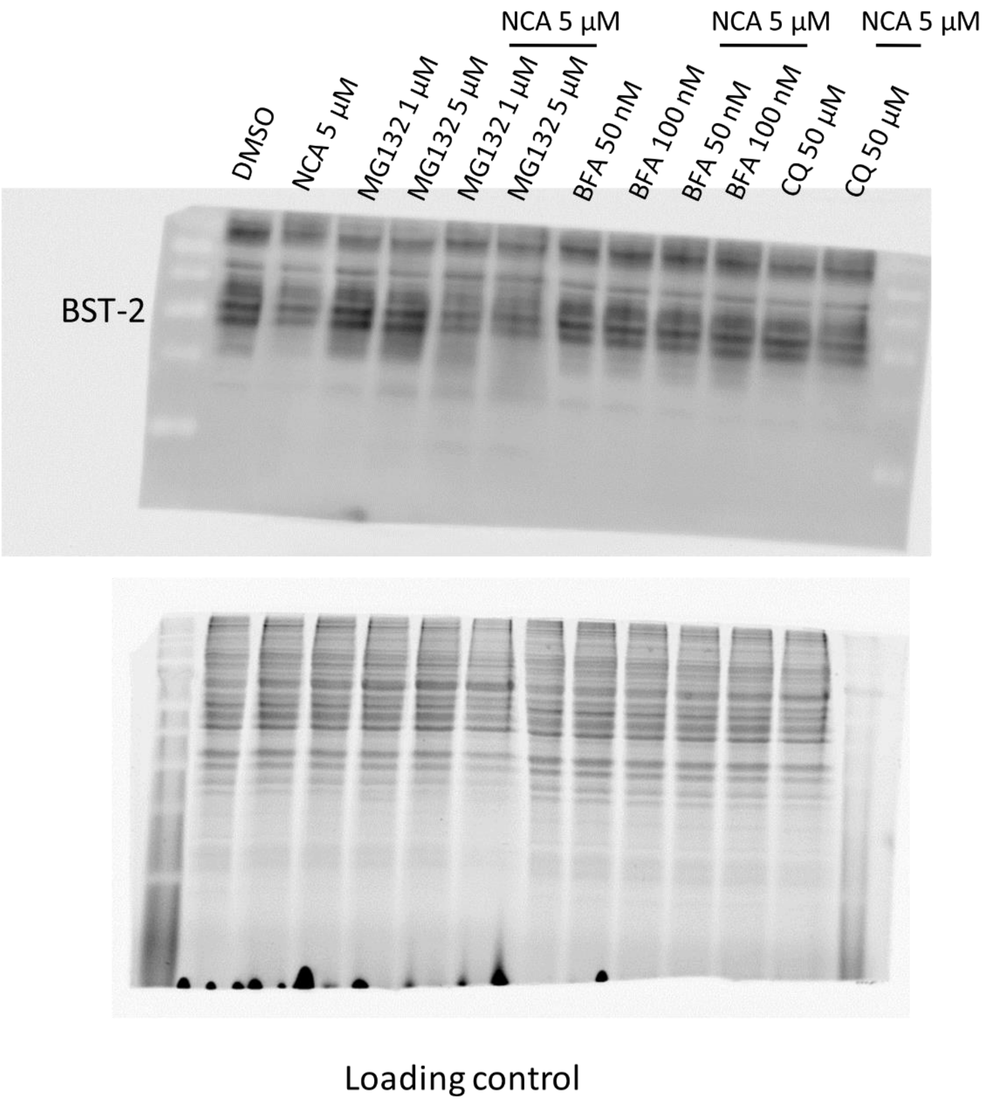

Figure S7 Western blot analysis of BST-2 protein level in HeLa cells with indicated treatment (Figure C & D). Replicate 1.

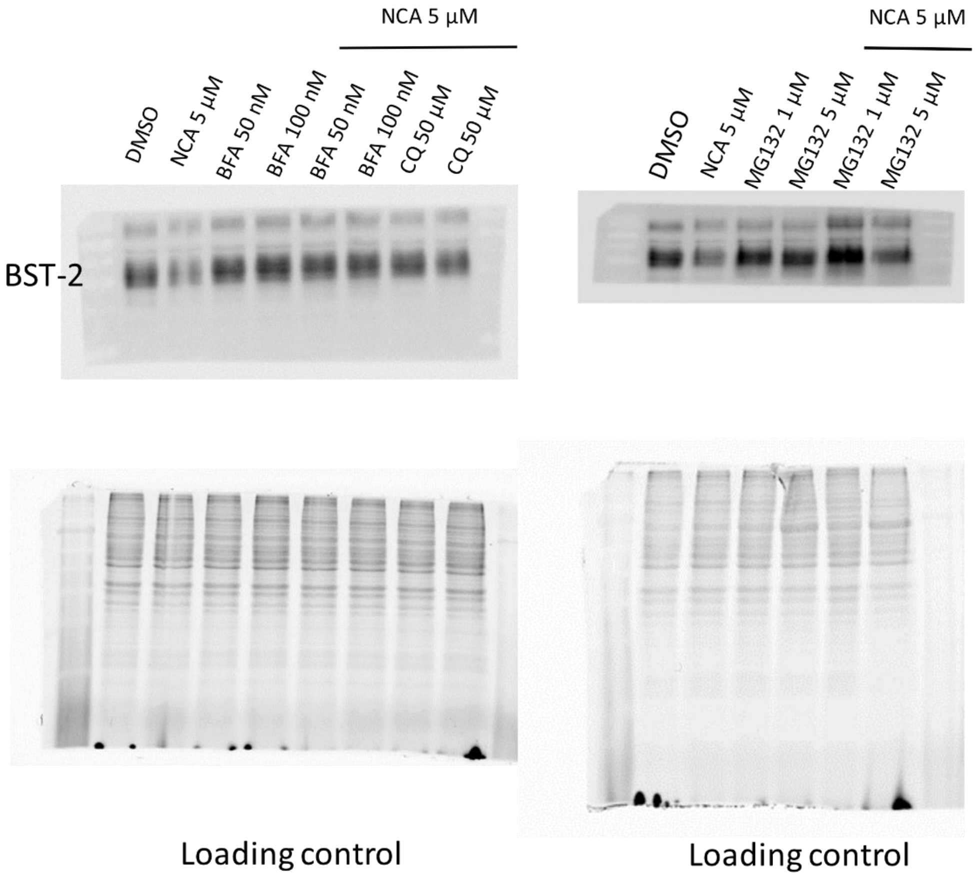

Figure S8 Western blot analysis of BST-2 protein level in HeLa cells with indicated treatment (Figure C & D). Replicate 2.

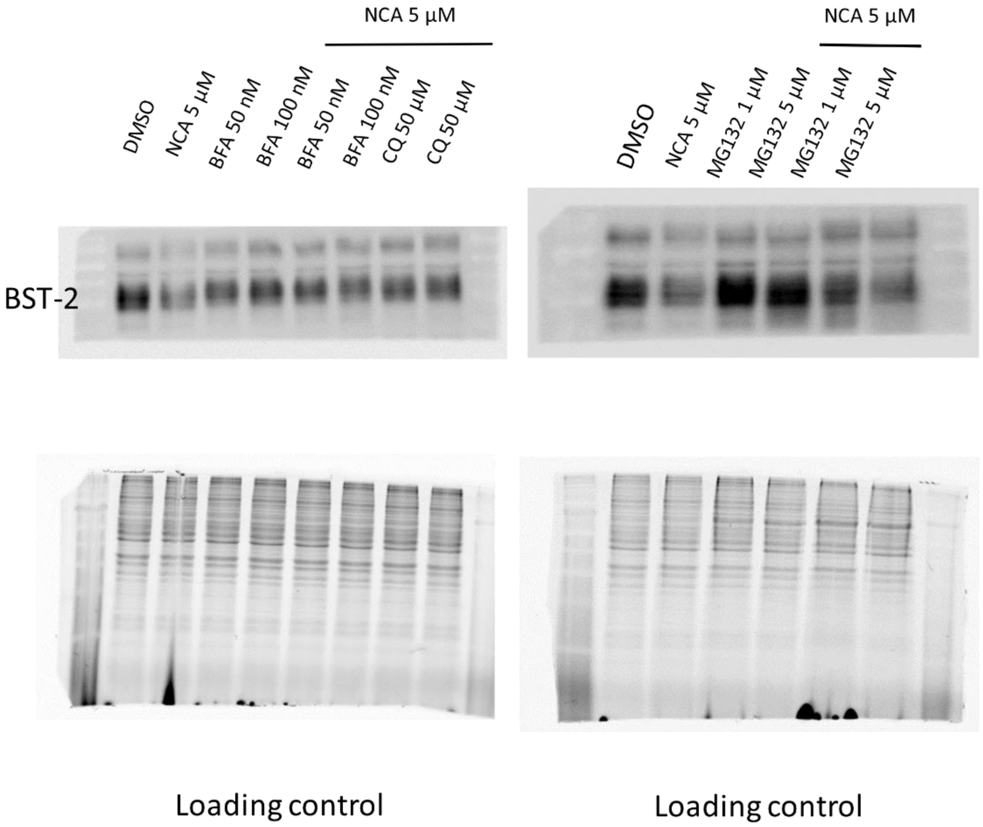

Figure S9 Western blot analysis of BST-2 protein level in HeLa cells with indicated treatment (Figure C & D). Replicate 3.

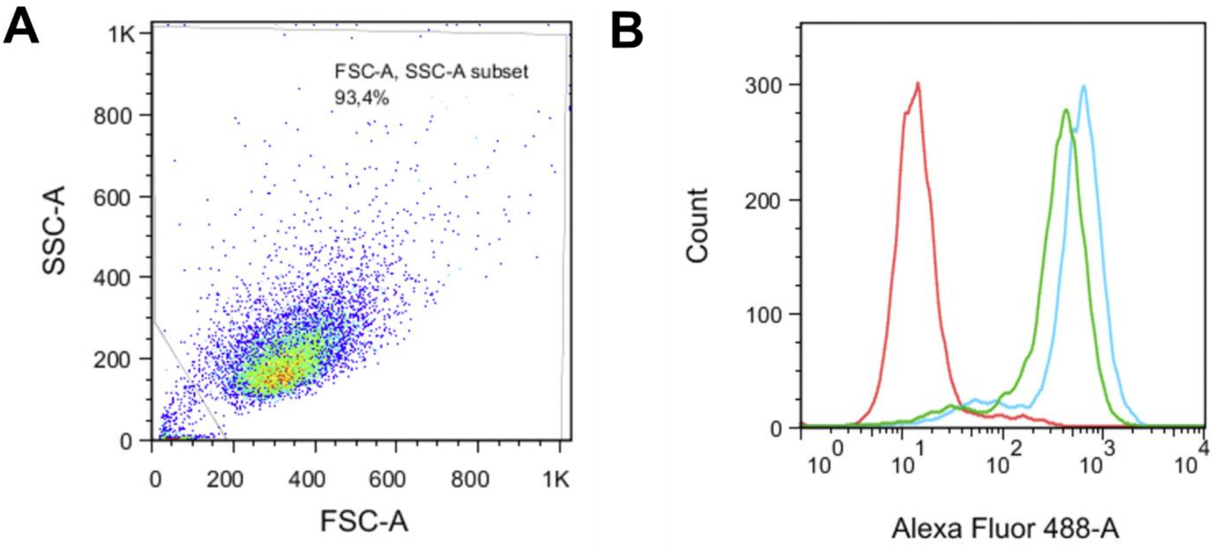

Figure S10 Quantification of BST-2 surface levels by flow cytometry. (A) Analysis of HeLa cells by flow cytometry. The dot plot shows a homogenous population of cells. The debris in the lower left were gated (light gray frame) and dismissed for analysis, (B) Histogram of the fluorescence intensity of BST-2 on the cell surface (stained with Alexa Fluor 488 coupled antibody). Red: Isotype control, blue: untreated cells, green: cells treated with NCA (10 µM, 24 h).

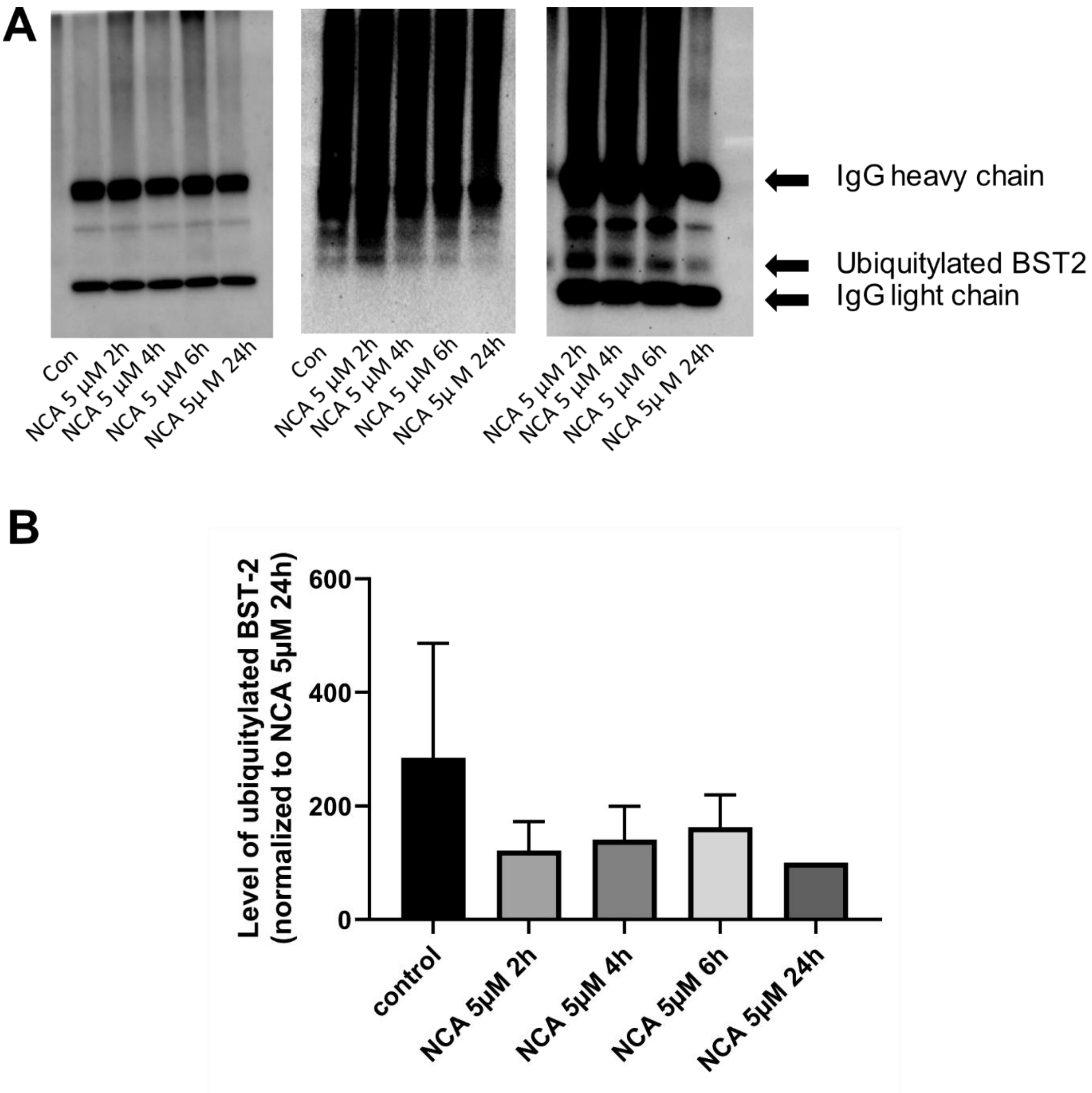

Figure S11 BST-2 degradation after NCA treatment is independent of ubiquitinylation. (A) Western blot analysis of ubiquitinylated BST-2 protein level in HeLa cells treated with 5 µM NCA for 2,4 ,6 or 24 h. Blots of three independent experiments are shown. (B) The amount of ubiquitinylated BST-2 was normalized to the loading control and results were normalized to the DMSO control. Data are presented as means ± SEM, (n = 3). For IP of BST-2, a mouse antibody against HA-tag was used, for detection of ubiquitin, a rabbit anti-ubiquitin antibody was used. One-way ANOVA and Kruskall-Wallis as *post hoc* test indicated no significant differences between groups.

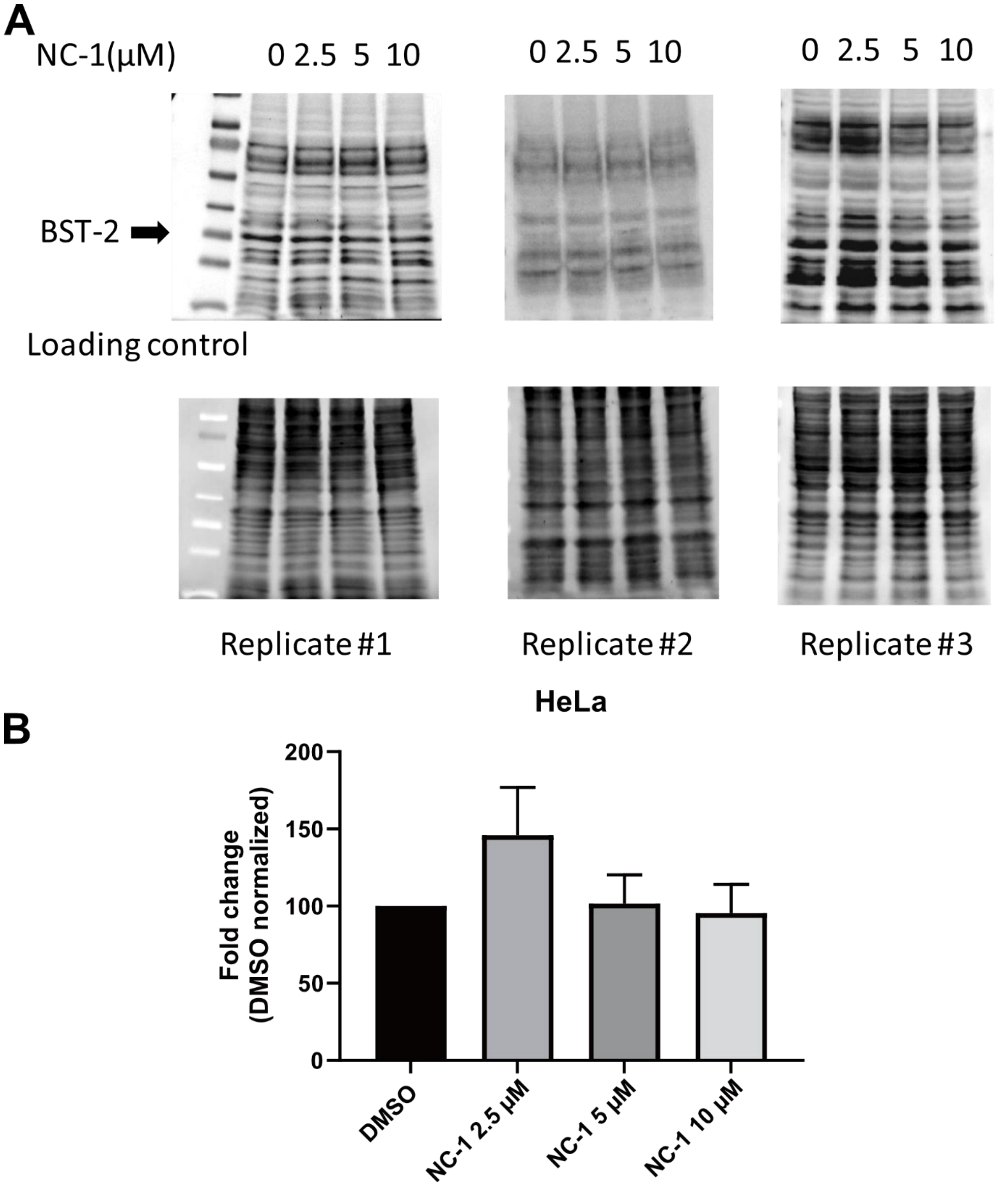

Figure S12 NC-1 does not induce BST-2 degradation. (A) Western blot analysis of BST-2 protein level in HeLa cells treated with different concentrations of NC-1 for 24 h. Blots of three independent experiments are shown. (B) The amount of BST-2 was normalized to loading control and results were normalized to the DMSO control. Data are presented as means ± SEM, (n = 3).

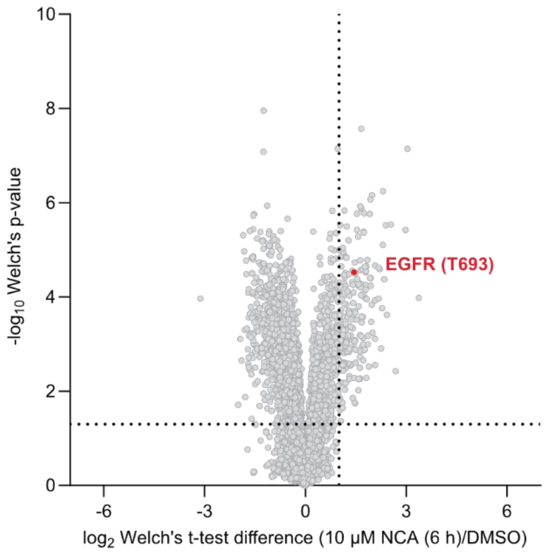

Figure S13 Influence of NCA on the global phosphoproteome. Volcano plot of phosphoproteome analysis of HeLa cells treated with 10 µM NCA for 6 h ($n = 4$). Phosphorylated peptides fulfilling the criteria p-value < 0.05 and log2 fold-change > 2 were considered significantly upregulated.

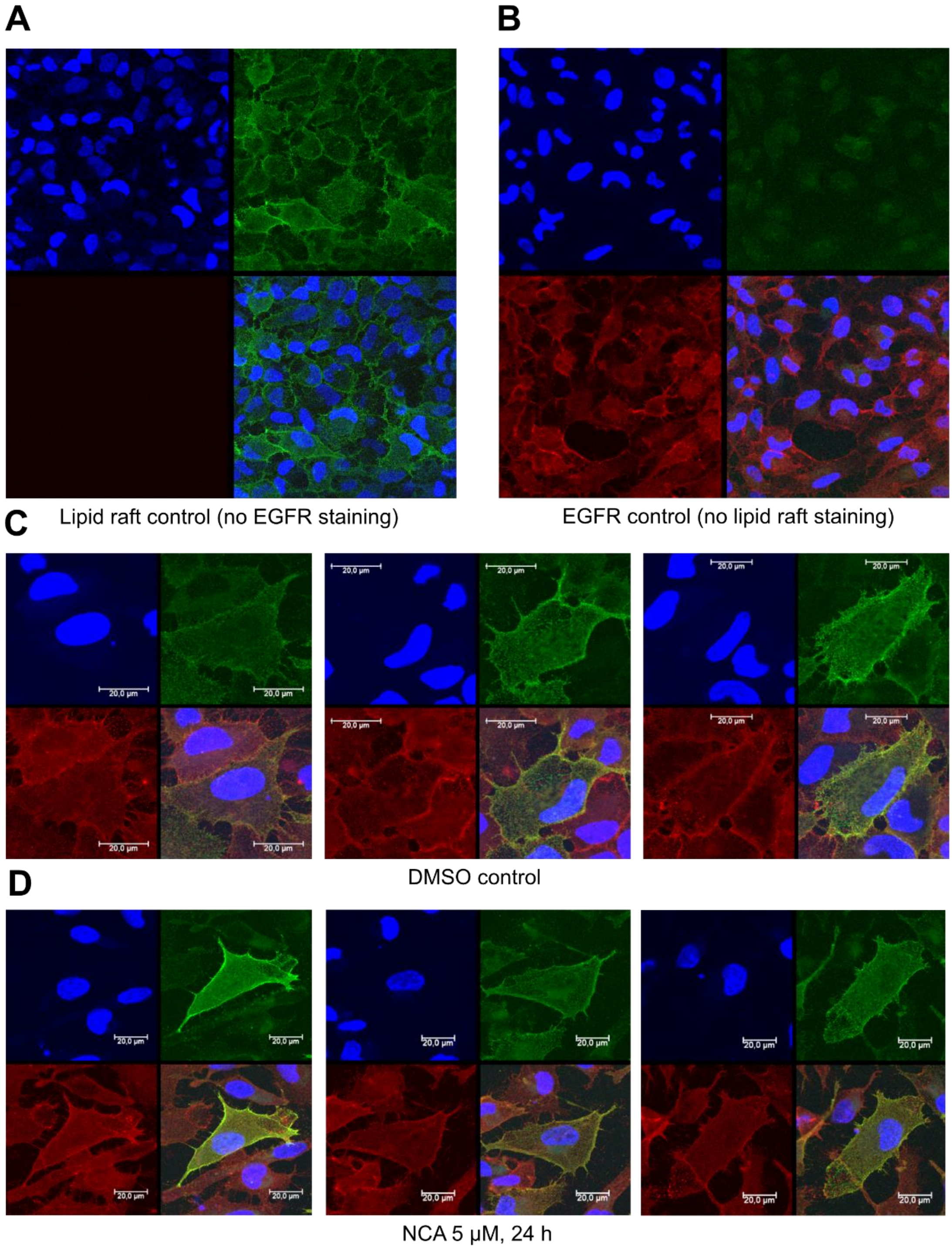

Figure S14 NCA affects the dynamics of EGFR protein by sequestering EGFR in lipid rafts (Figure 6). (A) Lipid raft control without EGFR staining, (B) EGFR control without lipid raft staining, (C) DMSO control, additional frames, (D) NCA (5 µM, 24 h) treated cells, additional frames. Binding of Cholera-Toxin-Subunit B (CT-B) is used as marker for lipid rafts.

2. Methods

2.1 Cell culture and cell lines

MDA-MB-231 cells for proteomic experiments were obtained from DSMZ and cultured in Dulbecco´s Modified Eagle´s Medium high glucose (DMEM) supplemented with heat-inactivated 10% (v/v) fetal bovine serum (FBS) and 2 mM L-glutamine. Cells were grown at 37 °C and 5% CO_2. HeLa cells were obtained from DSMZ and grown in DMEM supplemented with 10% (v/v) FBS and 2 mM L-glutamine. Cells were cultured at 37 °C with 5% CO_2 in a humidified incubator and were routinely tested for mycoplasma contamination.

2.2 MS-based proteomic procedures

2.2.1 *In situ* labeling in human cells

Cells for analytical labelling were seeded in 6-well plates, cells for preparative labelling were seeded in 15 cm dishes and were treated at 90% confluence with the probe (NC-1 or NC-4, stock solution in DMSO, 0.1% end concentration of DMSO in Medium) for 1 h at different concentrations (50 – 500 nM). For competition experiments, cells were preincubated with 25 µM of the natural product neocarzilin A (stock solution in DMSO, 0.1% end concentration of DMSO in Medium) for 1 h. The natural product solution was removed and replaced by 250 nM NC-4 (stock solution in DMSO, 0.1% end concentration of DMSO in Medium) for 1 h. The medium was removed, and the cells rinsed with cold PBS (1 mL for 6-well plates and 10 mL for 15 cm dishes). PBS was added to the cells, and they were scraped off and pelletized for 10 min at 600 rpm, 4 °C. The supernatant was aspirated. Cell lysis was performed using lysis buffer (1%(v/v) NP40 and 1% (w/v) sodium deoxycholate in PBS, 100 µL for pellets from 6-well plates and 1 mL for pellets from 15 cm dishes) at 4 °C for 15 min. Soluble and insoluble fractions have not been separated.

2.2.2 Analytical *in situ* labeling

Whole cell lysate was applied for click chemistry. Click chemistry was performed with TCEP (52 mM stock in ddH_2O, final conc. 1.0 mM), TBTA ligand (1.667 mM stock in DMSO/*t*BuOH = 1/4, final conc. 0.1 mM), $CuSO_4$ (50 mM stock in ddH_2O, final conc. 1.0 mM), rhodamine-azide (10 mM stock in DMSO; base click; Rh-N_3, final conc. 0.2 mM). The reaction was incubated at room temperature for 1 h and stopped by addition of 100 µL 2 x SDS loading buffer (63 mM Tris-HCl, 2% (v/v) glycerol, 139 mM sodium dodecylsulfate (SDS), 0.0025% (v/v) Bromophenol blue, 5% (v/v) 2-mercaptoethanol). For gel electrophoresis, 50 µL were applied per gel-lane on a SDS−PAGE gel (15.0% acrylamide). A Fujifilm LAS 4000 luminescent image analyser equipped with a Fujifilm LAS-300 camera, a Fujinon VRF43LMD3 lens and a 575DF20 filter was used for the detection of fluorescence.

2.2.3 *In situ* preparative labeling label-free quantification

For preparative labelling cells, were incubated with 25 µM NCA or DMSO for 1 h and subsequently with 250 nM NC-4 or DMSO for 1 h. Whole cell lysate was used. Protein amount after lysis was determined by BCA assay (Roti Quant, Roth) and adjusted to a final concentration of 1 µg in 1 mL. Click chemistry was performed with 0.20 mM Biotin-PEG3-N_3 (10 mM stock in DMSO, final conc. 0.2 mM, *Jena Bioscience*), TCEP (52 mM stock in ddH_2O, final conc. 0.52 mM), TBTA ligand (1.67 mM stock in DMSO/*t*BuOH = 1/4, final conc. 0.05 mM) and $CuSO_4$ (50 mM stock in ddH_2O, final conc. 0.5 mM). Click reaction was performed for 1 h at room temperature and MS sample preparation was performed as described before.[1] Proteins were precipitated with 10 mL MS-grade acetone at -80 °C. The precipitate was centrifuged down and washed with 500 µL MS-grade methanol twice. Enrichment was performed on 50 µL Pierce™ avidin-agarose beads (Thermo Scientific) in 0.2% SDS in PBS for 1 h at room temperature. The beads were washed three times with 0.2% SDS in PBS and

three times with PBS. The beads were resuspended in 200 µL denaturation buffer (7 M urea, 2 M thiourea in 20 mM Hepes, pH 7.5). Reduction and alkylation of the samples were performed at room temperature with DTT (1 mM) for 45 min, IAA (5.5 mM) for 30 min and DTT (4 mM) for 30 min. The samples were digested with 1 µL Lys-C (0.5 mg/mL, Fujifilm) 2 h at room temperature, 600 µL 50 mM TEAB buffer added and digested with 1.5 µL trypsin (0.5 mg/mL, Promega) overnight at 37 °C. 8 µL LC/MS-grade formic acid (FA) was added to stop the digest and the samples were desalted with Sep-Pak® C18 1 cc Vac cartridges (Waters Corp.). The columns were washed with MS-grade acetonitrile and 0.1% trifluoroacetic acid (TFA). Samples were loaded on the cartridges and washed with 0.1% TFA in MS-grade water, 0.5% FA in MS-grade water and eluted with elution buffer (20% H_2O, 0.5% FA in acetonitrile). Samples were lyophilized, stored at -80 °C and reconstituted in 30 µL 1% FA for MS/MS measurements.

2.2.4 Whole proteome analysis

Cells for whole proteome analysis were seeded in 15 cm dishes and were treated at 90% confluence with the natural product (NCA stock solution in DMSO, 0.1% end concentration of DMSO in medium) for different time periods (24 h and 48 h) at different concentrations (2.5 µM and 5 µM). The medium was removed, and the cells rinsed with cold PBS (10 mL). PBS was added to the cells, and they were scraped off and pelletized for 10 min at 600 rpm, 4 °C. The supernatant was aspirated. Cell lysis was performed using lysis buffer (1%(v/v) NP40 and 1% (w/v) sodium deoxycholate in PBS, 1 mL) at 4 °C for 15 min. Soluble and insoluble fractions have not been separated. The samples were adjusted after BCA to 200 µL, 0.5 mg/mL protein and proteins were precipitated by the addition of MS-grade acetone (1 mL, -80 °C). The precipitate was centrifuged down and washed with 500 µL MS-grade methanol twice. The proteins were resuspended in 200 µL in denaturation buffer (7 M urea, 2 M thiourea in 20 mM Hepes, pH 7.5). Reduction and alkylation of the samples were performed with TCEP (5 mM) for 1 h at 37 °C and with IAA (10 mM) for 30 min and DTT (10 mM) for 30 min at room temperature. The samples were digested with 1 µL Lys-C (0.5 mg/mL, Fujifilm) for 4 h at room temperature, 600 µL 50 mM TEAB buffer added and digested with 2 µL trypsin (0.5 mg/mL, Promega) overnight at 37 °C. The digest was stopped by the addition of 10 µL LC/MS-grade formic acid (FA) and the samples were desalted with Sep-Pak® C18 1 cc Vac cartridges (Waters Corp.). The cartridges were washed with MS-grade acetonitrile and 0.1% trifluoroacetic acid (TFA) in MS-grade water. Samples were loaded on the cartridges and washed with 0.1% TFA, 0.5% FA in MS-grade water and eluted with elution buffer (20% H_2O, 0.5% FA in acetonitrile). Samples were lyophilized and stored at -80 °C.

2.2.5 MS/MS measurement Orbitrap Fusion

Samples were dissolved in 1% FA in MS-grade water, sonicated for 15 min and filtered through a 0.22 µm Ultrafree-MC® centrifugal filter (Merck, UFC30GVNB) equilibrated with 1% FA MS-grade water. Samples were analyzed by LC-MS/MS using an UltiMate 3000 nano HPLC system (Dionex) equipped with an Acclaim C18 PepMap100 (75 µm ID x 2 cm) trap and a 25 cm Aurora Series emitter column (25 cm × 75 µm ID, 1.6 µm FSC C18) (Ionopticks) (column oven set to 40 °C) coupled to an Orbitrap Fusion (Thermo Fisher) in EASY-spray setting. Samples were loaded on the trap column with a flow rate of 5 µL/min with 0.1% TFA in MS-grade water and washed for 10 min. The peptides were transferred onto the separation column and were separated using a 132 min gradient (buffer A: 0.1% FA in MS-grade water, buffer B: 0.1% FA in acetonitrile, gradient: to 5% buffer B in 7 min, to 22% buffer B in 105 min, to 35% buffer B in 10 min and to 90% buffer B in 10 min. The column was washed with 90% buffer B for 10 min and reequilibrated with 5% buffer B for 10 min). Peptides were ionized using a nanospray source at 1.7-1.9 kV at a transfer capillary temperature of 275 °C. The instrument was used in top speed data-dependent mode and the cycle time between master scans set to 3 s. MS full scans were recorded at a resolution of R = 120,000 and an automatic gain control

(AGC) ion target value of $2\times e^5$ in a scan range of 300 – 1500 m/z with a maximum injection time of 50 ms and a RF lens amplitude of 60%. Precursors with intensities higher than $5\times e^3$ and charge states between 2 and 7 were selected for fragmentation in the higher-energy collisional dissociation (HCD) cell (30% collision energy). MS^2 scans were recorded in the ion trap operating in a rapid scan mode. The isolation window was set to 1.6 m/z. For enriched samples, the AGC target was set to $1\times e^4$ with a maximum injection time of 100 ms and for complex samples, AGC target of $1\times e^4$ with a maximum injection time of 35 ms was applied.

2.2.6 Bioinformatics and statistics

Processing of MS raw data was done with the software MaxQuant version 1.6.2.10. For identification of peptides, MS/MS spectra were searched against the Uniprot database for Homo sapiens (taxon identifier: 9606, downloaded on 30.09.2020, canonical).[2, 3] For MaxQuant mostly default settings were used (trypsin/P as digest enzyme, max. 2 missed cleavages, oxidation (M) and protein N-term acetylation as variable modifications, carbamidomethylation (C) as fixed modification, min. peptide length 7). The main search was conducted with 4.5 ppm for precursor mass tolerance and 0.5 Da for fragment mass tolerance. Protein identification was conducted with the following settings: PSM FDR 0.01, Protein FDR 0.01, min. razor + unique peptides: 2, razor protein FDR enabled, second peptides enabled. The "match between run" option (0.7 min match and 20 min alignment time windows) was enabled. Label free quantification (LFQ) was used for all samples. The built-in LFQ algorithm in MaxQuant software (MaxLFQ)[4] was used with a minimal ratio count of 1. The mass spectrometry proteomics data have been deposited to the ProteomeXchange Consortium[5] via the PRIDE9 partner repository with the dataset identifier PXD050453.

The statistical analysis of the resulting data was done with the Perseus software[6] (version 1.6.5.0). Normalized LFQ intensities from the proteinGroups.txt table were used for further analysis. First, the data was filtered by "filtering by categorical columns", namely the columns "identified by site", "reverse" and "contaminants". Log2 transformation and categorical annotation of treated samples and control were performed. The data was filtered against 70% of valid values in at least one group. Missing values were imputed from normal distribution (width 0.3, down shift 1.8, for the total matrix). Two-sample Student's t-tests including permutation based false discovery rate correction (FDR = 0.05) were performed. Volcano plots were generated by plotting student's t-test difference (treated/control) against t-test p-value (treated/control). Proteins with a p-value of < 0.05 and an enrichment factor of > 4 ($\log2(x) = 2$) were considered as significantly enriched.

2.2.7 Ingenuity pathway analysis (IPA)

Ingenuity Pathway Analysis (IPA) of the proteomics data was done with QIAGEN IPA (QIAGEN Inc., https://digitalinsights.qiagen.com/IPA). For IPA[7], proteomics data was first processed as described in section 2.2.6. p-values and log2 fold changes from the Student's t-tests were used for pathway analysis. In IPA, core analysis was performed with the setting "human" as species and a p-value cutoff of 0.05.

2.3 Phosphoproteomic procedures

2.3.1 Sample Preparation

Hela cells were grown in 100 mm dishes and treated at 90 % confluence with 2.5 and 10 µM NCA or DMSO for 6 or 48 hours, respectively. Subsequently pre-cooled lysis buffer consisting of 40 mM Tris-HCl pH 7.6, 8 M Urea lysis buffer + protease (cOmplete™ mini, EDTA-free protease inhibitor cocktail, Roche) and phosphatase inhibitors (Phosphatase inhibitor cocktail 1,2 + 3, Sigma-Aldrich) were added to the dishes and incubated for 10 minutes on ice. Cells were mixed with a cell scraper, transferred to 1.5 mL tubes, homogenates clarified by centrifugation for 5 minutes at 18,000 x g and protein concertation determined by Bradford assay. 200 µg protein input (c = 1.14 µg/µL) were reduced with 10 mM DTT at 30 °C for 45

min followed by alkylation of cysteines with 55 mM chloroacetamide for 30 min at room temperature. Samples were diluted 1:5 with digestion buffer (50 mM Tris-HCl pH 8.5, 2 mM $CaCl_2$) before two-step digestion for 2 h and over-night with trypsin at a 1:100 (w/w) ratio at 37 °C and 700 rpm. On the next day, samples were acidified to 1% formic acid (FA) followed by desalting on SepPAC50 columns (Waters). Peptides were eluted with 50% acetonitrile (ACN), 0.1% FA and vacuum dried. Dried peptides were reconstituted in 50 HEPES pH 8.5 and labelled with TMT10plex-labeling reagent (Lot. XA338782) as described previously[8] with a protein to tandem mass tag (TMT) reagent ratio of 1:1. Labelled samples were pooled, vacuum dried and reconstituted in 0.07% trifluoracetic acid (TFA) prior to desalting on SepPAC500 columns. Peptides were eluted with 50% ACN, 0.07% TFA, 5% of the volume transferred to a new tube for whole proteome analysis and both tubes vacuum dried. The TMT-pool peptide mix for whole proteome analysis was reconstituted in 25 mM NH_4FA pH 10 and fractionated on self-packed stage tips (3M Empore™, five disks, Ø 1.5 mm, C18 material) as described previously[9]. Peptides were sequentially eluted with 5%, 10%, 15%, 17.5% and 50% ACN in 25 mM NH_4FA pH 10 and subsequently pooled to four fractions (50%+5%, 10%, 15%, 17.5%+Flow trough). The TMT-pool peptide mix for phosphoproteome analysis was reconstituted in 0.07% TFA, 30% ACN and phosphoenrichment performed by immobilized metal ion affinity chromatography (IMAC) enrichment on a Fe(III)-loaded ProPac IMAC-10 column (Thermo Scientific) as described previously.[10] The phosphopeptide fraction was vacuum dried, reconstituted with 0.1% FA and loaded on self-packed C18 stage tips (3M Empore™, five disks, Ø 1.5 mm). After washing with 0.1% FA the pH was changed with 25 mM NH_4FA pH 10 and the flow through collected. Peptides were sequentially eluted as described above and pooled to four fractions. Both whole proteome and phosphopeptide fractions were vacuum dried and stored at -20 °C until MS measurement.

2.3.2 Mass spectrometric measurement

LC-MS analysis was performed on a Dionex 3000 (Thermo Fisher Scientific) coupled online to an Orbitrap Eclipse mass spectrometer (Thermo Fisher Scientific). Samples were reconstituted in 0.1% FA and 1/3 or 1/2 of the sample injected for whole proteome or phosphoproteome analysis, respectively. Peptide loading and washing were done on a trap column (100 µm i.d. x 2 cm, packed in-house with Reprosil-Pur C18-GOLD, 5 µm resin, Dr. Maisch) at a flow rate of 5 µL/min in 100% loading buffer (0.1% FA) for 10 min. Peptide separation was performed on an analytical column (75 µm i.d. x 40 cm packed in-house with Reprosil-Pur C18, 3 µm resin, Dr. Maisch) at a flow rate of 300 nL/min (solvent A: 0.1% FA, 5% DMSO in HPLC grade water; solvent B: 0.1% FA, 5% DMSO in ACN).[11] The phosphoproteome fractions were measured with an 80 min two-step gradient from 4% to 22.5% and 32% ACN. The MS instrument was operated in sensitive data-dependent MS3-mode. Full scans (MS1) were recorded from 360 to 1800 m/z with a resolution of 60k in the Orbitrap in profile mode using a normalized AGC target of 100% and maxIT of 50 msec. The cycle time was set to 3 sec. Selected precursors were isolated with 0.7 Th and fragmented in the linear ion trap by CID-targeting the precursor and precursor H_2PO_4 in parallel (multistage-activation) with a q-value of 0.25, 35% CE and 10 ms activation time. MS2 spectra were acquired with 30k resolution in the Orbitrap. The MS2 normalized AGC target was set to 300%, the maxIT to 60 msec and dynamic exclusion time to 90 sec. TMT reporter ions were measured in a consecutive MS3 scan based on the previous MS2 scan. Precursor ions were isolated with a window of 1.2 Th and MSA-fragmented identically to the previous MS2 scan. The top 10 fragment ions of the MS2 scans were isolated in the ion trap in parallel (synchronous precursor selection) and fragmented via HCD using an NCE of 55%. The MS3 spectra were recorded with 50k resolution in the Orbitrap. MS3 normalized AGC target was set to 500%, and the maxIT to 120 msec. The whole proteome fractions were measured with an 80 min linear gradient from 8% to 34% ACN. The MS instrument was operated in fast data-dependent MS3-mode. Full scans (MS1) were recorded from 360 to 1500 m/z with a resolution of 60k in the Orbitrap in

profile mode using a normalized AGC target of 100% and maxIT of 50 msec. The cycle time was set to 3 sec. Selected precursors were isolated with 0.7 Th and HCD fragmented with an NCE of 34%. MS2 spectra were acquired with 15k resolution in the Orbitrap. The MS2 normalized AGC target was set to 100%, the maxIT to 2 msec and dynamic exclusion time to 90 sec. TMT reporter ions were measured in a consecutive MS3 scan based on the previous MS2 scan. Precursor ions were isolated with a window of 1.2 Th and HCD-fragmented identically to the previous MS2 scan. The top 10 fragment ions of the MS2 scans were isolated in the ion trap in parallel (synchronous precursor selection) and fragmented via HCD using an NCE of 55%. The MS3 spectra were recorded with 30k resolution in the Orbitrap. MS3 normalized AGC target was set to 200%, and the maxIT to 54 msec.

2.3.3 Bioinformatics and statistics

Whole proteome and phospho TMT peptide identification and quantification were performed with MaxQuant (version 1.6.3.3), with MS3 standard settings unless otherwise described.[2] Isotope impurities of the TMT lot were specified to allow the automated correction of TMT intensities. Raw files were searched against the human reference proteome (UP000005640, 75,777 entries, download 01/2021) and common contaminants. Carbamidomethylated cysteine was set as fixed modification and oxidation of methionine, and N-terminal protein acetylation as variable modifications. Phosphorylation of serine, threonine or tyrosine were allowed as variable modification specifically for the phospho-enriched samples. Trypsin/P was specified as the proteolytic enzyme with up to two missed cleavage sites allowed. Results were adjusted to 1% site, peptide spectrum match and protein false discovery rate (FDR) employing a target-decoy approach using reversed protein sequences.

MaxQuant output tables were filtered for contaminants, reversed sequences, and proteins, which were only identified based on modified peptides. Protein abundance estimation was based on corrected TMT reporter intensities. For data analysis, the 6 h and 48 h TMT sets were total sum normalized, followed by a row-wise normalization based on one of the shared common TMT channels to remove batch effects between the two TMT sets. Unless otherwise stated, displayed protein abundances were log2 transformed. The mass spectrometry proteomics data have been deposited to the ProteomeXchange Consortium[5] via the PRIDE9 partner repository with the dataset identifier PXD050453.

2.4 Cell based assays

2.4.1 Compounds and treatment

Neocarzilin A was dissolved in DMSO and stored at -20 °C in small aliquots. Cells were seeded overnight before treatment and incubated with compounds at indicated concentrations and time points. DMSO was diluted in the corresponding culture medium as solvent control and did not exceed 0.1% (v/v) in any of the experiments.

2.4.2 Crystal violet staining assay

To evaluate the proliferative capacity, crystal violet staining assay was performed to detect the effect of compounds on cell proliferation in HeLa cells. Briefly, HeLa cells were seeded in 96-well plates at a density of 3000 cells/well the night before treatment and incubated with indicated concentrations of test compounds or DMSO. After 72 h, the medium was discarded and cells were washed with PBS and then stained with crystal violet solution (0.5% crystal violet, 20% methanol) for 10 min. Then cells were gently washed with water and dried, followed by dissolving in sodium citrate solution (0.1 M sodium citrate, 50% ethanol). Absorbance was measured at 550 nm by a Sunrise™ microplate reader (Tecan, Crailsheim, Germany). For analysis, values of day 0 were subtracted, and results were normalized to the DMSO control, which was set to 100% proliferation.

2.4.3 xCELLigence® assay

For real time monitoring of single cell migration, pre-stimulated medium at the indicated concentrations and medium without FCS as negative control were added to the lower chamber of an xCELLigence® CIM-plate 16, which was subsequently assembled and equilibrated according to manufacturer's protocol (ACEA Biosciences, San Diego, CA, USA). HeLa cells were detached from the culture flask and resuspended in pre-stimulated medium without FCS and 4.0 x 10^4 cells were seeded in the upper chamber of the equilibrated CIM-plate. After cells were allowed to settle onto the microporous (8 µm) membrane for 30 min at RT, the CIM-plate was placed into the xCELLigence instrument and migration towards growth medium containing 10% FCS as chemoattractant was recorded over 18 h. The xCELLigence device measures the impedance and transforms it into the cell index (CI), a dimensionless parameter, which is proportional to the number of migrated cells. The slope of the CI curves was evaluated as migration rate with the RTCA xCELLigence software (ACEA Biosciences, San Diego, CA, USA). The results of each biological replicate were normalized to the DMSO control, which was set to 100% migration rate.

2.4.4 Quantification and statistical analysis

All repeated experiments are independent and biological replicates unless indicated otherwise. Flow cytometry data were processed with FlowJo 7.6. Confocal images and Western blot densitometry were analyzed using ImageJ. Statistical analyses were performed with GraphPad Prism 8/9. Ordinary one-way ANOVA with a post-hoc Tukey's test or Dunnett's test or unpaired t-test with Welch's correction was performed and significance is shown as $^{ns}p > 0.12$, $^{*}p<0.033$, $^{**}p<0.002$, $^{***}p<0.001$. All shown graphs are presented as means ± standard error of mean (SEM).

2.5 Immunological procedures

2.5.1 Antibodies

The following antibodies were used.

Table S1. List of used antibodies.

Antibodies	SOURCE	IDENTIFIER
Goat Anti-Mouse IgG1, HRP-linked	Abcam	Cat# ab97240, RRID:AB_10695944
Goat anti-rabbit IgG (H+L), HRP-linked	Jackson ImmunoResearch Labs	Cat# 111-035-144, RRID: AB_2307391
Goat anti-rat IgG Alexa Fluor™ 546	Thermo Fisher Scientific	Cat# A-11081, RRID:AB_2534125
Mouse monoclonal anti- Phospho-STAT3 (Tyr705)	Cell Signaling Technology	Cat# 9138, RRID:AB_331262
Mouse monoclonal anti-BST-2	Santa Cruz Biotechnology	Cat# sc-390719
Rabbit monoclonal Anti-EGF Receptor	Cell Signaling Technology	Cat# 4267, RRID:AB_2895042
Rabbit monoclonal Phospho-EGF Receptor (Tyr1068)	Cell Signaling Technology	Cat# 48576
Rabbit polyclonal anti-STAT3	Cell Signaling Technology	Cat# 9132, RRID:AB_331588
Rat monoclonal anti-EGFR	Abcam	Cat# ab231, RRID:AB_2293306
Mouse monoclonal anti-VAT-1	Santa Cruz Biotechnology	Cat# sc-515705
Rabbit anti-ubiquitin	Cell Signaling Technology	Cat# 3933
Anti-HA HA.11 Clone 16B12	Covance	

2.5.2 Detection of BST-2 surface level

To analyze the effect of NCA on BST-2 surface level, flow cytometry was used. 1.0 x 10^5 cells per well were seeded into 12-well plates. After indicated treatment of compounds, cells were trypsinized and collected into 15 mL falcon tubes, and then cells were washed by centrifugation (350 g, 5 min at 4 °C) in PBS. Next, cells were resuspended in 150 µL of diluted primary

antibody, which was diluted in antibody dilution buffer (1% FBS in PBS, 1:200 dilution) and incubated on ice in a shaker shaking gently for 1 h in the dark. Subsequently, cells were washed twice by centrifugation (350 g, 5 min at 4 °C) in pre-chilled PBS to remove the excess antibody and then resuspended in 100 µL of diluted Alexa Fluor™ 488-conjugated secondary antibody (prepared in antibody dilution buffer). After 30 min incubation on ice in the dark, the cells were washed twice by centrifugation (350 g, 5 min at 4 °C) in pre-chilled PBS to remove excess dye and finally resuspended in 200-500 µL of pre-cooled PBS and analyzed on the FACSCanto II (BD, New Jersey, USA). In parallel, the anti-mouse IgG served as isotype control.

2.5.3 Western Blot

Cells were seeded into 6-well plates at a density of 4.2 x 10^5 cells/well. After specific treatment, cells were harvested and the protein concentration was determined using a Bradford protein assay and mixed with 5x SDS sample buffer before being resolved by SDS-polyacrylamide gel electrophoresis (SDS-PAGE) and transferred to PVDF membrane. Membranes were blocked with 5% (w/v) BSA (Anprotec, cat # AC-AF-0023) in TBS-T buffer (24.8 mM Tris-base, 190 mM NaCl, and 0.1% Tween-20) for 2 h at RT and subsequently incubated with appropriate primary antibodies overnight at 4 °C. The membranes were washed with TBS-T three times before incubating with HRP-conjugated secondary antibodies for 2 h at RT. After washing with TBS-T, membranes were incubated with ECL solution (100 mM pH 8.5 Tris, 2.5 mM luminol, 1 mM coumaric acid, and 17 µM H_2O_2) and the chemiluminescence was visualized by using a ChemiDoc™ touch imaging system.

2.5.4 Lipid rafts staining

Cells were seeded in ibidi 8-well µ-slides overnight before indicated treatment. Cells were washed with pre-chilled complete growth medium and incubated with the fluorescent CT-B conjugate working solution (1 µg/mL in chilled complete growth medium, Thermo Fisher Scientific, cat # V34403). After 10 min at 4 °C, cells were washed gently with chilled PBS three times. Afterwards, cells were incubated with the chilled anti-CT-B antibody working solution (1:200 dilution) for 15 min at 4 °C. After this incubation, cells were washed gently with chilled PBS before fixation in chilled 4% PFA for 15 min at 4 °C. The cells were washed with PBS and blocked in 1% (w/v) BSA in PBS for 1 h at RT followed by incubating with EGFR antibody (1:200 dilution) overnight at 4 °C. On the next day, cells were washed three times with PBS and then incubated with Alexa fluor 546 coupled secondary antibody (1:400 dilution) and Hoechst 33342 (5 µg/mL) for 1 h at RT. Cells were submerged with one drop of FluorSave reagent mounting medium and covered with a glass coverslip after being washed again with PBS three times. Images were captured by a Leica SP8 Inverted confocal microscope (Leica Microsystems, Wetzlar, Germany).

2.5.5 Immunoprecipitation

BST-2 was overexpressed in HeLa cells using the plasmid pCMV-HA.Tetherin, which was a gift from Paul Spearman (Addgene plasmid # 41068 ; http://n2t.net/addgene:41068 ; RRID:Addgene_41068). 24h after transfection, cells were lysed, and the BST was enriched using a mouse anti-HA antibody (HA.11 Clone 16B12, Covance) in combination with magnetic protein A beads from Miltenyi (Bergisch Gladbach, Germany) according to the manufacturer´s instructions. The precipitates were subjected to gel electrophoresis, and subsequently a Western blot was performed. Protein loading in the molecular weight range were BST-2 is to be expected was measured by the stain free technology and used for normalization. Ubiquitination of BST-2 was then detected by use of a rabbit anti-ubiquitin antibody (#3933, Cell Signaling Technology).

2.6 Genetic Methods

2.6.1 Generation of a VAT-1 knockout cell line

To generate a VAT-1 knockout cell line in HeLa cells, the CRISPR/Cas9 system was employed. Therefore, a mixture of three plasmids (sc-405997, Santa Cruz Biotechnology, Dallas, TX, USA) containing different guideRNAs (gRNA1: CTATCACACGACTGACTACG, gRNA2: CAGGGCCATCAGGTTCCGTT, gRNA: GTGATGGTGTTGAACCGGTC) and a GFP tag for visualizing successfully transfected cells was purchased. Plasmids were transfected according to the Lipofectamine™ 3000 manufacturers' instruction and cells incubated for 48 h. Then, cells were detached, counted and 1x10^6 cells resuspended in 2 mL pre-warmed PBS in a sterile FACS tube for sorting (Flow Cytometry Facility, Gene Center, LMU, Munich, Germany) with the following gating strategy: gate P1 to exclude cell debris, P2 + P3 to enrich single cells and exclude cell clusters, P4 for living cells (DAPI negative) and P5 for GFP positive cells (= successfully transfected). The sorted cells were directly seeded in collagen G coated 96-well plates as single cells and regularly screened for growing colonies. Colonies were expanded and sequentially transferred to 24-, 6-well plates and finally to a 25 and 75 cm^2 flask. At this point, protein samples for knockout validation via Western blotting were taken (Figure S3A). Clonal cell lines 1A2, 1A3 were expanded, frozen as cryo-stocks and used for further experiments.

2.6.2 Generation of a BST-2 knockout cell line

The knockout (KO) of BST-2 in HeLa cells was conducted with the CRISPR-Cas9 system. In brief, gRNAs targeting human BST-2 were cloned into the vector PX459 V2.0 which was a gift from Feng Zhang[12] (Addgene plasmid # 62988; http://n2t.net/addgene:62988; RRID: Addgene_62988). Two gRNAs were used to increase KO efficiency, sequences of gRNA1: 5'-CGCTTATCCCCGTCTTCCAT-3', gRNA2: 5'-CCCCCAGAATCACGATGATC-3'. Single-strand gRNAs were dissolved in 100 µM annealing buffer (10 mM Tris pH 7.5, 50 mM NaCl and 1 mM EDTA) and then mixed with equal volumes of forward and reverse primers in a PCR tube. The mixture was then heated to 95 °C for 2 min in a thermocycler and then was cooled gradiently to 25 °C for 45 min. Meanwhile, the vector PX459 V2.0 was digested by restriction enzyme BbsI at 37 °C for 2 h, and then annealed gRNAs were ligated into the vector using T4 ligase (Table S1) at 4 °C overnight, respectively. The ligation solution was transformed into DH5α. Briefly, 50 µL DH5α competent cells were thawed on ice and 10 µL of the plasmid-DNAs solution was added and mixed well. The mixture was placed on ice for 30 min and heated at 42 °C for 90 s, and then put on ice for another 2 min. Next, 300 µL LB medium was added into the tube and incubated at 37 °C for 1 h. Finally, the whole bacterial suspension was plated on an agar plate containing 100 µg/mL ampicillin and incubated at 37 °C for 1 h and then inverted for 12-16 h. Transformed DH5α colonies were picked and sequenced using human U6-forward primer 5'-GAGGGCCTATTTCCCATGATT-3'. HeLa cells were seeded into a 6-well plate and cell confluency was around 70% before transfection. The next day, cells were transfected with the plasmids using Lipofectamine 3000 for 2 days as described above before puromycin selection (2 µg/mL) for another 2 days. Afterwards, the single cells isolation was performed with the serial dilution method. Briefly, the transfected cells were seeded 1 cell/well into 96-well plates for 7-10 days in culture, and single-cell colonies were expanded and frozen. Successful KO of BST-2 in identified clones was confirmed by Western blot to detect BST-2 protein levels in wild-type (WT) control cells and BST-2 KO candidates.

2.6.3 Quantitative real-time PCR analysis

Cells were treated with DMSO or NCA for 8 h and mRNA from each well was isolated using the RNeasy® Mini Kit according to the manufacturer. The mRNA concentration was determined by a Nanodrop® Spectrophotometer followed by reverse transcription of mRNA to cDNA with the High-Capacity cDNA Reverse Transcription Kit (Applied Biosystems, Waltham, USA) as described by the manufacturer. Subsequently, the quantitative real-time polymerase chain

reaction (qPCR) was performed and a QuantStudio™ 3 Real-Time PCR System was used. In brief, primers were designed using the Harvard primer bank (https://pga.mgh.harvard.edu/primerbank/) and the forward and reverse primers for GAPDH gene (FW : GGAGCGAGATCCCTCCAAAAT, RE : GGCTGTTGTCATACTTCTCATGG), for the BST-2 gene (FW: CACACTGTGATGGCCCTAATG, RE: GTCCGCGATTCTCACGCTT). Primers were purchased from Metabion (Planegg, Germany), the qPCR reaction solution was added in each well of the MicroAmp®. Fast Optical 96-Well Reaction Plate and sealed with a foil before centrifuging (1000 rpm, 1 min). For the data analysis, GAPDH served as the control gene and the $\Delta\Delta C_T$ method was used to quantify changes in mRNA levels.

3 Supplementary Tables

Table S2. Proteins matching the criteria (p-value < 0.05, log2 fold-change > 2) of LFQ ABPP experiment with 250 nM NC-4 in MDA-MB-231 (1 h) (Volcano plot Figure S2B).

protein name	gene name	enrichment	p-value	sequence coverage [%]
Heme oxygenase 2	HMOX2	6.79	4.39	55.7
Vesicle amine transport protein 1 homolog	VAT1	6.13	5.47	41.7
Bone marrow stromal antigen 2	BST2	5.61	4.95	18.3
Reticulon-4	RTN4	4.41	2.44	14.9
Endonuclease domain-containing 1 protein	ENDOD1	3.84	2.31	21.2
Cytochrome b5 type B	CYB5B	3.72	3.55	45.2
Protein DBF4 homolog B	DBF4B	3.41	1.62	6.5
Zinc finger protein 185	ZNF185	2.49	1.91	34.4
Reticulon-1	RTN1	2.47	1.88	3.6
Transmembrane 9 superfamily member	TM9SF2	2.20	1.62	5.6
Cysteine dioxygenase type 1	CDO1	2.13	1.41	7.5
Prenylcysteine oxidase 1	PCYOX1	2.10	1.54	9.3

Table S3. Proteins matching the criteria (p-value < 0.05, log2 fold-change > 2) of LFQ ABPP experiment with 250 nM NC-4 in HeLa (1 h) (Volcano plot Figure 2B).

protein name	gene name	enrichment	p-value	sequence coverage [%]
Heme oxygenase 2	HMOX2	5.45	5.44	46.8
Bone marrow stromal antigen 2	BST2	5.23	3.32	18.3
Vesicle amine transport protein 1 homolog	VAT1	3.93	3.85	48.9
Prostaglandin E synthase	hCG_30600	3.42	1.46	5.6
Reticulon-1	RTN1	3.40	4.47	2.4
GTPase-activating protein and VPS9 domain-containing protein 1	GAPVD1	3.14	1.55	6.6

CD63 antigen	CD63	2.95	1.32	13.5
Cytochrome b5 type B	CYB5B	2.71	4.75	45.2
CD44 antigen	CD44	2.69	1.30	20.9
Amino acid transporter	SLC1A1	2.66	2.95	5.5
cDNA FLJ58568	C16orf58	2.54	3.34	9.5
PRA1 family protein	PRAF2	2.43	1.81	20.8
Coactosin-like protein	COTL1	2.36	1.59	21.9
Endonuclease domain-containing 1 protein	ENDOD1	2.27	2.55	14.2
Pirin (Iron-binding nuclear protein)	PIR	2.15	2.08	15.2
cDNA FLJ46477 fis	MCAM	2.11	1.99	15.1
UPF0729 protein C18orf32	C18orf32	2.02	3.12	13.2

Table S4. Proteins matching the criteria (p-value < 0.05, log2 fold-change < -2) of LFQ ABPP competition experiment with 25 µM NCA (1 h) and 250 nM NC-4 (1 h) in HeLa (Volcano plot Figure S2C).

protein name	gene name	enrichment	p-value	sequence coverage [%]
Heme oxygenase 2	HMOX2	-5.62	5.40	46.8
NOC3-like protein	NOC3L	-4.29	2.15	5
Vesicle amine transport protein 1 homolog	VAT1	-3.30	2.71	48.9
Rho GTPase-activating protein 1	ARHGAP1	-3.11	1.56	27.6
Reticulon-1	RTN1	-2.92	3.38	2.4
Niemann-Pick C1 protein	NPC1	-2.89	2.02	5.6
V-type proton ATPase subunit	ATP6V0D1	-2.87	1.48	17.7
cDNA FLJ58568	C16orf58	-2.81	3.34	9.5
GTPase-activating protein and VPS9 domain-containing protein 1	GAPVD1	-2.74	1.58	6.6
Bone marrow stromal antigen 2	BST2	-2.69	3.75	18.3
Prostaglandin E synthase	hCG_30600	-2.66	1.60	5.6
cDNA FLJ77391	EHD4	-2.46	1.42	20.9
Solute carrier family 39 (Zinc transporter)	SLC39A10	-2.35	1.74	6.4
2-Hydroxyacylsphingosine 1-beta-galactosyltransferase	UGT8	-2.32	4.09	9.6
60S ribosomal protein L27	RPL27	-2.27	1.71	15.9
Carboxypeptidase	SCPEP1	-2.25	2.26	4.7
Acyl-CoA (8-3)-desaturase	FADS1	-2.22	1.30	13.5
Transmembrane protein 201	TMEM201	-2.19	2.86	3.8
Phosphoinositide phospholipase C	PLCD3	-2.18	1.70	8.9
Amino acid transporter	SLC1A1	-2.13	2.19	5.5
Isopentenyl-diphosphate Delta-isomerase 1	IDI1	-2.10	1.40	14.1
Prenylcysteine oxidase 1	PCYOX1	-2.10	1.94	20.6
Reticulon-4	RTN4	-2.09	2.14	15.9

Catechol O-methyltransferase	COMT	-2.06	1.55	15.8
Peptidyl-prolyl cis-trans isomerase	PPIL1	-2.05	1.68	29.5
Disco-interacting protein 2	DIP2B	-2.04	2.29	8.8
Carnitine O-palmitoyltransferase	CPT1A	-2.04	1.45	16.4

Table S5. Proteins matching the criteria (p-value < 0.05, log2 fold-change > 2) of LFQ ABPP experiment with 250 nM NC-4 in HeLa VAT-1 knockout cells (1 h) (Volcano plot Figure 2E).

protein name	gene name	enrichment	p-value	sequence coverage [%]
Heme oxygenase 2	HMOX2	6.56	5.52	46.2
Bone marrow stromal antigen 2	BST2	6.27	5.24	18.3
CD63 antigen	CD63	4.40	3.29	13.5
Reticulon-3	RTN3	3.58	2.15	5.4
CD44 antigen	CD44	3.15	1.62	31.7
Cytochrome b5 type B	CYB5B	2.78	4.77	45.2
Serpin peptidase inhibitor	SERPINE2	2.72	1.33	8.7
cDNA FLJ56823		2.36	2.19	9.4
Tubby-related protein 2	TULP2	2.23	1.60	12.6
Ankyrin repeat and KH domain-containing protein 1	ANKHD1	2.13	1.90	2.2
Dolichol-phosphate mannosyltransferase subunit 1	DPM1	2.10	1.47	8.5
Mitochondrial aldehyde dehydrogenase 2 variant	ALDH2	2.10	3.26	36.8
Reticulon-4	RTN4	2.06	2.49	14.3
MICOS complex subunit	APOOL	2.03	1.69	17.5

4 Synthetic procedures

4.4 General Methods and Materials

All reagents and solvents were purchased in reagent grade or higher from commercial vendors (Sigma-Aldrich, Thermo Fisher Scientific Inc., Merck KGaA, Alfa Aesar, Roth, VWR International, Fluka, Acros Chemicals) and were used as delivered without further purification. Technical grade solvents were distilled prior to use. All air and/or water sensitive reactions were conducted under argon atmosphere using flame dried glassware using standard Schlenk-techniques. Merck silica-gel 60 F254 plates were used for analytical thin-layer chromatography (TLC). The spots were visualized using short wave UV light (λ=254 nm and 366 nm) or a $KMnO_4$-stain (1.50 g $KMnO_4$, 10.0 g K_2CO_3, 1.25 mL $NaOH_{aq}$ (10 wt-%), 200 mL ddH_2O) or a Dinitrophenylhydrazine-stain (12.0 g 2,4-Dinitrophenylhydrazine, 60 mL H_2SO_4 (98%), 80 mL ddH_2O, 200 mL EtOH). Preparative TLC was performed using TLC Silica gel 60 F254 (20 x 20 cm) plates from Merck KGaA or pre-coated TLC plates DIL G-200 from MACHEREY-NAGEL GmbH & Co. KG. Flash chromatography was performed Silica gel 60 (particle size = 40–63 µM) from Merck KGaA with compressed air. Proton-NMR spectra were recorded on Avance-III (AV-HD300, AV-HD400 or AV-HD500) NMR systems (Bruker Co.) at room temperature with deuterated chloroform ($CDCl_3$), dimethylsulfoxid (DMSO-d_6) or acetonitril (CD_3CN) as solvents and referenced to the residual proton signal of the corresponding deuterated solvent ($CDCl_3$: δ = 7.26 ppm, DMSO-d_6: δ = 2.50 ppm, CD_3CN: δ = 1.94 ppm). Chemical shifts are reported in parts per million (ppm). Coupling constants (*J*) are reported in hertz (Hz). For the assignment of multiplicity to the signals the following abbreviations were used: virt. = virtual, s = singlet, br s = broad singlet, d = doublet, t = triplet, q = quartet, p = pentet, sept = septet, m = multiplet or

unresolved. ^{13}C-NMR spectra were collected on Avance-III (AV-HD300, AV-HD400) NMR systems (Bruker Co.) at 75 or 101 MHz with $CDCl_3$, DMSO-d_6 and CD_3CN as solvents. Chemical shifts were referenced to the residual solvent peak as an internal standard ($CDCl_3$: δ = 77.16 ppm, DMSO-d_6: δ = 39.52 ppm, CD_3CN: δ = 1.32, 118.26 ppm). Reversed high performance liquid chromatography (RP-HPLC) for analytical purposes was performed on a Waters 2695 separations module with a Waters 2996 Photodiode Array Detector at wavelengths between 210 and 600 nm with mobile phases A: H_2O + 0.5% TFA, B: MeCN + 0.5% TFA, C: H_2O, or D: MeCN. Separation was performed on a C18 column (Waters XBridge C18, 3.5 µm, 4.6 x 100 mm). Preparative reversed phase HPLC runs were conducted on a Waters system with a Waters 2545 Quaternary Gradient Module, a Waters 2998 Photodiode Array Detector and a Waters Fraction Collector III with the same mobile phases as in the analytical setup. As stationary phases different C18 columns were used: P1: YMC-Triart C18, 10 x 250 mm, 5 µm; P2: Waters XBridge prep C18 OBDTM, 30 x 150 mm, 5 µm; P3: Waters XBridge prep C18 OBDTM, 50 x 250 mm, 10 µm. High resolution mass spectra were recorded using an LTQ-FT Ultra (Thermo Fisher Scientific Inc.) coupled with a Dionex UltiMate 3000 HPLC system and an ESI or APCI ion source.

4.5 Synthesis

4.5.1 Synthesis of NCA

The synthesis of neocarzilin A (NCA) has been reported previously by us.[1]

4.5.2 Synthesis of NC-4

(*R*)-4-benzyl-3-propionyloxazolidin-2-one (1)

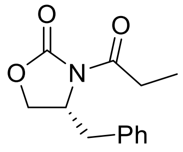

Chemical Formula: $C_{13}H_{15}NO_3$
Molecular Weight: 233.27 g/mol

Under argon atmosphere, propionic acid (5.48 mL, 5.39 g, 72.8 mmol, 1.0 eq) was dissolved in dry THF (130 mL) and dry triethylamine (25.2 mL, 18.4 g, 182 mmol, 2.5 eq) was added. The reaction was cooled to -25 °C and pivaloyl chloride (9.85 mL, 9.56 g, 80.1 mmol, 1.1 eq) was added dropwise. The reaction was stirred at -25 °C for 2 h. Anhydrous lithium chloride (3.39 g, 80.1 mmol, 1.1 eq) and (*R*)-4-benzyl-2-oxazolidinone (12.9 g, 72.8 mmol, 1.0 eq) were added and the reaction was stirred overnight and was allowed to reach room temperature. Ethyl acetate (40 mL) was added and the reaction mixture was washed with H_2O (2 x 40 mL). The combined aqueous layers were extracted with EtOAc (3 x 40 mL). The organic layers were combined, washed with brine (30 mL) and dried over Na_2SO_4. The volatile components were removed under reduced pressure to yield the product (17.1 g, 72.8 mmol, >99%) as a colorless solid.

^{1}H-NMR (400 MHz, $CDCl_3$): δ [ppm] = 7.36 – 7.31 (m, 2H), 7.30 – 7.25 (m, 1H), 7.23 – 7.19 (m, 2H), 4.71 – 4.63 (m, 1H), 4.25 – 4.13 (m, 2H), 3.35 – 3.27 (m, 1H), 3.06 – 2.87 (m, 2H), 2.82 – 2.73 (m, 1H), 1.21 (t, *J* = 7.3 Hz, 3H).

^{13}C-NMR (101 MHz, $CDCl_3$): δ [ppm] = 174.2, 153.7, 135.5, 129.6, 129.1, 127.5, 66.4, 55.3, 38.1, 29.3, 8.4.

The data is in accordace with literature.[13]

(*R*)-4-benzyl-3-((*S*)-2-methylpent-4-ynoyl)oxazolidin-2-one (2)

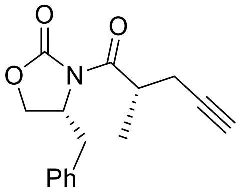

Chemical Formula: $C_{16}H_{17}NO_3$
Molecular Weight: 271.32 g/mol

Lithium diisopropylamide (LDA) was prepared *in situ* by adding *n*-butyllithium (*n*BuLi, 5.60 mL, 2.5 M in hexane, 14.0 mmol, 1.4 eq) to a solution of *N*,*N*'-diisopropylamine (2.16 mL, 15.4 mmol, 1.6 eq), dissolved in dry THF (12 mL), at 0 °C. The reaction was stirred for 30 min at 0 °C and then cooled down to -78 °C. *N*,*N*'-dimethylpropyleneurea (DMPU, 1.21 mL, 10.1 mmol, 1.0 eq) and (*R*)-4-benzyl-3-propionyloxazolidin-2-one (2.27 g, 9.73 mmol, 1 eq), dissolved in dry THF (4 mL) were added. The reaction was stirred for 30 min at -78 °C and propargyl bromide (3.71 mL, 80 wt. % in toluene, 39.2 mmol, 4.0 eq) was added dropwise. The reaction was stirred for 20 h at -78 °C and a saturated aqueous solution of ammonium chloride (20 mL) and water (20 mL) were added. The resulting mixture was extracted with Et_2O (3 x 20 mL). The combined organic phases were washed with water (6 x 10 mL) and dried over anhydrous Na_2SO_4. The volatile components were removed under reduced pressure. Purification by flash chromatography (Hex/EtOAc 3:1 → 1:1) yielded the desired product (911 mg, 3.36 mmol, 35%, *d.r.* = 100:0) as a colorless solid.

TLC: R_f = 0.35 (Hexane/EtOAc = 7:1) [UV, $KMnO_4$]

^{1}H-NMR (400 MHz, $CDCl_3$): δ [ppm] = 7.37 – 7.30 (m, 2H), 7.30 – 7.27 (m, 1H), 7.25 – 7.19 (m, 2H), 4.75 – 4.67 (m, 1H), 4.27 – 4.16 (m, 2H), 3.98 – 3.88 (m, 1H), 3.31 – 3.22 (m, 1H), 2.84 – 2.74 (m, 1H), 2.62 – 2.53 (m, 1H), 2.46 – 2.37 (m, 1H), 1.99 (t, *J* = 2.6 Hz, 1H), 1.33 (d, *J* = 6.9 Hz, 3H).

^{13}C-NMR (101 MHz, $CDCl_3$): δ [ppm] = 175.4, 153.1, 135.3, 129.6, 129.1, 127.6, 81.7, 69.9, 66.4, 55.4, 38.0, 37.7, 22.4, 17.3.

(*S*)-2-methylpent-4-yn-1-ol (3)

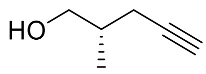

Chemical Formula: $C_6H_{10}O$
Molecular Weight: 98.15 g/mol

MeOH (1.83 mL, 45.2 mmol, 1.8 eq) and $LiBH_4$ (830 mg, 38.1 mmol, 1.5 eq) were added to a solution of compound **2** (6.89 g, 25.4 mol, 1.0 eq) in Et_2O (220 mL) at 0 °C. The reaction was stirred for 5 h at room temperature, $NaOH_{aq}$ (1 M, 25 mL) was added, and the mixture was vigorously stirred at room temperature for 40 min. The organic and aqueous layer were separated, and the aqueous layer was extracted with Et_2O (2 x 25 mL). The combined organic layers were dried over Na_2SO_4. The volatile components were removed under reduced pressure and the residue was purified by flash column chromatography (pentane/Et_2O 5:1 → 1:0) to yield the desired product (2.22 g, 22.6 mmol, 89%) as a yellow oil.

Due to the volatility of the compound, the solvents could not me removed completely.

TLC: R_f = 0.47 (Pentane/Et_2O 1:1) [UV, $KMnO_4$]

^{1}H-NMR: (300 MHz, $CDCl_3$) δ [ppm] = 3.54 (d, *J* = 6.1 Hz, 2H), 2.32 – 2.12 (m, 2H), 1.95 (t, *J* = 2.7 Hz, 1H), 1.92 – 1.79 (m, 1H), 0.98 (d, *J* = 6.9 Hz, 3H).

Synthesis of (*S*)-2-methylpent-4-ynal (4)

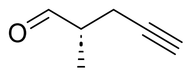

Chemical Formula: C_6H_8O
Molecular Weight: 96.13 g/mol

Oxalyl dichloride (2.14 mL, 2.24 g, 24.9 mmol, 1.1 eq) was dissolved in CH_2Cl_2 (60 mL) and cooled to -78 °C. A solution of DMSO (3.53 mL, 3.89 g, 49.8 mmol, 2.2 eq) and compound **3** (2.22 g, 22.6 mmol, 1.0 eq) in CH_2Cl_2 (15 mL) was added over 45 min. The reaction was stirred for 15 min at -78 °C and NEt_3 (15.8 mL, 11.4 g, 113 mmol, 5 eq) was added dropwise over 15 min. The reaction was stirred for 15 min at -78 °C and the reaction was allowed to reach room temperature within 1 h. Water (150 mL) was added, and the phases separated. The aqueous phase was extracted with CH_2Cl_2 (3 x 50 mL) and the combined organic phases were washed with a HCl_{aq} (2%, 30 mL) and $Na_2CO_{3\,aq}$ (5%, 30 mL). The organic layer was dried over Na_2SO_4, and the volatile components were removed under reduced pressure. Purification by flash column chromatography (Pentane/Et_2O 5:1 → 1:0) yielded the desired product (2.00 g, 20.8 mmol, 92%) as a yellow oil.

TLC: R_f = 0.38 (pentane/Et_2O = 20:1) [UV, $KMnO_4$]

^{1}H-NMR (400 MHz, Chloroform-*d*): δ [ppm] = 9.71 (s, 1H), 2.60 – 2.48 (m, 2H), 2.44 – 2.32 (m, 1H), 2.01 (t, *J* = 2.6 Hz, 1H), 1.24 (d, *J* = 7.1 Hz, 3H).

Ethyl (E)-4-(diethoxyphosphoryl)but-2-enoate (8)

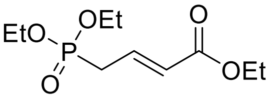

Chemical Formula: $C_{10}H_{19}O_5P$
Molecular Weight: 250.23 g/mol

(*E*)-4-bromobut-2-enoic acid (75%, 10.0 mL, 10.5 g, 63.6 mmol, 1.0 eq) and triethylphosphite (12.5 mL, 12.0 g, 71.9 mmol, 1.1 eq) were stirred at 125 °C for 4 h. The reaction was stirred overnight and allowed to reach room temperature. Purification of the crude product by vacuum distillation yielded the desired product (13.4 g, 53.6 mmol, 84%) as a clear yellowish oil.

^{1}H-NMR (300 MHz, Chloroform-*d*): δ [ppm] = 6.96 – 6.76 (m, 1H), 5.96 (dd, *J* = 15.6, 3.8 Hz, 1H), 4.24 – 4.06 (m, 6H), 2.82 – 2.68 (m, 2H), 1.33 (t, *J* = 6.3 Hz, 6H), 1.31 – 1.26 (m, 3H).

^{13}C-NMR (101 MHz, Chloroform-*d*): δ [ppm] = 165.8, 165.8, 137.6, 137.5, 126.1, 125.9, 62.5, 62.4, 60.6, 31.7, 29.9, 16.6, 16.5, 14.4.

The high complexity of the ^{13}C-NMR results from the P-C coupling.

The data is in accordace with literature.[14]

Ethyl (*S*,2E,4E)-6-methylnona-2,4-dien-8-ynoate (5)

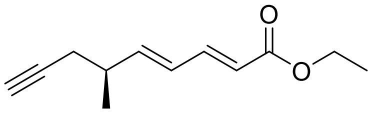

Chemical Formula: $C_{12}H_{16}O_2$
Molecular Weight: 192.26 g/mol

Ethyl (E)-4-(diethoxyphosphoryl)but-2-enoate (7.81 g, 31.2 mmol, 1.5 eq) was dissolved in dry THF (20 mL) and the solution was cooled to -78 °C. LiHMDS (1 M in THF, 31.2 mL, 31.2 mmol, 1.5 eq) was added slowly. The reaction was stirred at -78 °C for 1 h and a solution of (*S*)-2-methylpent-4-ynal (2.0 g, 20.8 mmol, 1.0 eq) in dry THF (10 mL) was added. The reaction was stirred at -78 °C for 1 h and at -40 °C for 3 h and a saturated solution of NH_4Cl_{aq} (100 mL) was added. The phases were separated, and the aqueous phase was extracted with Et_2O (3 x 30 mL). The combined organic phases were washed with brine (20 mL) and dried over Na_2SO_4. The volatile components were removed under reduced pressure and purification of the residue by flash column chromatography (Hex/EtOAc, 40:1) yielded the product (600 mg, 3.12 mmol, 15%, E/Z=87/13) as a clear, slight yellowish oil.

TLC: R_f = 0.5 (Hexane/EtOAc = 9:1) [UV, $KMnO_4$]

^{1}H-NMR (500 MHz, Chloroform-*d*): δ [ppm] = 7.32 – 7.18 (m, 1H), 6.25 – 6.04 (m, 2H), 5.83 (d, *J* = 15.4 Hz, 1H), 4.19 (q, *J* = 7.2 Hz, 2H), 2.57 – 2.47 (m, 1H), 2.32 – 2.22 (m, 2H), 1.99 (t, *J* = 2.7 Hz, 1H), 1.29 (t, *J* = 7.1 Hz, 3H), 1.15 (d, *J* = 6.7 Hz, 3H).

^{13}C-NMR (101 MHz, Chloroform-*d*): δ [ppm] = 147.2, 144.8, 127.6, 120.5, 82.2, 70.0, 60.4, 36.2, 25.8, 19.2, 14.5.

(*S*,2E,4E)-6-methylnona-2,4-dien-8-ynal (6)

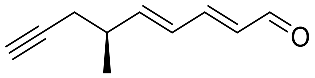

Chemical Formula: $C_{10}H_{12}O$
Molecular Weight: 148.20 g/mol

Ethyl (*S*,2E,4E)-6-methylnona-2,4-dien-8-ynoate (420 mg, 3.1 mmol, 1.0 eq) was dissolved in dry THF (5 mL) and cooled down to -78 °C. DIBAL-H (1 M in hexane, 4.57 mL, 4.58 mmol, 2.1 eq) was added and the reaction stirred at -78 °C for 4 h and was then allowed to reach room temperature. Dry MeOH (6.5 mL) and a saturated, aqueous sodium potassium tartrate solution (19 mL) were slowly added, and the solution was stirred for 3 h at room temperature. Water was added (20 mL) and the resulting solution was extracted with Et_2O (3 x 15 mL). The combined organic phases were washed with brine (50 mL) and dried over Na_2SO_4. The volatile organic compounds were removed under reduced pressure. The crude product was dissolved in hexane (20 mL), MnO_2 (3.79 g, 43.6 mmol, 20 eq) was added and the reaction was stirred for 4.5 h at room temperature. MnO_2 was filtered off and hexane (20 mL) was added to the filtrate. The solution was stirred at room temperature for 30 min and the resulting precipitate was filtered off. The volatile components were removed under reduced pressure to yield the desired product (227 mg, 1.53 mmol, 49% E/Z=88/12) as a yellow oil.

TLC: R_f = 0.17 (Hexane/EtOAc = 30:1) [UV, $KMnO_4$]

^{1}H-NMR (300 MHz, Chloroform-d): δ [ppm] = 9.55 (d, *J* = 8.3 Hz, 1H), 7.09 (dd, *J* = 15.3, 10.1 Hz, 1H), 6.42 – 6.20 (m, 2H), 6.12 (dd, *J* = 15.3, 8.0 Hz, 1H), 2.63 – 2.49 (m, 1H), 2.30 (d, *J* = 2.5 Hz, 1H), 2.01 (t, *J* = 2.6 Hz, 1H), 1.18 (d, *J* = 6.7 Hz, 3H).

^{13}C-NMR (75 MHz, Chloroform-d): δ [ppm] = 193.9, 152.4, 149.6, 131.1, 127.9, 81.9, 70.3, 36.3, 25.6, 19.1.

(*S*,3E,5E,7E)-9-methyldodeca-3,5,7-trien-11-yn-2-one (7)

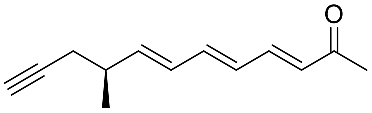

Chemical Formula: $C_{13}H_{16}O$
Molecular Weight: 188.27 g/mol

(*S*,2E,4E)-6-methylnona-2,4-dien-8-ynal (6) (198 mg, 1.34 mmol, 1.0 eq) was dissolved in toluene (10 mL) and 1-(triphenylphosphoraniliden)-2-propanone (850 mg, 262 mmol, 2.0 eq) was added. The mixture was heated to 100 °C and stirred overnight. The reaction was cooled to room temperature and hexane (10 mL) was added. The resulting precipitate was filtered off and the volatile components of the liquor were removed under reduced pressure. Hexane (15 mL) was added to the residue and the mixture was stirred for 30 min and filtered. The volatile compounds of the liquor were removed under reduced pressure. The crude product was purified by flash column chromatography (Hex/EtOAc, 20:1) to yield the product (160 mg, 851 µmol, 64%) as a yellow oil.

TLC: R_f = 0.24 (Hexane/EtOAc = 10:1) [UV, $KMnO_4$]

HRMS APCI calcd. for $C_{13}H_{17}O$ $[M+H^+]^+$: 189.12739 , found 189.12739.

^{1}H-NMR (400 MHz, Chloroform-d): δ [ppm] = 7.14 (dd, *J* = 15.5, 11.1 Hz, 1H), 6.58 (dd, *J* = 14.9, 10.7 Hz, 1H), 6.32 – 6.16 (m, 2H), 6.13 (d, *J* = 15.5 Hz, 1H), 5.94 (dd, *J* = 15.2, 7.3 Hz, 1H), 2.57 – 2.45 (m, 1H), 2.29 – 2.23 (m, 5H), 2.00 (t, *J* = 2.6 Hz, 1H), 1.15 (d, *J* = 6.8 Hz, 3H).

^{13}C-NMR (126 MHz, Chloroform-d): δ [ppm] = 198.6, 143.7, 143.6, 141.8, 130.0, 129.4, 129.1, 82.4, 70.0, 36.2, 27.5, 26.0, 19.4.

(*S*,3Z,5E,7E,9E)-1,1,1-trichloro-4-hydroxy-11-methyltetradeca-3,5,7,9-tetraen-13-yn-2-one (NC-4)

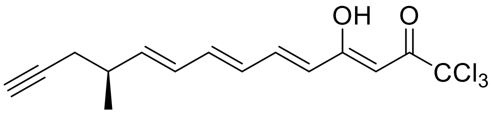

Chemical Formula: $C_{15}H_{15}Cl_3O_2$
Molecular Weight: 333.63 g/mol

(*S*,3E,5E,7E)-9-methyldodeca-3,5,7-trien-11-yn-2-one (7) (75.0 mg, 398 µmol, 1.0 eq) was dissolved in 3.5 mL dry THF und cooled to -78 °C. LiHMDS (816 µL, 1.00 M, 817 µmol, 2.1 eq) was added dropwise and the mixture was stirred for 1 h at -78 °C. Trichloroacetic anhydride (80.0 µL, 438 µmol, 1.0 eq) was added and the reaction was stirred for 1.5 h at -78 °C before additional trichloroacetic anhydride (44.0 µL, 241 µmol, 0.55 eq) was added. PBS (5 mL) was added, and the mixture was extracted with Et_2O (3 x 10 mL). The combined organic phases were washed with brine (20 mL) and dried with Na_2SO_4. The volatile components were removed under reduced pressure and crude product was purified my preparative reversed face chromatography, followed by flash column chromatography (PE/Et_2O, 9:1). This yielded the product (22 mg, 65.9 µmol, 16.5%) as a yellow solid.

TLC: R_f = 0.45 (Hexane/EtOAc = 10:1) [UV, $KMnO_4$]

HRMS ESI calcd. for $C_{15}H_{14}Cl_3O_2$ $[M-H^+]^-$: 331.00649, found 331.00646.

^{1}H-NMR (500 MHz, Chloroform-d): δ [ppm] = 13.37 (s, 1H), 7.39 – 7.33 (m, 1H), 6.66 – 6.54 (m, 1H), 6.35 – 6.27 (m, 1H), 6.26 – 6.19 (m, 1H), 6.16 (s, 1H), 6.06 – 5.92 (m, 2H), 2.60 – 2.46 (m, 1H), 2.34 – 2.20 (m, 2H), 2.00 (t, *J* = 2.6 Hz, 1H), 1.16 (d, *J* = 6.8 Hz, 3H).

^{13}C-NMR (126 MHz, Chloroform-d): δ [ppm] = 186.3, 177.9, 144.6, 142.6, 142.3, 129.4, 129.2, 123.9, 95.3, 93.5, 82.3, 70.0, 36.3, 30.5, 25.9, 19.4.

5 NMR Spectra

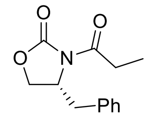

Chemical Formula: $C_{13}H_{15}NO_3$
Molecular Weight: 233.27 g/mol

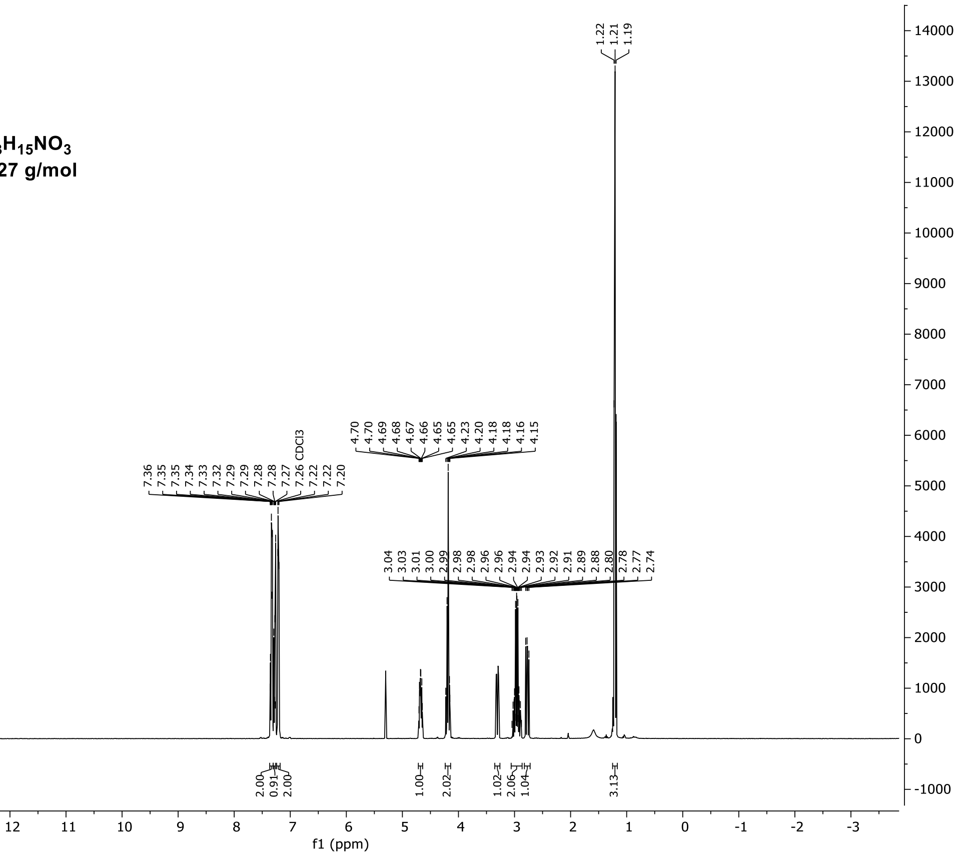

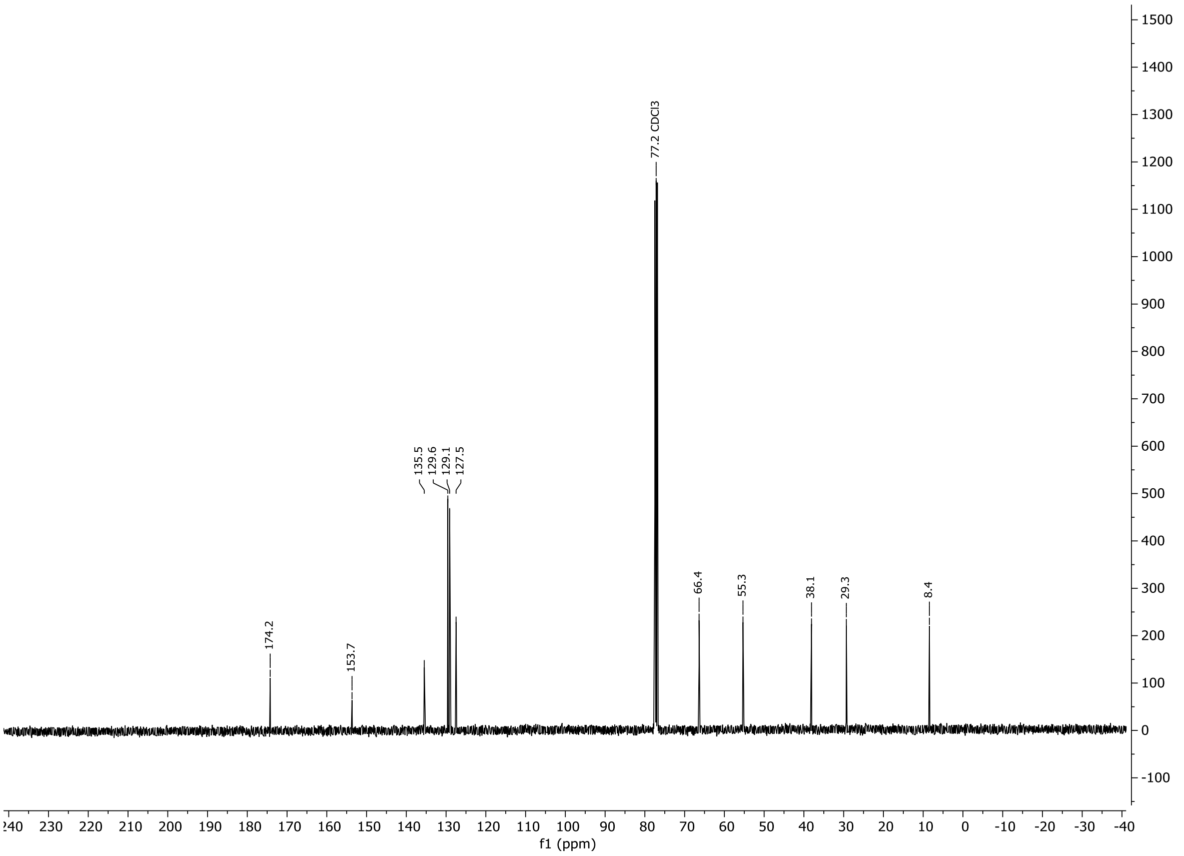

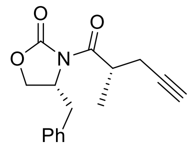

Chemical Formula: $C_{16}H_{17}NO_3$
Molecular Weight: 271.32 g/mol

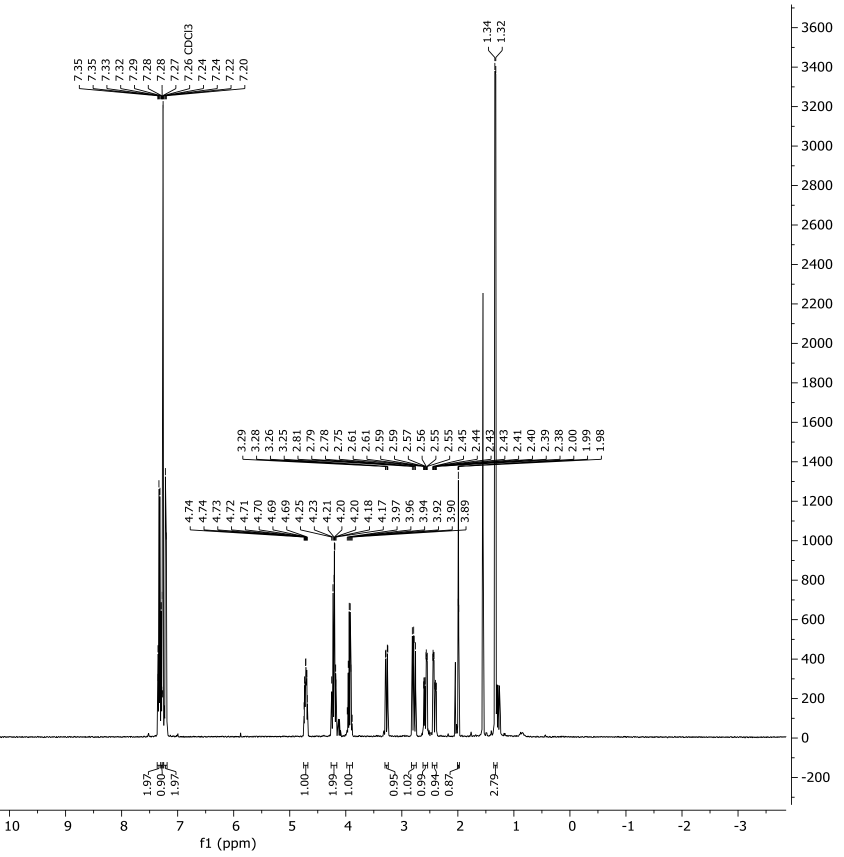

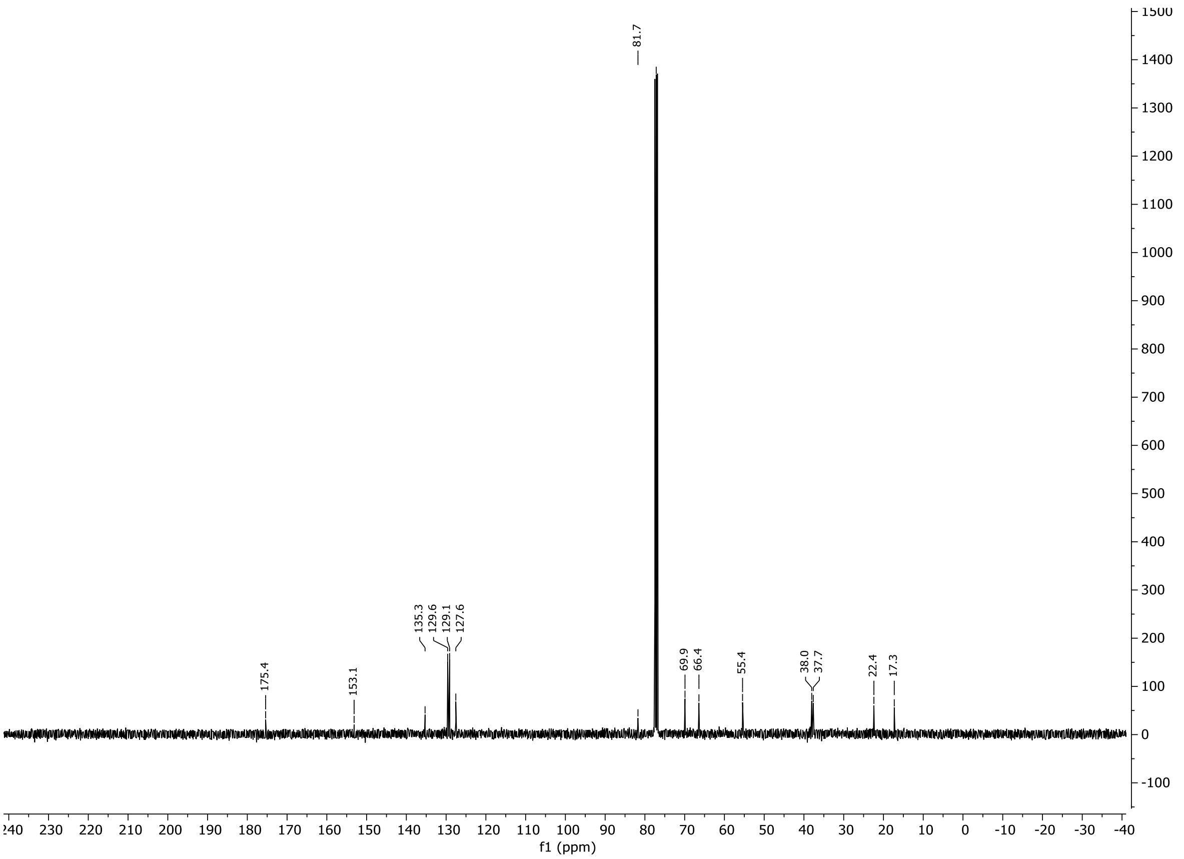

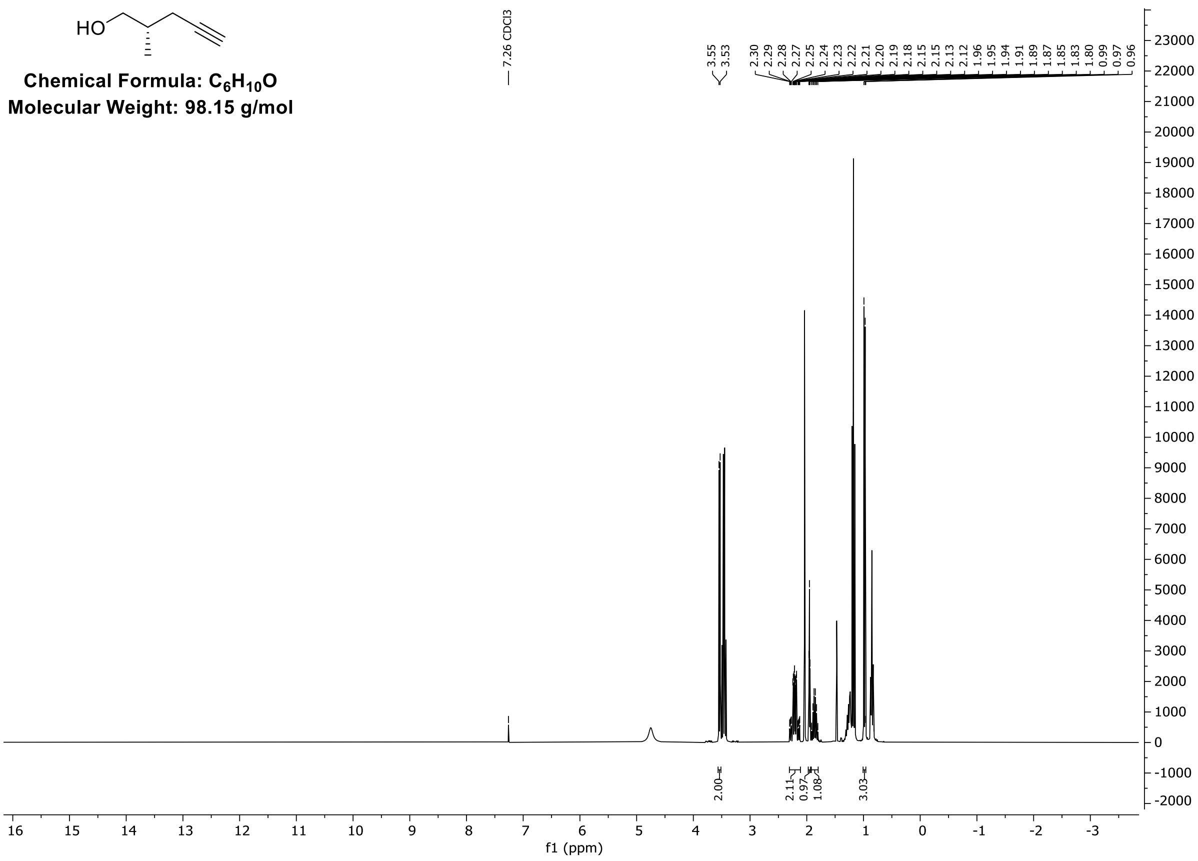
HO
Chemical Formula: C6H10O
Molecular Weight: 98.15 g/mol
7.26 CDCl3
3.55
3.53
2.30
2.29
2.28
2.27
2.25
2.24
2.23
2.22
2.21
2.20
2.19
2.18
2.15
2.15
2.13
2.12
1.96
1.95
1.94
1.91
1.89
1.87
1.85
1.83
1.80
0.99
0.97
0.96
2.00
2.11
0.97
1.08
3.03
f1 (ppm)

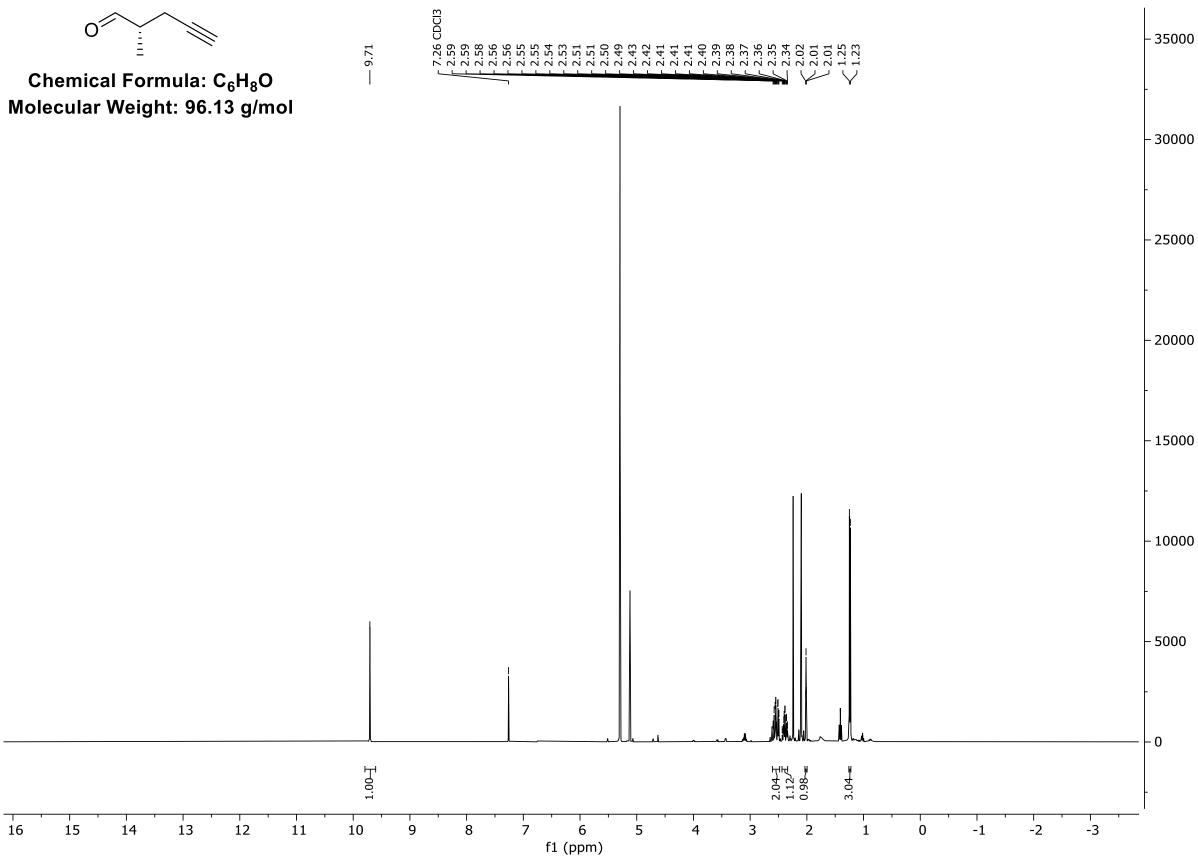
O
Chemical Formula: C6H8O
Molecular Weight: 96.13 g/mol
9.71
7.26 CDCl3
2.59
2.59
2.58
2.56
2.56
2.55
2.55
2.54
2.53
2.51
2.51
2.50
2.49
2.43
2.42
2.41
2.41
2.40
2.39
2.38
2.37
2.36
2.35
2.34
2.02
2.01
2.01
1.25
1.23
1.00
2.04
1.12
0.98
3.04
f1 (ppm)

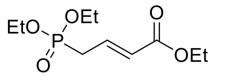

Chemical Formula: $C_{10}H_{19}O_5P$
Molecular Weight: 250.23 g/mol

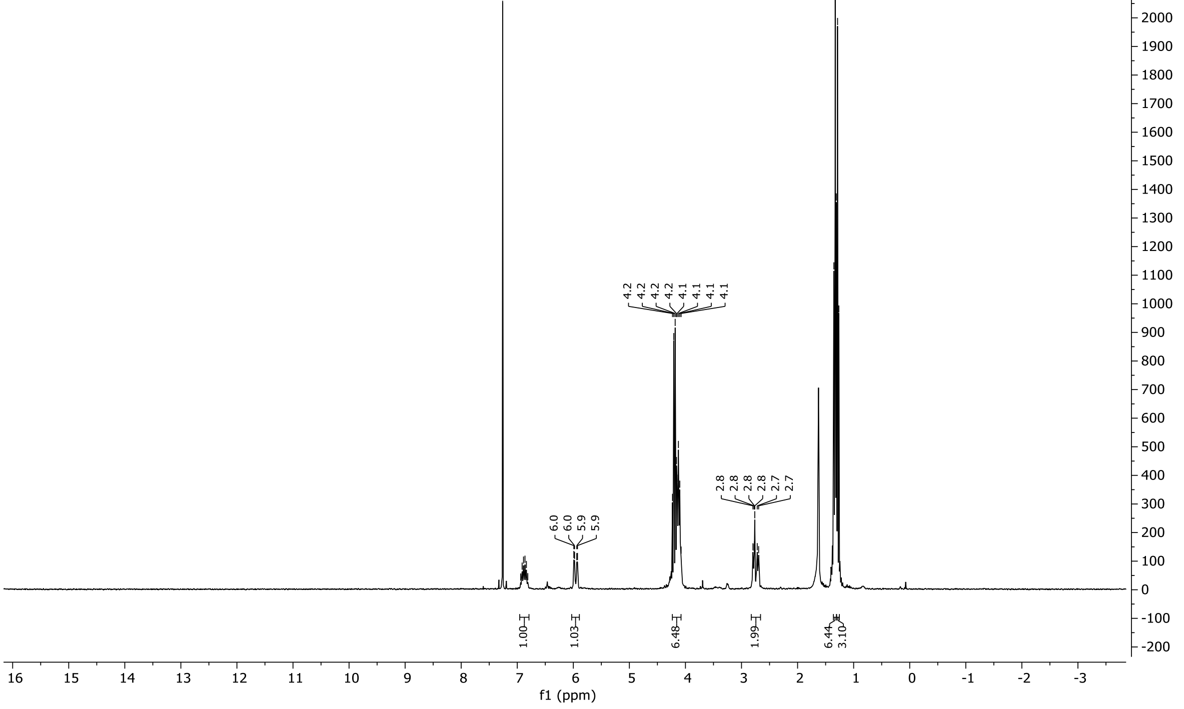

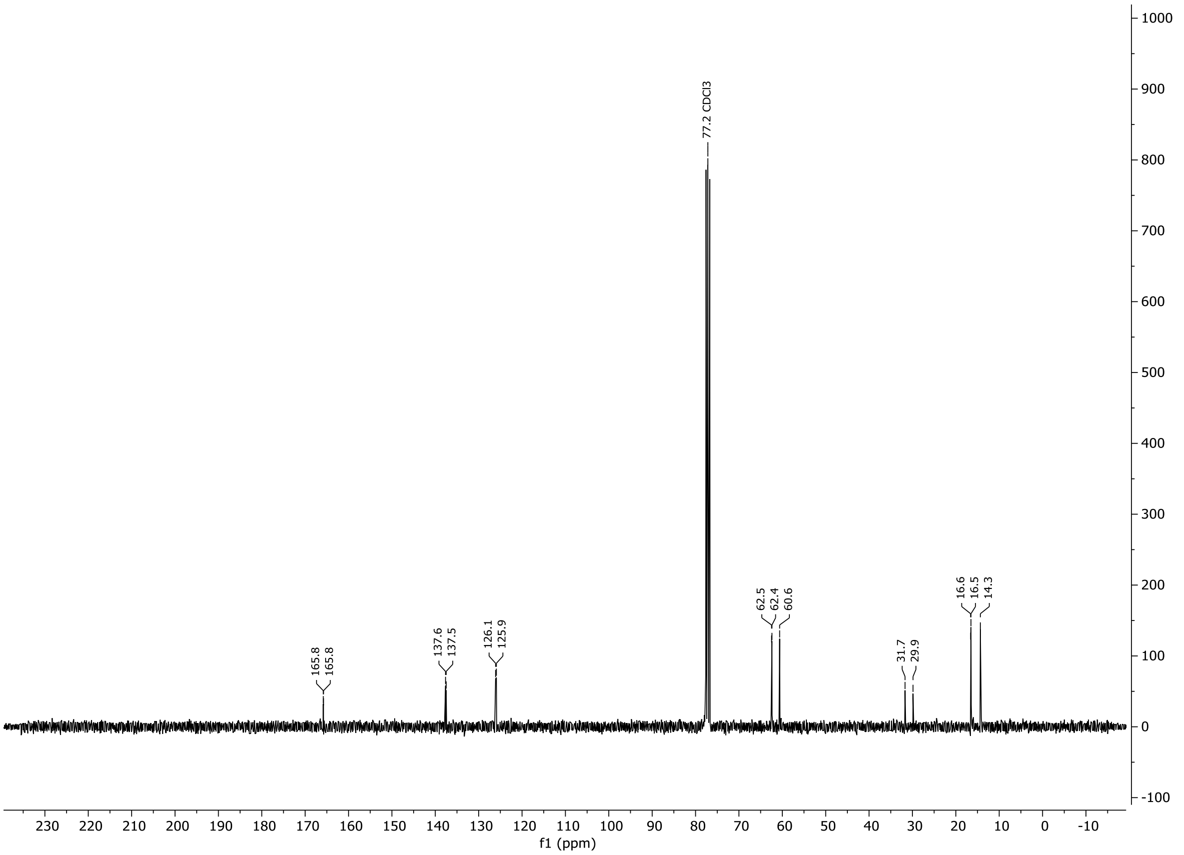

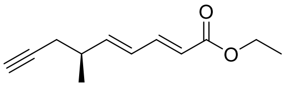

Chemical Formula: $C_{12}H_{16}O_2$
Molecular Weight: 192.26 g/mol

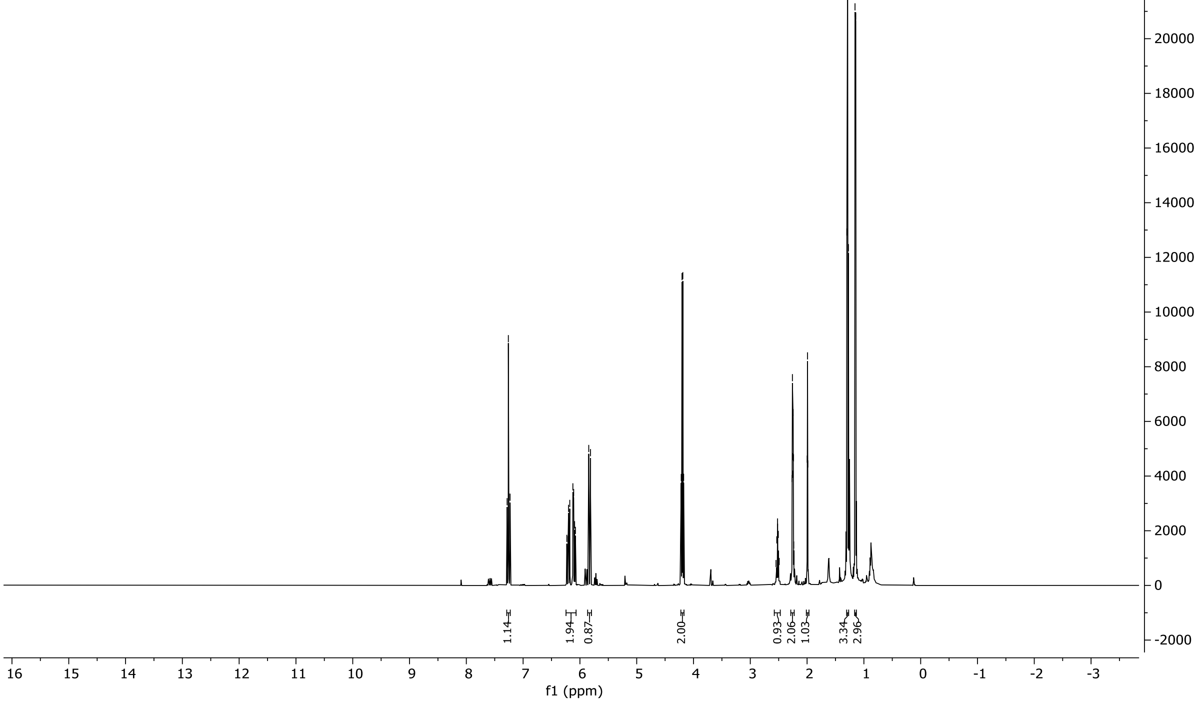

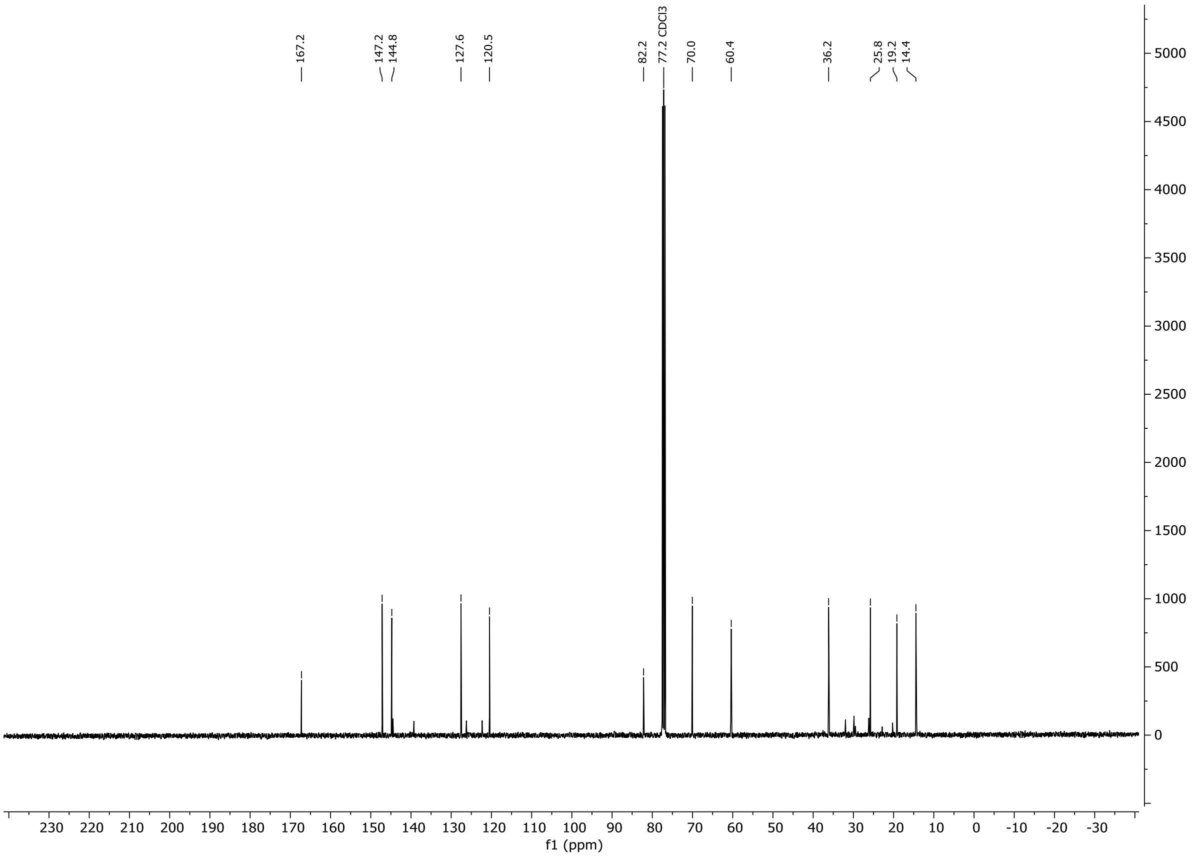

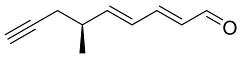

Chemical Formula: $C_{10}H_{12}O$
Molecular Weight: 148.20 g/mol

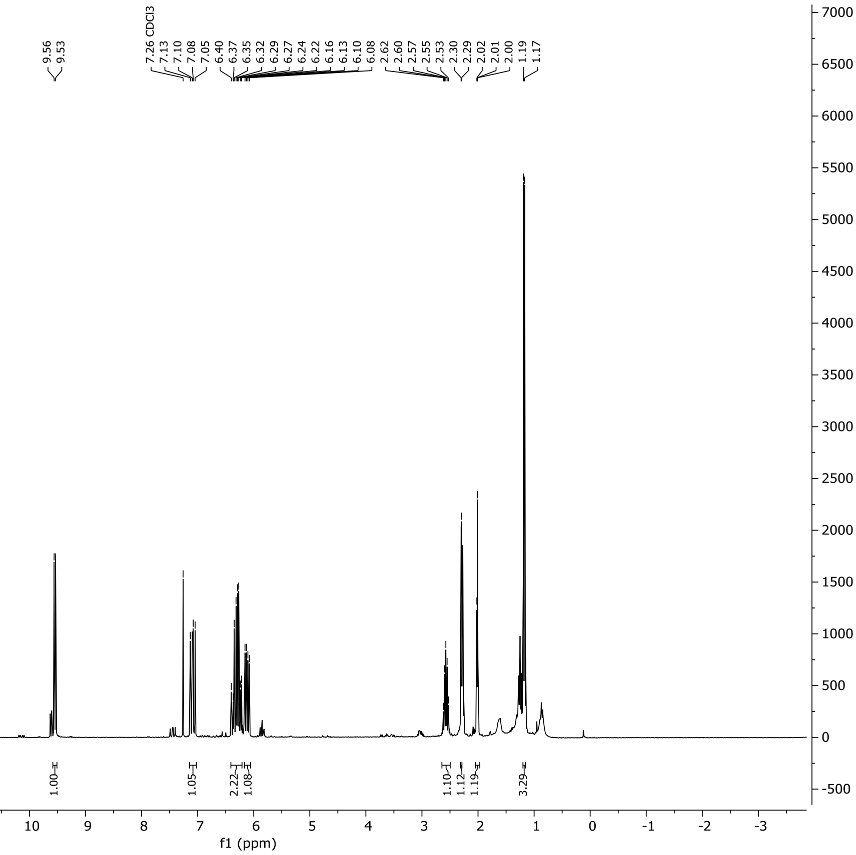

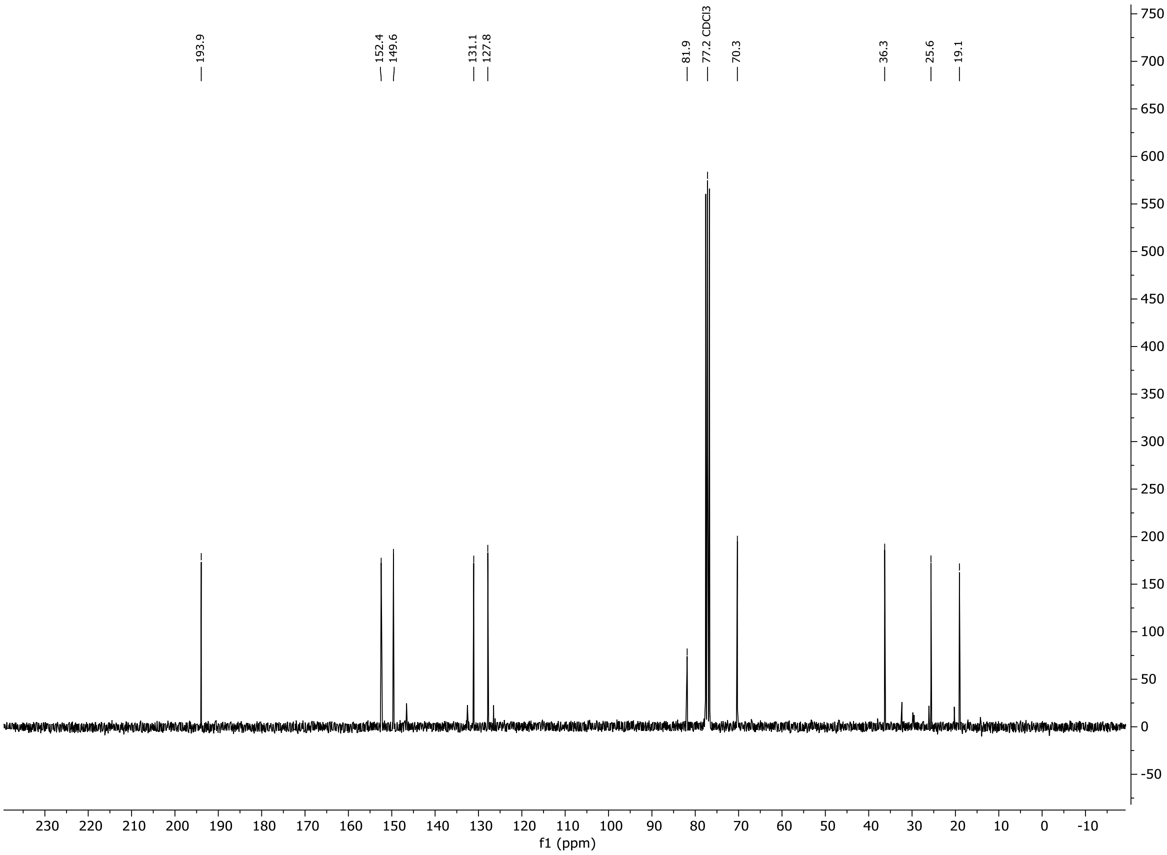

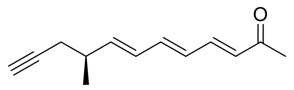

Chemical Formula: $C_{13}H_{16}O$
Molecular Weight: 188.27 g/mol

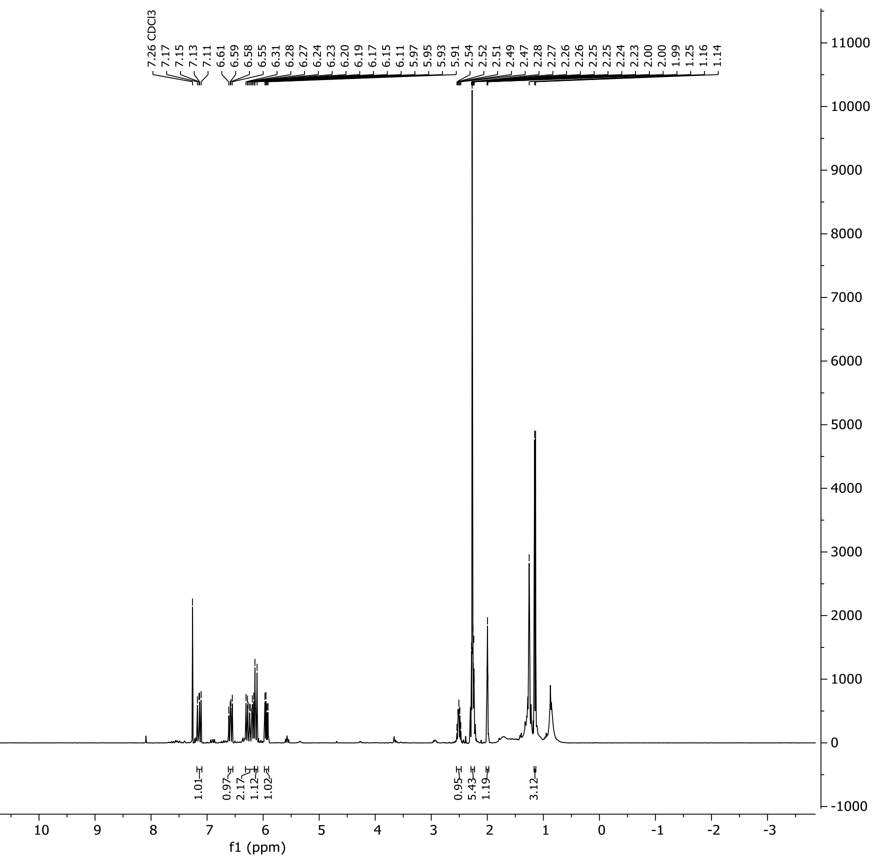

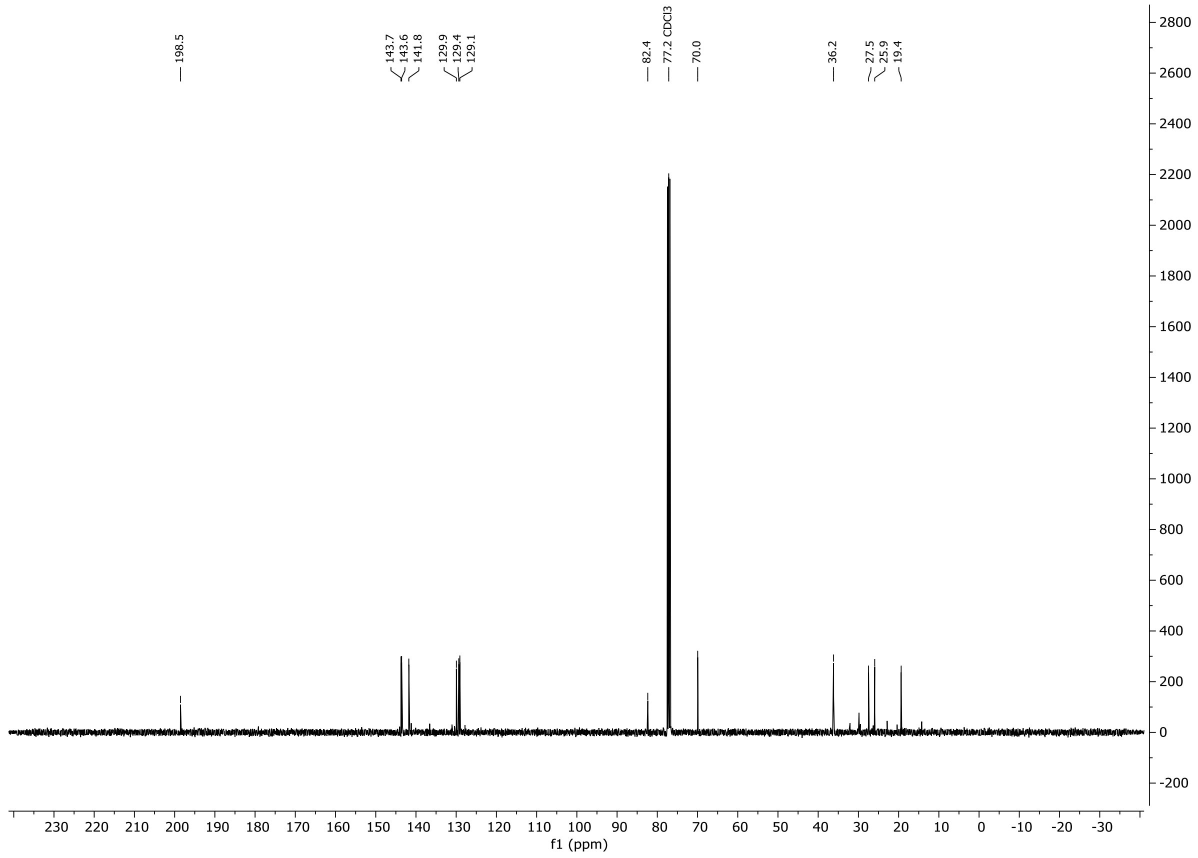

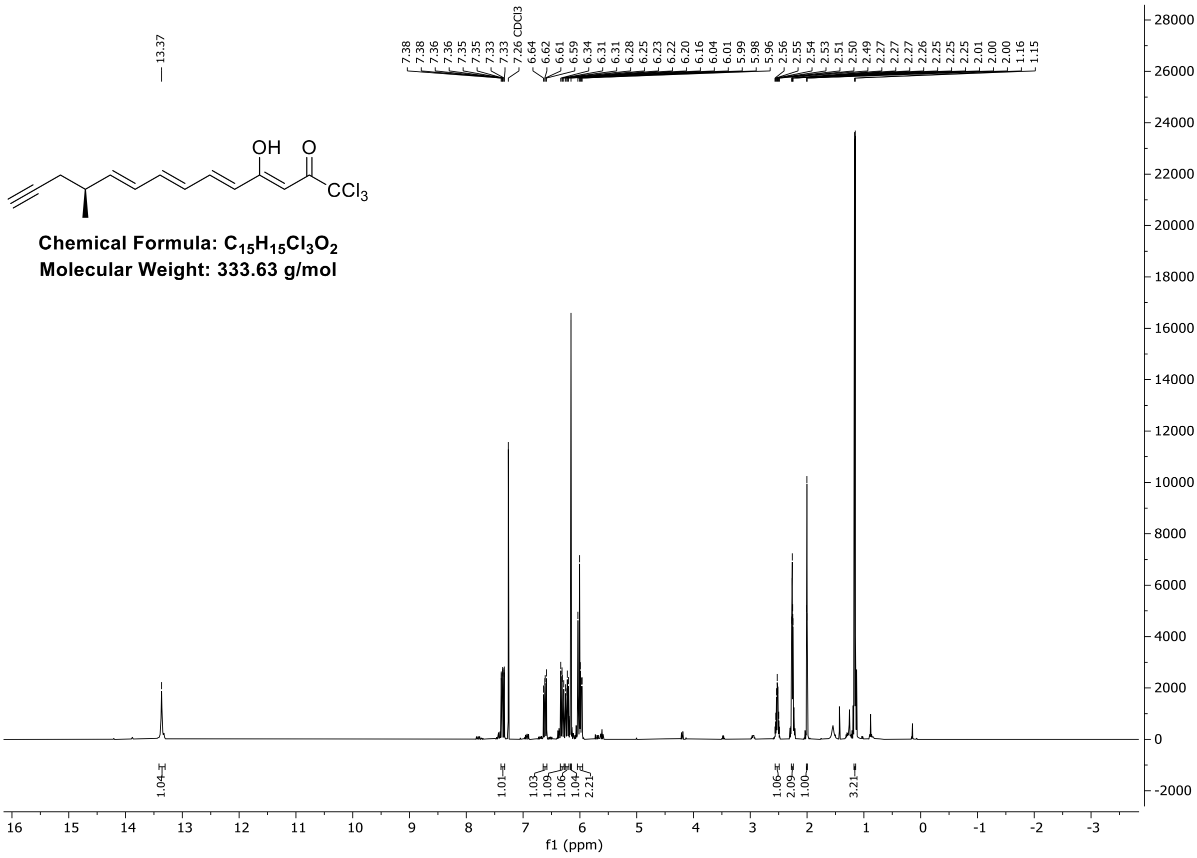
OH O
CCl3
Chemical Formula: $C_{15}H_{15}Cl_3O_2$
Molecular Weight: 333.63 g/mol
13.37
7.38 7.38 7.36 7.36 7.35 7.35 7.33 7.33 7.26 CDCl3 6.64 6.62 6.61 6.59 6.34 6.31 6.31 6.28 6.25 6.23 6.22 6.20 6.16 6.04 6.01 5.99 5.98 5.96 2.56 2.55 2.54 2.53 2.51 2.50 2.49 2.27 2.27 2.27 2.26 2.25 2.25 2.25 2.01 2.00 2.00 1.16 1.15
1.04 1.01 1.03 1.09 1.06 1.04 2.21 1.06 2.09 1.00 3.21
f1 (ppm)

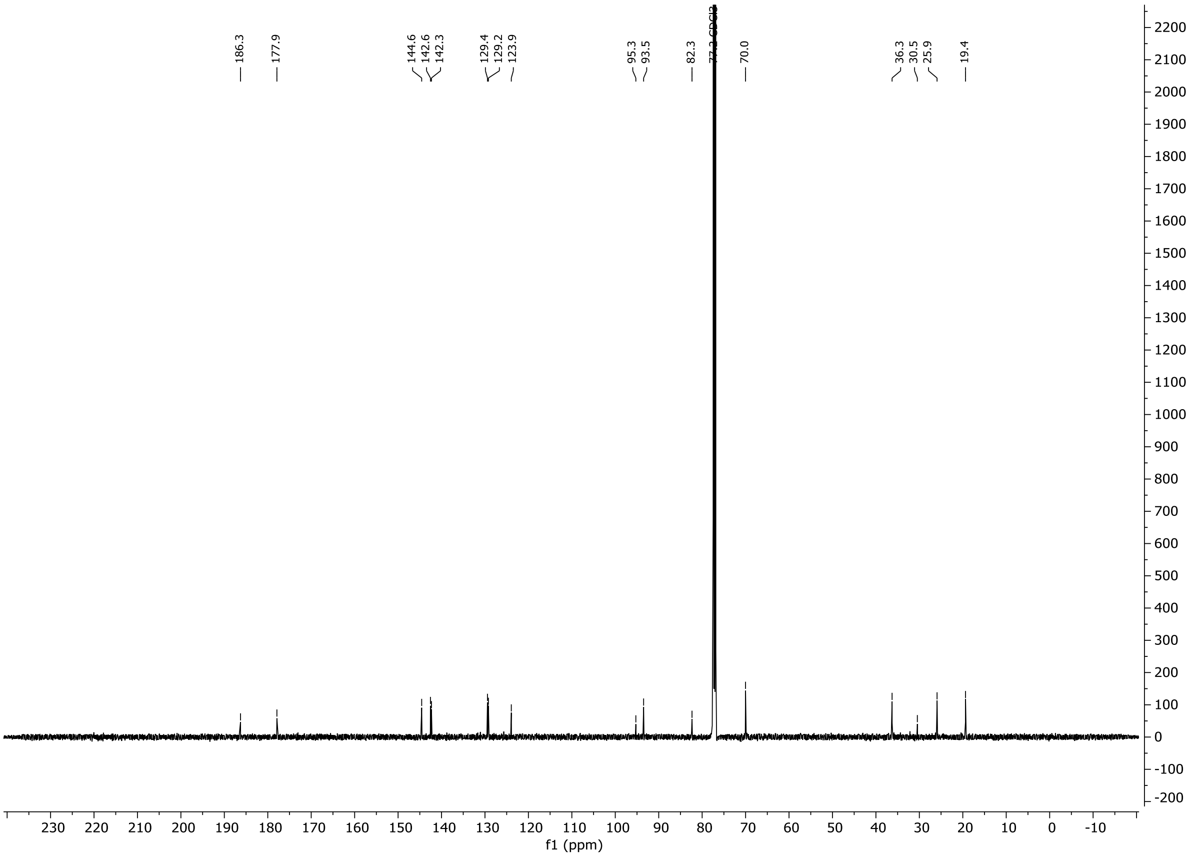
186.3 177.9 144.6 142.6 142.3 129.4 129.2 123.9 95.3 93.5 82.3 77.2 CDCl3 70.0 36.3 30.5 25.9 19.4
f1 (ppm)

6 Safety Statement

All experiments were performed according to standard laboratory safety rules. No unexpected or unusually high safety hazards were encountered.

7 Supplementary References

(1) Gleissner, C. M.; Pyka, C. L.; Heydenreuter, W.; Gronauer, T. F.; Atzberger, C.; Korotkov, V. S.; Cheng, W.; Hacker, S. M.; Vollmar, A. M.; Braig, S.; et al. Neocarzilin A Is a Potent Inhibitor of Cancer Cell Motility Targeting VAT-1 Controlled Pathways. *ACS Cent Sci* **2019**, *5* (7), 1170-1178. DOI: 10.1021/acscentsci.9b00266.
(2) Cox, J.; Mann, M. MaxQuant enables high peptide identification rates, individualized p.p.b.-range mass accuracies and proteome-wide protein quantification. *Nat Biotechnol* **2008**, *26* (12), 1367-1372. DOI: 10.1038/nbt.1511.
(3) Cox, J.; Neuhauser, N.; Michalski, A.; Scheltema, R. A.; Olsen, J. V.; Mann, M. Andromeda: A Peptide Search Engine Integrated into the MaxQuant Environment. *J Proteome Res* **2011**, *10* (4), 1794-1805. DOI: 10.1021/pr101065j.
(4) Cox, J.; Hein, M. Y.; Luber, C. A.; Paron, I.; Nagaraj, N.; Mann, M. Accurate Proteome-wide Label-free Quantification by Delayed Normalization and Maximal Peptide Ratio Extraction, Termed MaxLFQ. *Mol Cell Proteomics* **2014**, *13* (9), 2513-2526. DOI: 10.1074/mcp.M113.031591.
(5) Vizcaino, J. A.; Deutsch, E. W.; Wang, R.; Csordas, A.; Reisinger, F.; Rios, D.; Dianes, J. A.; Sun, Z.; Farrah, T.; Bandeira, N.; et al. ProteomeXchange provides globally coordinated proteomics data submission and dissemination. *Nature Biotechnology* **2014**, *32* (3), 223-226. DOI: 10.1038/nbt.2839.
(6) Tyanova, S.; Temu, T.; Sinitcyn, P.; Carlson, A.; Hein, M. Y.; Geiger, T.; Mann, M.; Cox, J. The Perseus computational platform for comprehensive analysis of (prote)omics data. *Nat Methods* **2016**, *13* (9), 731-740. DOI: 10.1038/Nmeth.3901.
(7) Kramer, A.; Green, J.; Pollard, J., Jr.; Tugendreich, S. Causal analysis approaches in Ingenuity Pathway Analysis. *Bioinformatics* **2014**, *30* (4), 523-530. DOI: 10.1093/bioinformatics/btt703.
(8) Zecha, J.; Satpathy, S.; Kanashova, T.; Avanessian, S. C.; Kane, M. H.; Clauser, K. R.; Mertins, P.; Carr, S. A.; Kuster, B. TMT Labeling for the Masses: A Robust and Cost-efficient, In-solution Labeling Approach. *Mol Cell Proteomics* **2019**, *18* (7), 1468-1478. DOI: 10.1074/mcp.TIR119.001385.
(9) Ruprecht, B.; Zecha, J.; Zolg, D. P.; Kuster, B. High pH Reversed-Phase Micro-Columns for Simple, Sensitive, and Efficient Fractionation of Proteome and (TMT labeled) Phosphoproteome Digests. *Methods Mol Biol* **2017**, *1550*, 83-98. DOI: 10.1007/978-1-4939-6747-6_8.
(10) Ruprecht, B.; Koch, H.; Medard, G.; Mundt, M.; Kuster, B.; Lemeer, S. Comprehensive and reproducible phosphopeptide enrichment using iron immobilized metal ion affinity chromatography (Fe-IMAC) columns. *Mol Cell Proteomics* **2015**, *14* (1), 205-215. DOI: 10.1074/mcp.M114.043109.
(11) Hahne, H.; Pachl, F.; Ruprecht, B.; Maier, S. K.; Klaeger, S.; Helm, D.; Medard, G.; Wilm, M.; Lemeer, S.; Kuster, B. DMSO enhances electrospray response, boosting sensitivity of proteomic experiments. *Nat Methods* **2013**, *10* (10), 989-991. DOI: 10.1038/nmeth.2610.
(12) Ran, F. A.; Hsu, P. D.; Wright, J.; Agarwala, V.; Scott, D. A.; Zhang, F. Genome engineering using the CRISPR-Cas9 system. *Nat Protoc* **2013**, *8* (11), 2281-2308. DOI: 10.1038/nprot.2013.143.

(13) Lu, H.; Handore, K. L.; Wood, T. E.; Shimokura, G. K.; Schimmer, A. D.; Batey, R. A. Total Synthesis of the 2,5-Disubstituted γ-Pyrone E1 UAE Inhibitor Himeic Acid A. *Organic Letters* **2023**, *25* (41), 7502-7506. DOI: 10.1021/acs.orglett.3c02761.
(14) O'Boyle, N. M.; Barrett, I.; Greene, L. M.; Carr, M.; Fayne, D.; Twamley, B.; Knox, A. J. S.; Keely, N. O.; Zisterer, D. M.; Meegan, M. J. Lead Optimization of Benzoxepin-Type Selective Estrogen Receptor (ER) Modulators and Downregulators with Subtype-Specific ERα and ERβ Activity. *Journal of Medicinal Chemistry* **2018**, *61* (2), 514-534. DOI: 10.1021/acs.jmedchem.6b01917.
